# Continuous Morphological Variation Correlated with Genome Size Indicates Frequent Introgressive Hybridization among *Diphasiastrum* Species (Lycopodiaceae) in Central Europe

**DOI:** 10.1371/journal.pone.0099552

**Published:** 2014-06-16

**Authors:** Kristýna Hanušová, Libor Ekrt, Petr Vít, Filip Kolář, Tomáš Urfus

**Affiliations:** 1 Department of Botany, Charles University, Praha, Czech Republic; 2 Department of Botany, University of South Bohemia, České Budějovice, Czech Republic; 3 Institute of Botany, Academy of Sciences of the Czech Republic, Průhonice, Czech Republic; University of Idaho, United States of America

## Abstract

Introgressive hybridization is an important evolutionary process frequently contributing to diversification and speciation of angiosperms. Its extent in other groups of land plants has only rarely been studied, however. We therefore examined the levels of introgression in the genus *Diphasiastrum*, a taxonomically challenging group of Lycopodiophytes, using flow cytometry and numerical and geometric morphometric analyses. Patterns of morphological and cytological variation were evaluated in an extensive dataset of 561 individuals from 57 populations of six taxa from Central Europe, the region with the largest known taxonomic complexity. In addition, genome size values of 63 individuals from Northern Europe were acquired for comparative purposes. Within Central European populations, we detected a continuous pattern in both morphological variation and genome size (strongly correlated together) suggesting extensive levels of interspecific gene flow within this region, including several large hybrid swarm populations. The secondary character of habitats of Central European hybrid swarm populations suggests that man-made landscape changes might have enhanced unnatural contact of species, resulting in extensive hybridization within this area. On the contrary, a distinct pattern of genome size variation among individuals from other parts of Europe indicates that pure populations prevail outside Central Europe. All in all, introgressive hybridization among *Diphasiastrum* species in Central Europe represents a unique case of extensive interspecific gene flow among spore producing vascular plants that cause serious complications of taxa delimitation.

## Introduction

Hybridization among related taxa has a range of possible biological consequences: from the production of sterile offspring, through introgression of alleles into populations, to the formation of new entities [1], [2]. According to ploidy level of participating parental accessions two types of hybridization are known – homoploid hybridization (equal ploidy level) and heteroploid hybridization (different ploidy level). Following text concerns only homoploid type.

Three different levels of homoploid hybridization may be distinguished. The most frequent is hybridization between two well delimited species (with developed hybridization barriers) producing sterile F_1_ hybrids. On the other hand, evolutionarily younger and dynamic plant species often produce fertile hybrids that further contribute to the evolutionary dynamics of populations and lineages. The extreme form of hybridization is introgression (i.e. intense and repeated gene flow across a weak species border via numerous backcrosses), which can lead to a highly complicated situation with collapsed reproductive barriers, frequently manifested as reticulate hybrid swarms [1], [3]–[6]. Finally, constant gene flow through introgression can result in confusing taxonomic patterns, threats of extinction of rare species *via* genetic erosion. It can, however, also lead to novel genotypes and changes in adaptive traits [7]–[10].

Introgressively hybridizing populations are known in many groups of angiosperms [1], [11]–[13] but are very unusual in ferns (monilophytes), where F1 hybrids of sexual species are believed to be completely sterile in nearly all cases [12], [14], [15]. However, production of viable spores is thought to be relatively common in hybrids of the lycopod genera *Diphasiastrum* Holub, *Lycopodiella* Holub and *Lycopodium* L. [14], [16], [17]. Their hybrids are considered to be stabilized hybrids with normal meiosis. Backcrosses and introgressive hybridization have not been detected, and the stabilization of large clusters of hybrid shoots has been attributed to the strong cloning ability of these taxa [14], [18]. Generally, such events may cause taxonomic confusion and numerous misinterpretations in practical determination (taxon identification) [19].

The genus *Diphasiastrum* Holub (*Lycopodium* sect. *Complanata* Victorin) with 20–30 species is the world's largest and taxonomically most complex group within the Lycopodiaceae family [17]. Its species are widely distributed across the Northern Hemisphere with several occurrences in tropical highlands. Their base chromosome number is generally accepted to be x = 23 and polyploidy (3x and 4x) is extremely rare (e.g. [14], [18], [20]). Six diploid taxa are commonly recognized in Europe: three (basic) species – *D. alpinum* (L.) Holub, *D. tristachyum* (Pursh) Holub and *D. complanatum* (L.) Holub – and morphologically intermediate hybrids (formally labelled as intermediate species and currently treated as predominantly recent hybrids and, in some regions, isolated hybridogenous lineages) *D. ×issleri* (Rouy) Holub *(D. complanatum* × *D. alpinum*), *D. ×oellgaardii* Stoor *et al.* (*D. alpinum* × *D. tristachyum*) and *D. ×zeilleri* (Rouy) Holub (*D. complanatum × D. tristachyum*) [18], [20]–[24]. Especially complicated is the situation in regions where all six taxa co-occur. Mixed populations consisting of four, five or all six taxa have been detected in Central Europe, for example SE France (Vosges Mts; [23], [25]), Austria (Bavarian forest; [26]), Germany ([26], [27]) and the Czech Republic (Šumava Mts, Krkonoše Mts, Jeseníky Mts; [28]–[33]). In the rest of Europe, by contrast, *Diphasiastrum* taxa occur mostly allopatrically (e.g. [34]–[38]). From the point of view of practical determination, they are considered a taxonomically critical group [12], [39], [40]. In contrast to the thoroughly investigated populations from Western and Northern Europe, the putatively most complex Central European hybrid zone has not been studied in sufficient detail. European *Diphasiastrum* taxa are generally stress-tolerant plants that avoid high-competition habitats, especially unforested ones. Basic species occur either in tundra-type habitats (incl. alpine mountain zones) and occasionally in open forest sites [27], [29], [41]. On the contrary, hybrid taxa tend to occur in man-disturbed habitats such as periodically heavily disturbed ski slopes, timber storage places, forest glades, deforested strips and road margins, where basic species frequently co-occur [29]. Incidentally, all localities with sympatric occurrence of 3 and more taxa (both, basic and hybrid) are known from such type of habitat (e.g. [29], [41]). For example, *D. ×oellgaardii* has been described from a ski slope, a typical secondary habitat, where it co-occurred with several other *Diphasiastrum* taxa [23], [42].

A number of factors complicate investigations of hybridization patterns in *Diphasiastrum*. Members of this genus have a simple morphology with few discrete morphological features that can be evaluated [21], [43], [44]. Lycopods are also characterized by having two independent stages of life cycle – green, photosynthetic, diploid sporophytes (asexual generation) and underground, heterotrophic and long-lived haploid gametophytes (sexual generation). Sexual reproduction is restricted to gametophyte thus the hybridization is truly obscure and cryptic [16]. Also the mycorrhizal dependence of the gametophyte makes their spores difficult to germinate in controlled laboratory environments [45], and crossing experiments are virtually impossible to accomplish [24]. Large-scale *in situ* screening for various morphological, cytological or genetic traits thus seems to be the most achievable way to investigate gene flow and reproductive interactions within the group.

Various methodological approaches are available for studying introgressive hybridization (morphometrics, karyology, allozymes, microsatellites [1], [4], [46]–[49]. Flow cytometry represents a rather dated but still very efficient tool for the study of hybridization (including introgression), as it allows for rapid estimation of nuclear DNA content of large numbers of individuals [50]. DNA content is largely stable at the species level [51]–[59], and hybrid individuals can easily be detected by their intermediate genome size [60]–[64]. Although plant hybridization studies frequently employ morphometrics and flow cytometry, only a handful of them examine correlations between morphology and genome size using a large enough dataset subjected to a robust statistical evaluation [60], [63], [65]. Importantly, absolute genome size of diploid *Diphasiastrum* taxa has been demonstrated to be a taxonomically specific marker that allows detection of hybrid individuals [20], [32], [33].

The patterns of hybridization in *Diphasiastrum* have recently been addressed using two types of markers: low-copy nuclear genes and genome size. Sequences of three regions of nuclear genome (RPB2, LEAFY, LAMB4) confirmed the hybrid status of *D. ×issleri*, *D. ×oellgaardii* and *D. ×zeilleri* [18], [24]. This study of a limited sample set also indicates that certain levels of recent hybridization and backcrossing exist within European *Diphasiastrum*, however, leaving unknown its frequency and variation patterns in natural populations. On the contrary, discrete variation in genome size in several parts of Europe indicates only primary hybridization with no hint of backcrossing (except for a few rare triploid hybrids) or introgression [20]. Nevertheless, as introgression leads to continuous patterns of variation in species traits (including genome size; [61], sufficiently large and carefully designed sampling is crucial for its discovery. It is thus possible that the levels of introgressive hybridization could have been underestimated because of the generally low number of individuals sampled (165), few samples per population studied (mean 1.62, range 1–9) and very limited sampling within the taxonomically most complex region of Central Europe, where all species co-occur [20].

In order to comprehensively evaluate the frequency and patterns of hybridization in the model lycopod group of *Diphasiastrum*, we conducted a study targeted at the taxonomically most challenging area of Central Europe using two independent markers for interspecific variation that allow large-scale screens: genome size and morphology (both numerical and geometric morphometrics). In one part of Central Europe (the Czech Republic and its immediate vicinity), we exhaustively collected rich samples of all known populations. For comparative purposes, we also screened for genome size variation (and morphological features) within two other European regions (Scandinavia and the British Isles) with less complex and largely allopatric distribution of the species. We asked the following specific questions (i) Does genome size correlate with morphological variation? (ii) What is the pattern of morphological and genome size variation among *Diphasiastrum* individuals in Central Europe? Do the six taxa represent distinct morphological or cytological entities? (iii) Are populations from Central Europe uniform in their genome size and morphology, or do they rather consist of individuals that are variable in these traits? (iv) Is there any difference in the pattern of genome size variation between Central Europe and comparable areas?

## Material and Methods

### Sampling design

We thank the administration of the Krkonoše and Šumava National Parks for granting permits to collect plants and the administrations of the Jeseníky and Beskydy National Conservation Areas for cooperation. Samples from the core Central European area were collected in 2007–2011 in the Czech Republic and adjacent countries (Table S1; i.e. ‘Central European dataset’). In small and medium-sized populations (up to 30 individuals), all plants were sampled. In the case of three large populations (pop. no. 2, 13 and 22), a representative proportion of individuals equally covering the entire range of morphological variation was sampled. Because of high clonal ability of these taxa, we sampled 2–5 distant plants of each morphotype. In total, 561 individuals from 57 populations (mean 10 individuals per population, range 1–56) were subjected to flow cytometric estimation of genome size. A majority of these plants (well developed and undamaged individuals) were also subjected to morphometric analyses (466/313 individuals from 55/49 populations for numerical/geometric morphometrics of Central Europe dataset and 57/51 individuals from 30/29 populations from Northern Europe, respectively). Within the Czech Republic, almost all known recent populations were sampled. As comparative material, 22 additional populations/44 individuals were sampled in Scandinavia (Finland, Sweden, Norway) and 7 populations/19 individuals in Scotland and Wales for estimation of genome size (together hereafter referred to as the ‘Northern European dataset’). Both datasets were treated separately in subsequent analyses.

**Figure 1:**
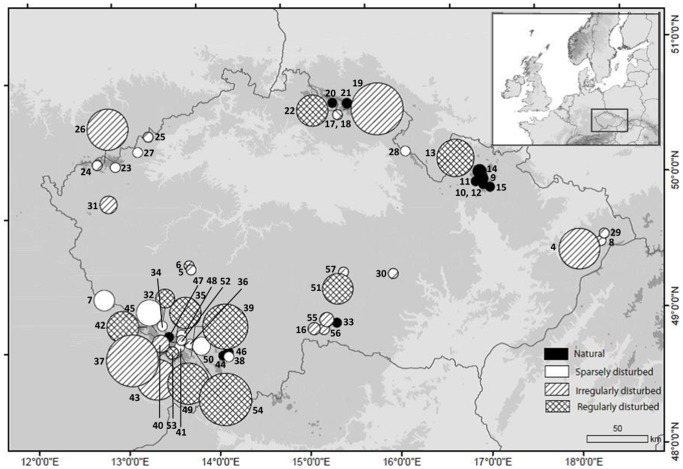
Sample localities of *Diphasiastrum* taxa in eastern Central Europe. The size of the symbols is proportional to variance in genome size of individuals within populations (i.e. roughly corresponding to the taxonomic complexity of populations); the color pattern reflects different habitats occupied by the populations.

Each population was localized using a GPS device (Garmin eTrex Legend; WGS 84). Rate of human disturbance was estimated and classified at each locality into four types: natural (sub/alpine zones, spruce forest etc.), sparsely disturbed (forest road margins), irregularly disturbed (timber storage sites) and regularly disturbed (ski slopes and other deforested strips; see also Figure 1). One well developed and intact sterile shoot was sampled per each individual. Fresh material was used for flow cytometric analyses (FCM) and numerical and geometric morphometric. Each accession was crosscheck-determined (independently confirmed by two team members) following several determination keys and floras [40], [66], [67]. The dimensions of ventral, lateral and dorsal leaves and their size in relation to the stem were used as the most important diagnostic characters [20], [66], [67]. During determination of specimens, we first identified indisputable morphotypes of basic species and then classified intermediate accessions. However, several individuals combined characters of all involved species, so their final determination must be treated as doubtful. Still, these doubtful individuals did not influence the results because even easily determinable basic species are represented by extremely high variation both in genome size and in morphology. This taxonomic determination served only to passively display taxa in ordination diagrams and was not used in any statistical analysis.

### Flow cytometry

Absolute genome sizes (C-values; [68]) were determined using a Cyflow SL instrument (Partec GmbH, Münster, Germany) equipped with a green solid-state laser (Cobolt Samba, 532 nm, 100 mW). For sample preparation, a slightly modified two-step procedure using Otto buffers was adopted [69]. *Pisum sativum* cv. *Ctirad* (2C = 9.09 pg; [70]) was used as the internal standard. Intact shoots together with an appropriate amount of standard tissue were chopped with a sharp razor blade in a Petri dish containing 500 µl of ice-cold Otto I buffer (0.1 M citric acid, 0.5% Tween 20; [71]). The suspension was filtered through a 42-µm nylon mesh and incubated for approx. 10 min at room temperature. Finally, the suspension was stained by a solution containing 1 ml of Otto II buffer (0.4 M Na_2_HPO_4_·12 H_2_O), β-mercaptoethanol (final concentration of 2 µl/ml), propidium iodide (PI) and RNase IIA (both at final concentrations of 50 µg/ml). Samples were stained for 5 min at room temperature and run through the flow cytometer. Isolated stained nuclei were excited with a laser beam, and the fluorescence intensity of 5,000 particles was recorded. Only histograms not exceeding a 6% coefficient of variance (CV) of G0/G1 peaks were analyzed further. The reliability of FCM measurements (i.e. between-plant differences) was repeatedly confirmed in simultaneous runs of *Diphasiastrum* accessions yielding distinct fluorescence intensities (i.e. resulting in furcate double peaks in FCM histograms; [72]). In order to cover a larger spatial scale, most of the samples (566, 91%) were measured at one time point only. Nevertheless, we checked for time stability of the measurements both over a short time period (three subsequent days, 52 samples) and over a long time period (once per month over three subsequent months, 6 samples). We did not count chromosomes because chromosome numbers of *Diphasiastrum* taxa were estimated many times with identical results (2n = 46; e.g. [14], [18], [73], [74]) and our interpretation of ploidy levels is in congruent with a previous flow cytometric study [20].

### Numerical morphometrics

In order to examine morphological variation of Central European *Diphasiastrum* (and of Northern Europe for comparison), 16 characters (Table 1) were measured. Well developed sterile shoots and fertile branchlets (if present) were directly used for morphometrics. The characters measured included traits used for the determination of taxa [12], [40], [66], [67], [75]–[78]. The characters measured were especially focused on the leaf proportions of fresh sterile shoots as follows: ventral leaf length (VL) and width in the widest part (VW); lateral leaf length (LL), width in the widest part (LW) and width between single leaf axillae (LD); dorsal leaf length (DL) and width in the widest part (DW). The position among different parts of ventral, lateral and dorsal leaves were measured in 9 characters: top of the lower ventral leaf to the top of the upper ventral leaf (VLU), top to base of the upper ventral leaf (VBU), top of the lower lateral leaf to the top of the upper lateral leaf (LLU), top to base of the upper lateral leaf (LBU), width between bases of lateral leaves (SW), top of the lower dorsal leaf to the top of the upper dorsal leaf (DLU), top to base of the upper dorsal leaf (DBU), width of the shoot at the widest point (DLW), width of the shoot – width of the lateral leaf (DWL). Height of the plant was not included due to its pronounced environmentally conditioned plasticity (e.g. extreme plasticity among individuals of *D. alpinum* from exposed vs. shady sites; Figure S1). Basic descriptive statistical parameters were computed for each of the characters using the UNIVARIATE procedure in SAS (ver. 9.1). The correlative relationship among the characters was investigated using Pearson's correlation and the non-parametric Spearman's rank coefficients to detect high correlations (>0.95) and avoid distortion of the multivariate analysis. A principal component analysis (PCA; [79]), based on a correlation matrix, was performed to reduce the multidimensional nature of the character space using Canoco for Windows 4.5 [80]. Genome size was passively projected into PCA diagrams using a local regression (loess) model. A redundancy analysis (RDA, [81] with a Monte Carlo permutation test (999 permutations)) was applied to test the association between morphological variation and genome size, using Canoco for Windows. Primary morphometrics and flow cytometric data are available as Table S2.

**Table 1:** List of morphometric characters used in distance-based morphometric analyses.

Character number		Character short	Character
v1		GS	genome size
v2	ventral leaves	VL	leaf length
v3		VLU	top of the lower leaf to top of the upper leaf
v4		VW	width in the widest part
v5		VBU	top to base of the upper leaf
v6	lateral leaves	LL	leaf length
v7		LLU	top of the lower leaf to top of the upper leaf
v8		LW	width in the widest part
v9		LBU	top to base of the upper leaf
v10		SW	width between bases
v11		LD	width between single leaf axillae
v12	dorsal leaves	DL	leaf length
v13		DLU	top of the lower leaf to top of the upper leaf
v14		DW	width in the widest part
v15		DBU	top to base of the upper leaf
v16		DLW	width of the shoot at the widest point
v17		DWL	width of the shoot - width of the lateral leaf

### Geometric morphometrics

**Figure 2:**
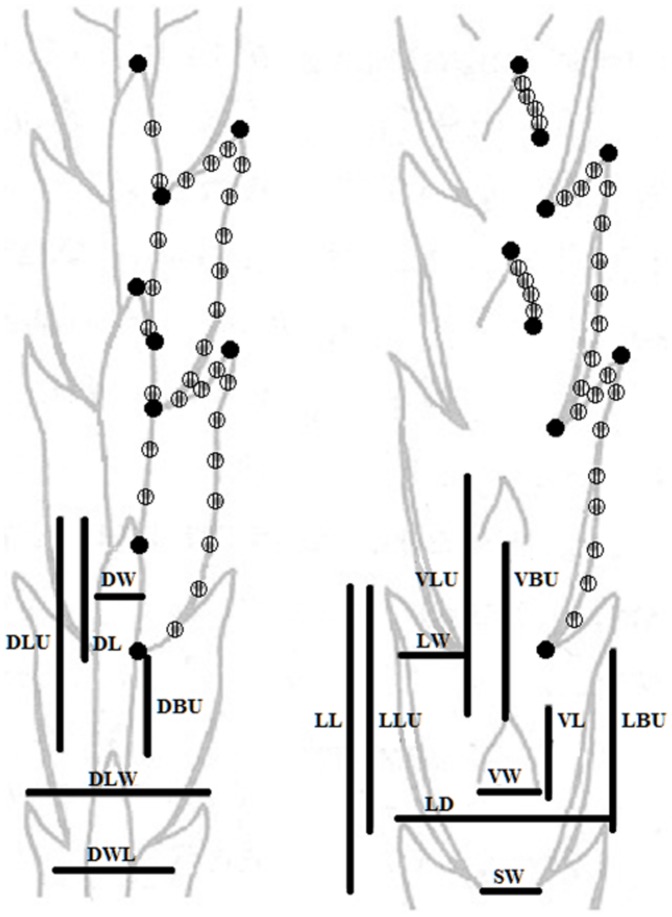
Characters used in morphometric analyses. Characters localized on the ventral and dorsal side of the stem of *Diphasiastrum* taxa. The lines indicate variables measured for numerical morphometrics; the points denote landmarks (fulfilled dot) and sliding semilandmarks (striped dot) used in geometric morphometry.

Photographs (RGB color images – JPG) of adult well developed parts of the stem were taken using an Olympus C-7070 digital camera mounted on an Olympus SZX12 binocular microscope) to investigate the variation in the shape and position of leaves using the thin plate spline method with sliding semilandmarks [82], [83]. The shape and leaf position of the dorsal and ventral part of the branch was assessed independently. For both the ventral and dorsal part of the stem, two adjacent nodes with corresponding leaves were chosen (9 landmarks/28 semilandmarks in the dorsal part and 9/28 in the ventral part, respectively; Figure 2). Due to branch symmetry, only one half of the structure was described by landmarks and analyzed. Landmarks were digitalized using tpsDig software [84]. Individual objects were superimposed by a generalized Procrustes analysis with sliding semilandmarks in tpsRelw ver. 1.49 [84]; for the scatter of superimposed landmarks). Then a relative warp analysis (RWA) was performed also using tpsRelw (α set to 0). The RWA scores were then visualized with the PCA procedure in Canoco for Windows, and genome size was projected using a local regression model. In order to assess the level and significance of covariation between the shape (represented as 34 shape coordinates) and genome size of the investigated plants, the two-block partial least squares (PLS) method [85] incorporated in tpsPls ver. 1.18 [86] was used. The PLS method reduces the dimensionality of the data by creating new linear combinations of variables (singular axes) that were calculated to maximize the covariation between two datasets [87], i.e. morphology and genome size in our case. A permutation test (999 permutations) was used to test whether the correlation along the singular axes was higher than would be expected by chance. This procedure allows extraction of a single axis of shape change that is most significantly correlated with changes in genome size [88]. In addition, the same method was also applied to assess the levels of covariation between the two geometric morphometric datasets (ventral and dorsal branch side).

### Spore abortion percentage

The spore abortion percentage (Ab) was estimated in order to confirm the spore fitness of individual taxa. Spores were collected from morphologically typical individuals with developed spores of each taxon (ripe strobili are found only rarely in the field). The spore abortion percentage was estimated by counting the number of aborted spores in a random sample of 100 spores per plant. Spores were considered aborted when they lacked a protoplast or were collapsed [89]. Spores were investigated under a light microscope (Olympus CH30) under 100× magnification.

## Results

**Figure 3:**
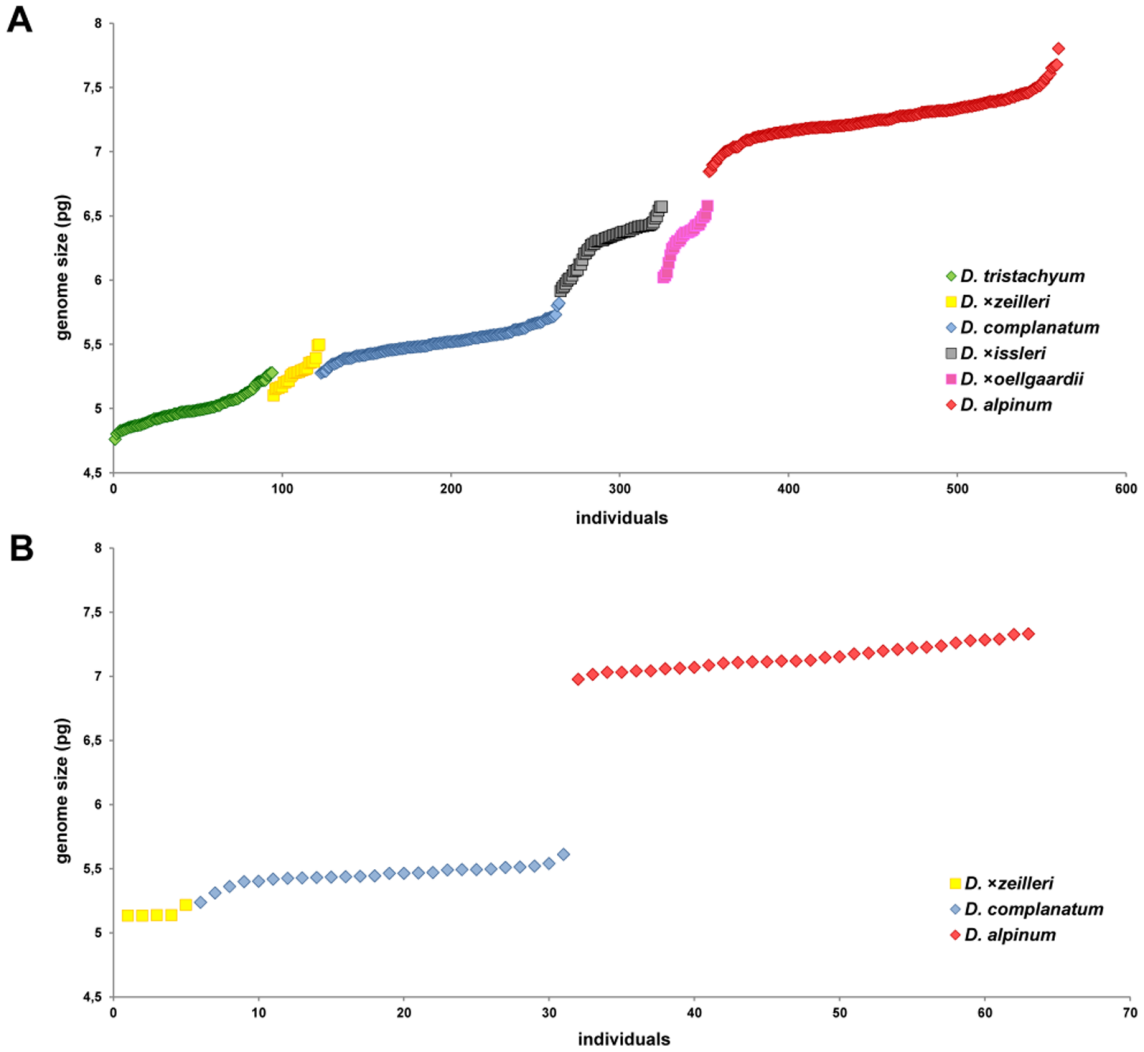
Distribution of absolute genome sizes of Diphasiastrum samples. Absolute genome sizes of *Diphasiastrum* individuals assigned to six European taxa in Central (A; 561 individuals, range 4.73–7.80 pg) and Northern (B; 63 individuals, range 5.13–7.33 pg) Europe. Different colors denote species as independently cross-check determined using several regional keys and floras (i.e. a passive illustrative projection; see Methods for details).

Coefficients of variance (CV) of all obtained flow cytometric histograms did not exceed 6% (range 1.22–5.78%, mean CV = 2.93%; S.D. = ±0.64). 2C-values of the Central European samples varied between 4.76 and 7.8 pg, mean 6.19, S.D = ±0.92. Accessions of pure (single species) populations of basic taxa varied in *D. alpinum* 6.43–7.68 pg and *D. complanatum* 5.24–5.72 pg (unfortunately *D. tristachyum* did not occur in pure populations). North European plants ranged from 5.13 to 7.33 pg, mean 6.29, S.D = ±0.89; plants with the smallest genome sizes assigned to *D. tristachyum* were absent within this region (Table 2). Fluorescence values of replicated measurements turned out to be highly stable for samples analyzed on three subsequent days (the maximum difference was 2% in 52 triplicates) and in three subsequent months (the maximum difference among analyses did not exceed 3% in any of the six sample triplicates) and thus met the standard criteria for reproducibility of FCM genome size measurements [69]. Importantly, absolute genome size of Central European individuals increased in a continuous fashion whereas North European plants split into three groups (two of them highly distinct; Figure 3). Absolute genome sizes of particular Central European basic species (cross-check determined) tended to differ (Figure 3), even though hybrids created a continuum of genome size values. The most intricate intervals of genome sizes were found in *D. ×issleri* and *D. ×oellgaardii*, which completely overlap. Their morphology overlaps too, see below. The situation in Scandinavia and the British Isles turned out to be less difficult compared to the Central European region. Intermediate taxa are less abundant there (*D. alpinum* and *D. complanatum* dominate in Norway, Sweden and Finland). Nevertheless, *D. ×zeilleri* was found frequently in Finland.

**Table 2:** Range of absolute genome sizes with its average and spore abortion percentage of hybrids and basic taxa of *Diphasiastrum* under study.

Taxon	No. of sampled individuals	Average 2C value (pg) ± S.D.	2C values range (pg)	Ab (%)/GS of measured indiv. (pg)
*D. tristachyum*	95	5.00±0.12	4.76–5.31	2/4.95; 8/5.09
*D. zeilleri* (CE)	28	5.27±0.10	5.10–5.50	0/5.21
*D. zeilleri* (NE)	6	5.16±0.05	5.13–5.24	
*D. complanatum* (CE)	142	5.51±0.11	5.28–5.82	1/5.48; 0/5.62
*D. complanatum* (NE)	25	5.46±0.06	5.31–5.61	
*D. issleri*	61	6.29±0.16	5.91–6.57	2/6.31; 7/6.38
*D. oellgaardii*	27	6.33±0.14	6.02–6.58	4/6.37; 2/6.46
*D. alpinum* (CE)	208	7.26±0.16	6.84–7.80	0/7.65
*D. alpinum* (NE)	32	7.15±0.10	6.98–7.33	

2C values - absolute genome sizes.

Ab - average and spore abortion percentage.

CE - Central European dataset.

NE - North European dataset.

We detected very low genome size variation in populations comprising a single taxon. These occur mostly in primary habitats and in areas with irregular or one-off disturbances, e.g. road margins and timber storage places. The highest variation in genome size was found in several populations that consisted of all six species and in mixed populations composed of *D. alpinum* and *D. tristachyum* (pop. 2, 19, 37, 13 and 22). Such populations occurred mostly at regularly disturbed sites, for example, ski slopes or other deforested strips (Figure 1 and Table S1).

**Figure 4:**
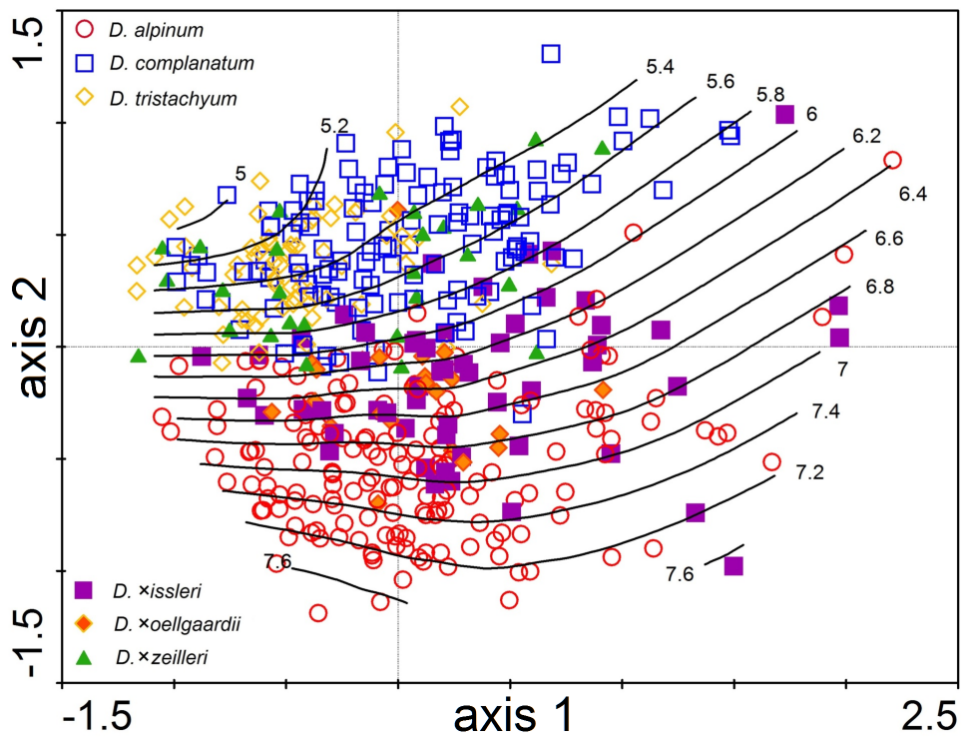
Principal component analysis of *Diphasiastrum* taxa. PCA of 466 individuals from Central Europe based on 16 vegetative morphological characters (the first and second ordination axis explain 33.4% and 27.2% of total variation, respectively). Genome size (values in pg DNA) is passively projected in the diagram using a local regression (loess) model. Individual accessions are designated by different symbols based on their independent taxonomic determination according to regional keys and floras (i.e. a passive illustrative projection).

**Figure 5:**
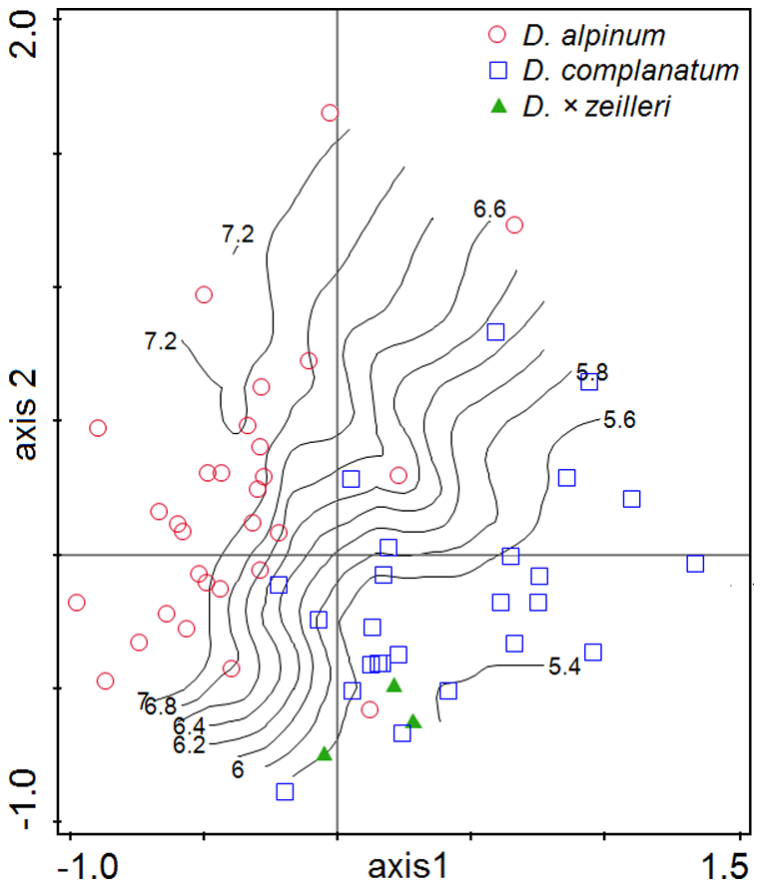
Principal component analysis of *Diphasiastrum* taxa from Northern Europe. PCA of 57 individuals based on 16 vegetative morphological characters (the first and second ordination axis explain 29.0% and 23.9% of total variation, respectively) illustrates different pattern of morphological variation in Northern Europe. Genome size (values in pg DNA) is passively projected in the diagram using a local regression (loess) model. Individual accessions are designated by different symbols based on their independent taxonomic determination according to regional keys and floras (i.e. a passive illustrative projection). Outlaying *D. alpinum* accession (0.408, 0.198) is an example of extremely shaded ecotype (see also Figure S1).

**Figure 6:**
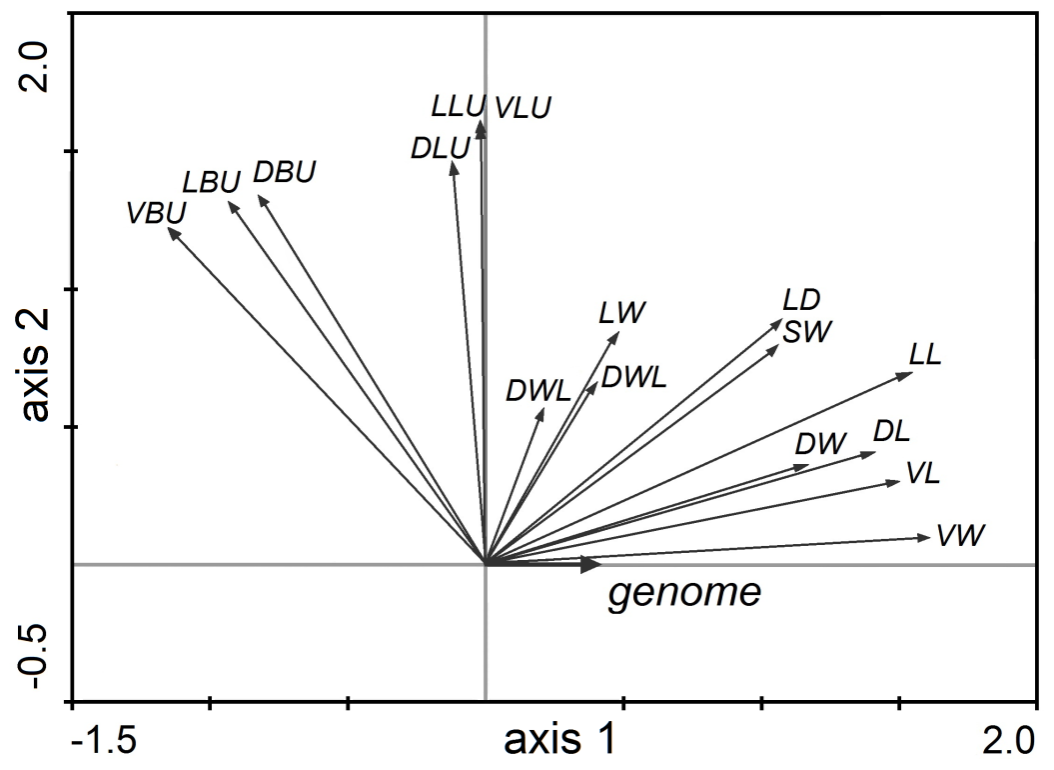
Redundancy analysis. RDA showing the change in values of 16 vegetative morphological characters measured on 466 accessions of *Diphasiastrum* taxa along a gradient of genome size (for an explanation of the codes, see Table 1; the canonical axis (axis 1) explains 18.4%, and the first unconstrained axis (axis 2) explains 33.0% of total variation, respectively).

No tightly correlated characters (i.e. with a correlation coefficient >0.95) were found in the correlation analysis (CORR), so all vegetative characters were included in the multivariate analyses. The PCA analysis (Figure 4) revealed a different morphological trends of accessions independently assigned to the three basic species; *D. complanatum* was partly separated along the first axis (which tends to be positively correlated with VBU, LBU, LLU and also with VLU, DLU, DBU), while *D. alpinum* was well separated along the second PCA axis (which is negatively correlated with VBU, LBU and DBU but positively correlated with VL, LL and DL). The third axis partly separated *D. tristachyum* (this axis tends to be positively correlated with DLW). On the contrary, individuals assigned to hybrids overlapped with basic species or were scattered among them (Figure 4). But still mind the trend illustrative character of displayed morphologically established groups (cross-check determined). Importantly, genome size appeared to be well correlated with the second PCA axis (see the perpendicularly oriented loess curves in Figure 4). Unlike pattern (probably influenced by different habitat and taxa composition) showed PCA analysis of Northern Europe dataset (Figure 5, Figure S4). A significant association between morphology and genome size was further confirmed by RDA (p = 0.001, 999 permutations). The morphological characters VW, VL, DL, DW and DL exhibited the strongest positive correlation with the canonical axis whereas the remaining characters were correlated only weakly or not at all (Figure 6).

**Figure 7:**
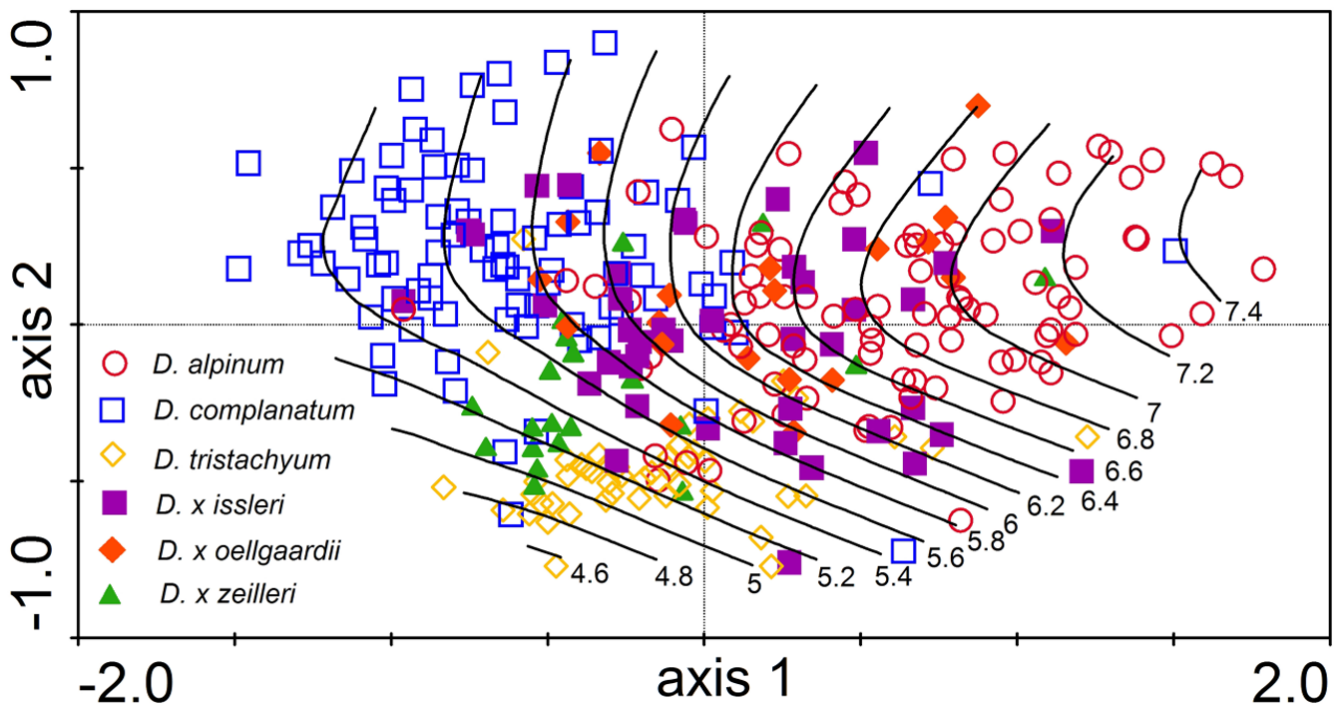
Variation in the shape of the ventral side of the stem. Relative warp analysis of 313 *Diphasiastrum* taxa accessions based on 37 landmarks (the first and second ordination axis explain 45.7% and 12.2% of total variation, respectively). Genome size (values in pg DNA) is passively projected in the diagram using a local regression (loess) model.

**Figure 8:**
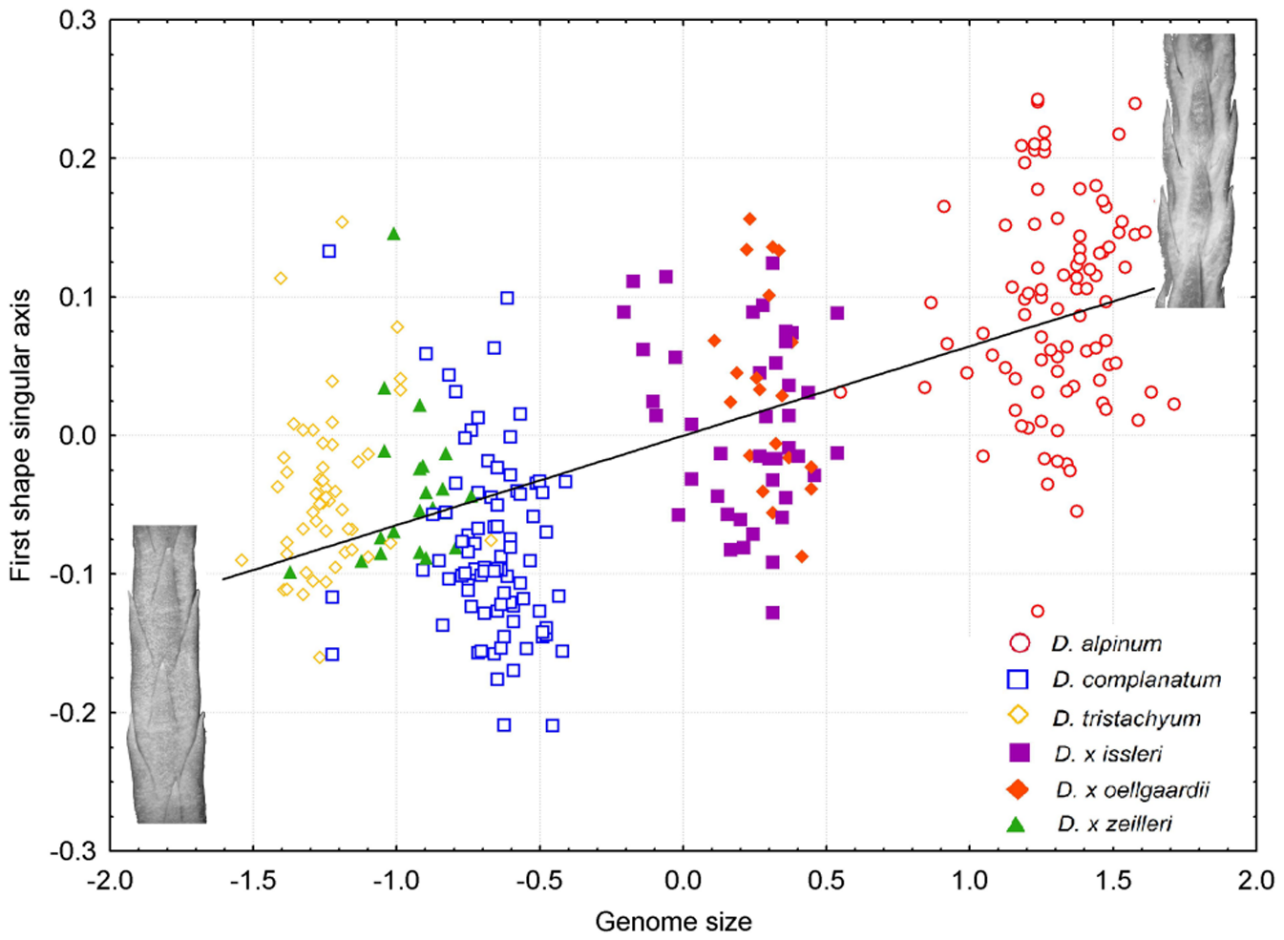
A partial least squares correlation between the shape and genome size. PLS analysis of *Diphasiastrum* taxa (correlation coefficient is 0.67) confirmed correlation between the shape the ventral side of the stem and genome size. A taxonomic determination based on regional keys and floras is passively projected using differently colored symbols. Individual specimens are shown to highlight the shape at the upper and lower genome size extremes.

**Figure 9:**
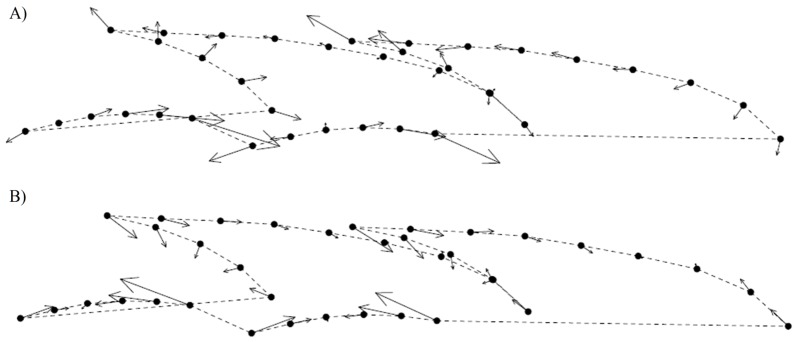
Warp diagram of shape change depending on genome size. Warp diagram showing shape change in relation to increasing (A)/decreasing (B) genome size. Points represent landmarks of the sample with mean position on the genome size singular axis, and arrows represent the vector of shape change between the largest (A) and smallest (B) genome size values.

Variation in the shape of the ventral and dorsal side of the stem, respectively, showed a very similar pattern (significant strong covariation among ventral and dorsal shape singular axes detected by PLS, r = 0.73, P<0.001), which strongly corresponded to the results of distance-based morphometrics. Again, the basic species were well-separated from each other (showing even better separation of *D. complanatum* and *D. tristachyum* than in distance-based morphometrics), accessions of hybrids form transitions among them (Figure 7 and Figure S3). The first singular axis of shape change and the first singular axis corresponding to genome size were significantly correlated for both the ventral and dorsal side of the *Diphasiastrum* stem (PLS, r = 0.67; P = 0.01 and r = 0.65; P = 0.01 for the ventral and dorsal side, respectively), and this covariation was in both cases significantly higher than expected by chance (Permutation test, P<0.001; Figure 8). A vector projection of the deviation from the mean reference (Figure 9) provides a visual demonstration of how the shape changes along the axis of maximum covariation [90]. Along the singular axis corresponding to genome size (from *D. tristachyum* to *D. alpinum*), distances between leaves become shorter, and leaves become smaller. Because the Procrustes superimposition procedure separates shape variation from size variation, the pattern of the correlation between the amount of nuclear DNA and centroid size of objects could be assessed independently. In both the ventral and the dorsal dataset, centroid size was significantly negatively correlated with genome size (p = 0.001 in both datasets); however, the correlation was very weak (r = 0.18 and 0.17 for the ventral and dorsal side, respectively).

All taxa including basic species and hybrids usually formed developed spores (Table 2). The proportion of aborted spores varied between 0 and 8% with no obvious differences among species.

## Discussion

Our study revealed a continuous pattern in both morphology and genome size among taxa of European *Diphasiastrum*. Importantly, variation in both distance-based morphological characters and in overall shape was strongly correlated with genome size. Although genome size might have a direct or indirect effects on various plant traits including morphology e.g. size of plant cells, seed or spore size, phenology [90]–[96], we interpret the correlation in *Diphasiastrum* rather as a consequence of taxonomical heterogeneity within the dataset. Firstly, the patterns in morphology and genome size clearly matched the independent cross-check determination of the taxa (i.e. genome size corresponded to the taxonomical assignment). Secondly, the rather complicated morphological differences detected (e.g. in the position and shape of ventral and lateral leaves) can hardly be explained merely by the nucleotypic effect of genome size on plant traits (e.g. [94], [95]). Finally, genome size has frequently been shown to be a neutral marker within closely related taxa complexes, discriminating individual taxa or clades rather than being a factor that directly influences traits of adaptive value (e.g. [61], [62], [64], [97]). Caution should be taken when interpreting small differences in genome size [98], [99]. We are nevertheless convinced that our results are not negatively influenced by methodological artefacts (e.g. influence of secondary metabolites, DNA degradation, instrumental shifts etc.; [69]). Firstly, low coefficients of variation were achieved which are incompatible with the presence of interfering secondary metabolites. Secondly, genome size values measured from the same 58 samples over longer time periods (52 samples measured three times in one week and 6 measured once monthly over three months) also remained stable. Finally, simultaneously analyzed plants with distinct genome size values resulted in distinct peaks (Figure S2), which is considered the most convincing piece of evidence for genuine differences in nuclear DNA content [68]. A possible strong influence of aneuploidy could also be ruled out based on results of morphometrics, where absolute genome size explains a major part of the observed variation. In addition, our genome size values correspond to the results of an independent flow cytometric survey of the same taxa of *Diphasiastrum* [20]. Possibility of samples with different ploidy level occurrence is highly improbable due to generally lower range of genome size values. Potential triploids were refused via confrontation of genome size values with morphology.

### Introgression mirrored by continuous genome size variation

The continuous rather than discrete pattern of variation detected both in morphology and genome size among Central European accessions suggests frequent introgressive hybridization among the basic *Diphasiastrum* species within this area. Although the genome sizes of individuals determined as basic species tend to be distinct (except for a slight overlap of these individuals with *D. tristachyum* and *D. complanatum*), genome sizes of individuals determined as hybrids (*D. ×issleri*, *D. ×oellgaardii* and *D. ×zeilleri*) create a continuum linking these distinct values. Gene flow among the basic species thus does not result in the formation of stable hybrid zones with sterile hybrids as is usually the case in ferns [10], [100], [101]. Instead, it seems that the populations investigated represent reoccurring hybrid zones with fertile hybrids probably forming hybrid swarms. The hypothesis of frequent backcrossing and consequent introgression in *Diphasiastrum* has already been suggested by molecular analyses (sequence data from three low-copy regions of the nuclear genome; [18]). It may also be supported by the complete fitness of hybrid spores (Table 2 and also [102]). Frequent gene flow among species is probably facilitated by intergametophytic mating, a prevalent phenomenon among homosporous lycopods [103].

Interestingly, such a continuum has not been found in other parts of Europe where the co-occurrence of the basic species is known to be rare. Markedly discrete genome size values were detected both in the Northern European dataset (i.e. Scandinavia and the British Isles; Figure 3) and in a previous flow cytometric survey in various parts of Europe [20]. This pattern together with the low frequency of the hybrid taxa in Northern and Western Europe [36], [37] suggest that the frequencies of backcrossing within these areas are generally low although Aagaard (2009) indicated backcrossing at six sites scattered in Western Europe. The low frequency of hybrids outside Central Europe might be caused by fewer suitable habitats, i.e. secondary human-disturbed sites (see discussion below). Tundra and taiga habitats suit *D. alpinum* and *D. complanatum*. *D. ×zeilleri* was repeatedly found in Finland (even without the basic species present at localities). This might explain the taxonomic confusion surrounding *D. tristachyum* and *D. ×zeilleri* in several floras of northeast Europe [33], [77], [104]. It should be noted, however, that a low number of samples (e.g. four from Northern Europe in [20] and 63 in our Northern European dataset vs. 561 from C Europe) or non-random sampling (i.e. selection of typical individuals) might have contributed to the underestimation of the introgression levels within these areas. More intensive screening outside Central Europe, particularly targeted at taxonomically intricate populations, is needed to evaluate the overall levels of gene flow within *Diphasiastrum*. At the same time to obtain accurate quantitative characteristics of introgressants (e.g. parental combination, direction of hybridization, backcrossing rate) a suitable molecular marker should be adopted.

### Origin of hybrid swarm populations

In Central Europe, morphological and genome size variation indicates that *Diphasiastrum* is distributed in a mosaic of (i) single species populations, (ii) simple mixed populations of typically two *Diphasiastrum* species and scattered hybrids, and (iii) highly complex populations (hybrid swarms) consisting of two or all three basic species and several hybrids (pops. No. 2, 13, 22, 39 and 54; Figure 1). Whereas the first two population types tend to comprise small numbers of individuals, populations of the third type are usually composed of numerous individuals (up to a hundred). At least two of the three basic species (*D. alpinum* and *D. complanatum*) form taxonomically pure stands with negligible intra-population variation in genome size, but hybrids were predominantly found at localities where they co-occurred with basic species, resulting in populations with higher variance in genome size (Figure 1). Importantly, populations composed only of hybrids were extremely rare. A mere four of these populations (18, 24, 47 and 48), which are obviously in decline because they consisted of not more than three individuals, were detected. This pattern of distribution indicates a polytopic and probably recent origin of the hybrid taxa. Each mixed population is likely a result of an independent hybridization event. This is also supported by distinct habitat preferences of pure vs. complex populations. While pure populations of basic species prefer open subalpine habitats (*D. alpinum*) or moderately disturbed open forest patches and forest margins (*D. complanatum*), morphologically and cytologically intricate populations tend to occur in man-made secondary habitats such as timber storage places and deforested strips. *D. tristachyum* is a special case because pure populations (in boreo-continental pine forest) are extremely rare (e.g. we haven't recorded any vital one in Central Europe) and *D. tristachyum* predominantly occurs in mixed populations.

The most complex hybrid swarms occur almost exclusively in artificial habitats such as ski slopes. Our field experience supports the connection between the high rate of hybridization (reflected by enormous morphological variation) and human-influenced habitats (followed by the highest genome size variance on; Figure 1, Table 2 and Table S1). It is thus possible that human-induced habitat changes in Central Europe have brought together previously ecologically isolated basic species and thus largely promoted their hybridization. Similar cases of human-induced changes that lead to secondary contact of previously separated species, promoting their hybridization, have been documented, for example, in *Prunus fruticosa* vs. *P. cerasus* [105], *Viola lutea* subsp. *sudetica* vs. *V. tricolor* [4], *Senecio hercynicus* vs. *S. ovatus* [48], *Arctium lappa* vs. *A. tomentosum* vs. *A. minus* [49].

In Central Europe, *Diphasiastrum* represents a complex, highly variable group of closely related taxa that is still undergoing evolution. The vast variation of hybrids that exist in nature may act as a “hybrid bridge” necessary for the introgression of genetic material between taxa with the potential for adaptive evolution [100]. Hybridization and introgression of single genes controlling traits with adaptive potential may cause reproductive isolation and, consequently, speciation [106]. Additional research, such as molecular study testing model populations (simplified hybrid combination of 2 basic taxa - *D. complanatum* vs. *D. alpinum* and their hybrids), is needed to shed some light on the evolutionary potential of novel genotypes generated by homoploid hybridization in *Diphasiastrum*.

### Taxonomical consequences

Our morphometric analyses confirm that absolute genome size correlates with morphological traits of particular groups of “taxa”. The most intricate pair of taxa turned out to be *D.* ×*issleri* and *D.* ×*oellgaardii*, which overlapped in all analyses (incl. the PLS analysis, which confirmed separation tendencies in other taxa groups; Figure 3 and Figure 8). Surprisingly, the absolute genome size interval of *D.* ×*oellgaardii* was shifted towards *D. alpinum* rather than being scattered around the mean value of its putative parental species. This shift may mirror more frequent backcrossing with *D. alpinum* or the participation of *D.* ×*issleri* in the hybridization (for a discussion of the possible occurrence of trihybrids, see [107]). The more complex origin of *D.* ×*oellgaardii* may be supported by the fact that *D.* ×*oellgaardii* (compared to other hybrids) never occurs without at least one of its parental taxa [22], [26]–[28], [108]. The position of *D. ×zeilleri* is less enigmatic because it does not overlap in its morphology and genome size with other putative hybrids. Moreover, its genome size is intermediate between its presumed parents although it strongly approaches that of *D. tristachyum*. Current taxonomic treatment doesn't fully reflects the real variation in European *Diphasiastrum* group. Even though characters from recent floras and keys were used in cross-check determination, several misplaced individuals were displayed in outputs of statistical analyses. Such pattern probably reflects enormous morphological plasticity of *Diphasiastrum* (see also Figure S1).

The presence of backcrossing and introgression in populations blurs the delimitation of taxa in Central Europe. Additional information yielded from morphometric analyses confirms the limited applicability of determination keys. Successful determination is restricted to particular regions where taxa do not frequently co-occur (e.g. Scandinavia, the British Isles and possibly parts of Western Europe). In Central Europe, the group is immensely intricate. In agreement with molecular investigations of [18], we reckon that only the so-called basic species should remain treated at the specific level and that all hybrid plants should be regarded as recent neohybrids occurring primarily in microevolutionarily active regions.

## Supporting Information

Figure S1 **Shape variation of** ***Diphasiastrum alpinum*** **- shade (upper) vs. exposed (down) ecotype.**
(TIF)

Figure S2 **Simultaneous analysis of PI-stained nuclei (absolute nuclear DNA content) isolated from fresh tissues of 4** ***Diphasiastrum*** **taxa.** A – *D. alpinum* (CV 2.06%), B – *D. complanatum* (CV 1.97%), C – *D. zeilleri* (CV 1.65%), D – *D. tristachyum* (CV 1.7%).
(TIF)

Figure S3 **Variation in the shape of the dorsal side of the stem of 313 accessions of** ***Diphasiastrum*** **taxa.** (relative warp analysis based on 37 landmarks; the first and second ordination axis explain 35.4% and 19.2% of total variation, respectively). Genome size (values in pg DNA) is passively projected into the diagram using a local regression (loess) model.
(TIF)

Figure S4 **Relative warp analysis of 49** ***Diphasiastrum*** **accessions from Northern Europe based on 37 landmarks.** Genome size (values in pg DNA) is passively projected in the diagram using a local regression (loess) model.
(TIF)

Table S1 **List of populations sampled.**
(DOC)

Table S2 **Primary data (flow cytometry, morphometrics).**
(PDF)
