# Supplementary figures and images for: Continuous Morphological Variation Correlated with Genome Size Indicates Frequent Introgressive Hybridization among Diphasiastrum Species (Lycopodiaceae) in Central Europe
Source: PLoS One. 2014 Jun 16;9(6):e99552. doi: 10.1371/journal.pone.0099552 (PMC4059668; doi:10.1371/journal.pone.0099552)

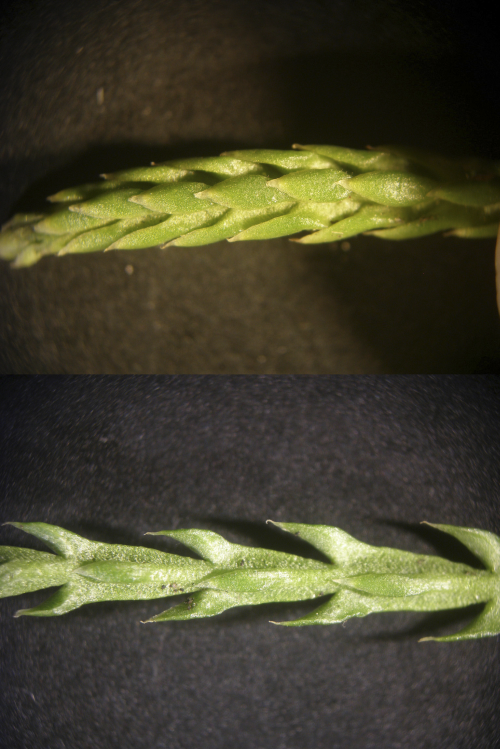

Supplement: Figure S1 — Shape variation of Diphasiastrum alpinum - shade (upper) vs. exposed (down) ecotype. (TIF) [file pone.0099552.s001.tif]

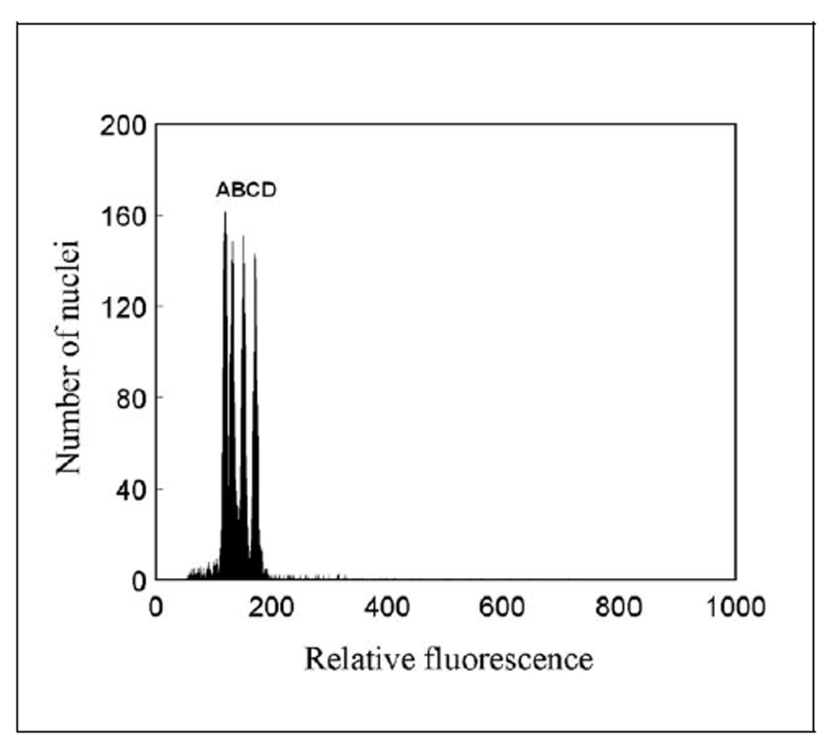

Supplement: Figure S2 — Simultaneous analysis of PI-stained nuclei (absolute nuclear DNA content) isolated from fresh tissues of 4 Diphasiastrum taxa. A – D. alpinum (CV 2.06%), B – D. complanatum (CV 1.97%), C – D. zeilleri (CV 1.65%), D – D. tristachyum (CV 1.7%). (TIF) [file pone.0099552.s002.tif]

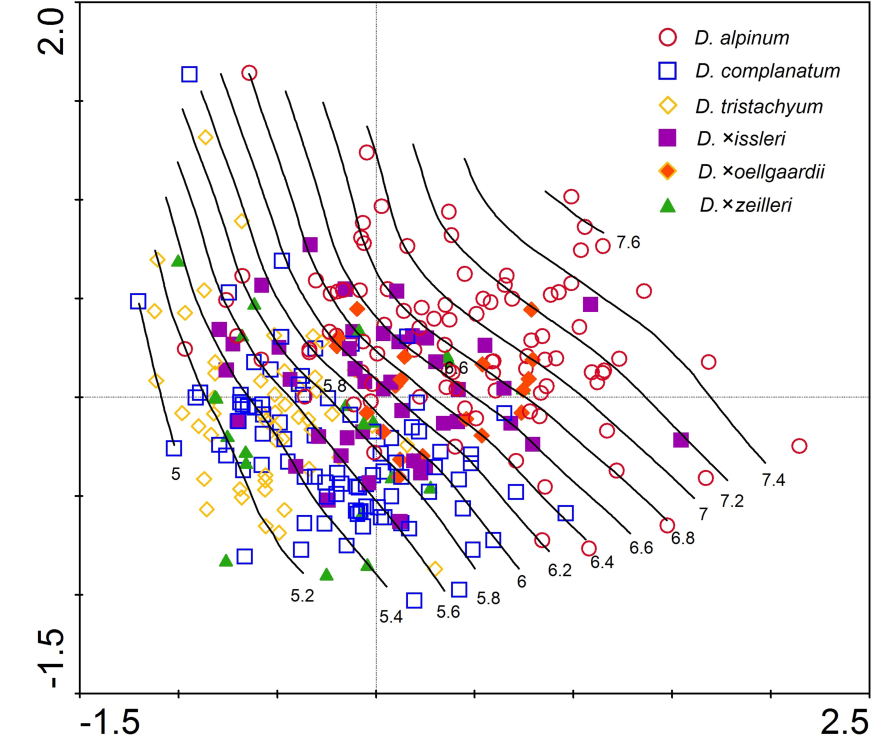

Supplement: Figure S3 — Variation in the shape of the dorsal side of the stem of 313 accessions of Diphasiastrum taxa. (relative warp analysis based on 37 landmarks; the first and second ordination axis explain 35.4% and 19.2% of total variation, respectively). Genome size (values in pg DNA) is passively projected into the diagram using a local regression (loess) model. (TIF) [file pone.0099552.s003.tif]

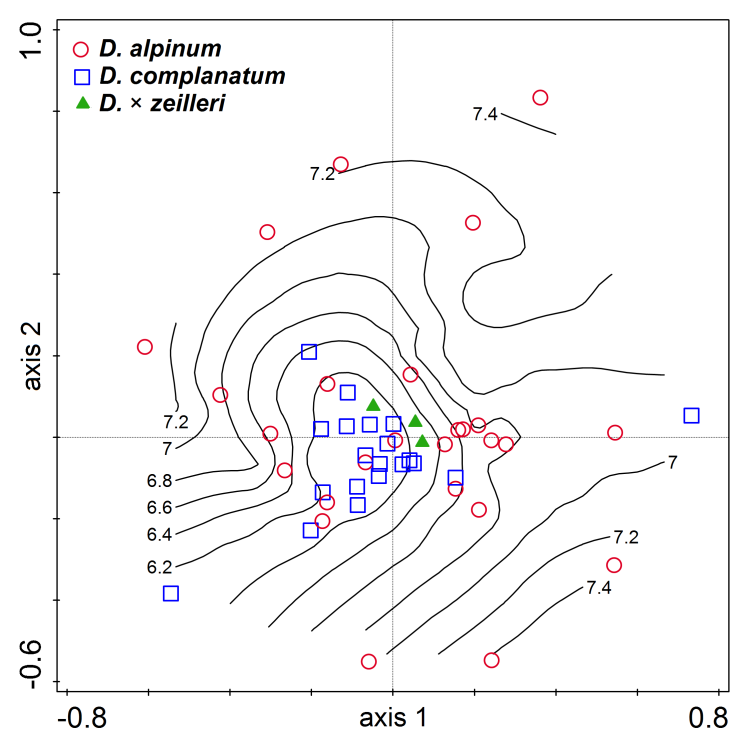

Supplement: Figure S4 — Relative warp analysis of 49 Diphasiastrum accessions from Northern Europe based on 37 landmarks. Genome size (values in pg DNA) is passively projected in the diagram using a local regression (loess) model. (TIF) [file pone.0099552.s004.tif]
