# Supplementary material for: Continuous Morphological Variation Correlated with Genome Size Indicates Frequent Introgressive Hybridization among Diphasiastrum Species (Lycopodiaceae) in Central Europe
Source: PLoS One. 2014 Jun 16;9(6):e99552. doi: 10.1371/journal.pone.0099552 (PMC4059668; doi:10.1371/journal.pone.0099552)
Supplement: Table S1 — List of populations sampled. (DOC) [file pone.0099552.s005.doc]

**Table S1:** List of populations sampled.

| **number** | **locality** | **longitude** | **latitude** | **date of collection** | **genome size (pg)** | **No of measured individuals** | **GS variation (%)*** |
| --- | --- | --- | --- | --- | --- | --- | --- |
| 1 | A, Alps, Oberatuern, alpine zone | 47°15´57.2" | 14°04´49.7" | 18.7.2011 | 7.54 | 1 | 0 |
| 2 | A, Hochficht, ski slope | 48°44´22.6" | 13°52´57.3" | 10.10.2007 | 4.82-7.46 | 57 | 1.09 |
| 3 | A, Kärnten, Oberkolbnitz, Reisseck-gruppe, alpine zone | 46°55´2.5" | 13°21´46.6" | 9.9.2009 | 7.10-7.20 | 3 | 0 |
| 4 | CZ, Beskydy Mts, Kladnatá hill, path margin | 49°29´42.2" | 18°12´3.3" | 12.11.2010 | 5.35-5.61, 7.22-7.37 | 7 | 0.73 |
| 5 | CZ, Brdy, Hutě pod Třemšínem, Kobylí hlava hill, spruce forest | 49°34´34" | 13°46´52" | 7.5.2010 | 5.05 | 1 | 0 |
| 6 | CZ, Brdy, Věšín, road margin | 49°36´28.5" | 13°45´42.8" | 29.6.2010 | 7.34-7.45 | 2 | 0 |
| 7 | CZ, Bohemian forest, Malinová hill, the area of former company | 49°23´24.3" | 12°46´17" | 8.7.2010 | 5.50-7.32 | 10 | 0.17 |
| 8 | CZ, Dolní Cerekev, Lysá hill, path margin, alpine zone | 49°32´28.9" | 18°27´48.5" | 12.11.2010 | 5.42-5.47 | 2 | 0 |
| 9 | CZ, Jeseníky Mts, Kamzičí hill, path margin, alpine zone | 50°06'15.4" | 17°14'38.9" | 26.9.2010 | 7.44-7.68 | 4 | 0.01 |
| 10 | CZ, Jeseníky Mts, Praděd, Peter´s rocks, alpine zone | 50°04'4.4" | 17°13'56" | 27.9.2010 | 7.36-7.65 | 5 | 0.01 |
| 11 | CZ, Jeseníky Mts, Praděd, Table´s rocks, alpine zone | 50°05'12.1" | 17°13'50.8" | 27.9.2010 | 7.51-7.66 | 2 | 0.01 |
| 12 | CZ, Jeseníky Mts, Praděd, Vysoká hole, alpine zone | 50°03'54.4" | 17°13'58.3" | 27.9.2010 | 7.49 | 2 | 0 |
| 13 | CZ, Jeseníky Mts, Ramzová-Černava, ski slope | 50°11´2.9" | 17°04´38.3" | 7.10.2009, 12.10.2011 | 4.95-7.44 | 58 | 0 |
| 14 | CZ, Jeseníky Mts, Snow basin, alpine zone | 50°08'40.4" | 17°08'21.9" | 25.9.2010 | 7.17-7.59 | 11 | 0.02 |
| 15 | CZ, Jeseníky Mts, Vysoká hole hill, alpine zone | 50°03'34.7" | 17°13'45.4" | 27.9.2010 | 7.25-7.52 | 6 | 0.01 |
| 16 | CZ, Kaproun, Červenka forest, spruce forest | 49°04´45" | 15°12´13" | 2.10.2009 | 4.85-5.48 | 8 | 0.06 |
| 17 | CZ, Krkonoše Mts, Herlíkovice, ski slope | 50°39´30.9" | 15°35´0.3" | 27.8.2009 | 5.41-5.58 | 7 | 0 |
| 18 | CZ, Krkonoše Mts, Herlíkovice, ski slope | 50°39´30.1" | 15°34´49.2" | 27.8.2009 | 6.12-6.31 | 2 | 0.01 |
| 19 | CZ, Krkonoše Mts, Husí challets, alpine zone | 50°41´8.2" | 15°39´30.9" | 27.8.2009 | 4.98, 7.17-7.18 | 3 | 1.07 |
| 20 | CZ, Krkonoše Mts, Kotelní jáma, alpine zone | 50°45´12.6" | 15°31´49.8" | 14.7.2011 | 7.02 | 1 | 0 |
| 21 | CZ, Krkonoše Mts, Luční challet, alpine zone | 50°44´10.9" | 15°42´16.4" | 29.6.2011 | 7.12 | 1 | 0 |
| 22 | CZ, Krkonoše Mts, Medvědín, ski slope | 50°44´11.3" | 15°36´13" | 27.8.2009, 28.6.2011 | 4.80-7.12 | 37 | 0.39 |
| 23 | CZ, Krušné Mts, Boží Dar, road margine | 50°24´21" | 12°46´13.3" | 2.7.2010 | 7.28-7.35 | 6 | 0 |
| 24 | CZ, Krušné Mts, Horní Blatná, moorland above the railway trail | 50°23´8.2" | 12°46´12" | 2.7.2010 | 6.30-6.42 | 3 | 0 |
| 25 | CZ, Krušné Mts, Kalek, overgrowing moorlands | 50°34´54.5" | 13°21´50" | 6.8.2011 | 5.39-5.43 | 2 | 0 |
| 26 | CZ, Krušné Mts, Ryžovna, overgrowing moorlands | 50°24´5.2" | 12°49´29.1" | 2.9.2011 | 5.42, 7.12-7.20 | 3 | 0.67 |
| 27 | CZ, Krušné Mts, Výsluní, path margine | 50°27´56,3" | 13°13´34.7" | 6.8.2011 | 5.32-5.43 | 3 | 0 |
| 28 | CZ, Orlické Mts, Sedloňov, Polom hill, former timber deposit | 50°20´55.4" | 16°20´0.4" | 26.7.2010 | 4.97-5.22 | 9 | 0.01 |
| 29 | CZ, Radhošťské Beskydy Mts, Krásná hill, path margin | 49°33´39.3" | 18°30´8.2" | 17.11.2010 | 5.34-5.70 | 5 | 0.01 |
| 30 | CZ, Sklené nad Oslavou, ditch along forest path | 49°26´50" | 16°04´17" | 25.10.2009 | 5.45-5.55 | 3 | 0 |
| 31 | CZ, Slavkovský les Mts, Bečov nad Teplou, path margin | 50°05´7.8" | 12°51´56.2" | 15.9.2010 | 5.36-6.42 | 13 | 0.12 |
| 32 | CZ, Šumava, Bučina, former forest clearing | 48°58´05.7" | 13°35´32.1" | 1.9.2010, 12.10.2011 | 6.43-7.32 | 16 | 0.06 |
| 33 | CZ, Šumava, Český Rudolec, opening of spruce-pine forest | 49°07´17.1" | 15°23´17.9" | 15.6.2010 | 5.47-5.69 | 6 | 0 |
| 34 | CZ, Šumava, Hůrka, former village | 49°07´28.6" | 13°19´34.3" | 1.9.2010 | 6.86-7.34 | 3 | 0.04 |
| 35 | CZ, Šumava, Churáňov, winter area for cross-country skiing | 49°04´04.2" | 13°37´09.7" | 1.7.2010, 12.10.2011 | 5.49-7.40 | 30 | 0.39 |
| 36 | CZ, Šumava, Klášterec, moorland above the railway trail | 49°02´04.4" | 13°43´29.2" | 1.7.2010, 12.10.2011 | 5.51-5.72 | 15 | 0 |
| 37 | CZ, Šumava, Knížecí pláně, moorland by the forest path | 48°58´08.4" | 13°38´51.2" | 1.7.2010 | 5.01-7.38 | 10 | 1.06 |
| 38 | CZ, Šumava, Ktiš, overgrowing moorland | 48°55´18.7" | 14°08´32.5" | 1.7.2010 | 5.24-5.39 | 3 | 0 |
| 39 | CZ, Šumava, Kubova huť, ski slope | 48°58´56.9" | 13°45´53.9" | 21.5.2010 | 4.89-7.27 | 19 | 0.68 |
| 40 | CZ, Šumava, Kvilda - Olšinka, margin of the former quarry | 49°02´05.4" | 13°35´59.8" | 1.9.2010 | 6.34-7.40 | 19 | 0.12 |
| 41 | CZ, Šumava, Kvilda, Tetřev, former borrow-pit for the sand | 49°00´28.1" | 13°33´24.9" | 1.9.2010, 12.10.2011 | 7.23-7.45 | 5 | 0.01 |
| 42 | CZ, Šumava, Laka lake, former forest clearing | 49°06´05.4" | 13°19´57.8" | 1.9.2010 | 6.36-7.28 | 6 | 0.14 |
| 43 | CZ, Šumava, Nové Hutě, slope above forest path between Nové Hutě and Přilba hill | 49°00´52.8" | 13°38´58.2" | 1.7.2010, 12.10.2011 | 5.44-7.27 | 13 | 0.66 |
| 44 | CZ, Šumava, Ostrá hora, overgrowing moorland | 48°56´04.6" | 14°05´18.7" | 1.7.2010 | 5.20-5.32 | 6 | 0 |
| 45 | CZ, Šumava, Vltava´s springs, former borrow-pit for the sand | 48°58´47.2" | 13°33´42.8" | 1.9.2010 | 5.91-7.37 | 8 | 0.27 |
| 46 | CZ, Šumava, Příslop, overgrowing moorland | 48°56´42" | 14°08´43.6" | 1.7.2010 | 5.46-5.59 | 5 | 0 |
| 47 | CZ, Šumava, Srní, spruce forest | 49°05´15.7" | 13°29´15" | 1.9.2010, 13.10.2011 | 6.01-6.12 | 3 | 0 |
| 48 | CZ, Šumava, Stachy, Nature reserve Pod Popelní horou hill, former pasture | 49°06´16" | 13°37´12" | 2.8.2011 | 6.40-6.49 | 2 | 0 |
| 49 | CZ, Šumava, Špičák hill, ski slope | 49°10´16.1" | 13°12´45.0" | 7.10.2009 | 5.07-7.46 | 27 | 0.4 |
| 50 | CZ, Šumava, Včelná pod Boubínem, former forest clearing | 49°00´36.1" | 13°50´53.7" | 1.7.2010, 12.10.2011 | 5.54-6.40 | 6 | 0.13 |
| 51 | CZ, Šumava, Vysočina - Čeřínek, ski slope | 49°22´25.2" | 15°25´53.2" | 6.9.2010 | 5.20-6.93 | 5 | 0.37 |
| 52 | CZ, Šumava, Zadov, above ski slopes | 49°03´39.1" | 13°37´33.3" | 7.10.2009, 12.10.2011 | 7.20-7.50 | 7 | 0.01 |
| 53 | CZ, Šumava, Zadov, around former ski jump | 49°03´39.6" | 13°37´32.5" | 1.7.2010 | 7.12-7.25 | 8 | 0 |
| 54 | CZ, Šumava, Žlíbský vrch, path margin | 48°56´10.9" | 13°43´51.8" | 21.5.2010 | 5.13-7.80 | 18 | 0.81 |
| 55 | CZ, Terezín, Červenka forest, spruce forest | 49°04´38" | 15°13´50" | 2.10.2009 | 4.73-5.56 | 30 | 0.08 |
| 56 | CZ, Terezín, Červenka forest, spruce forest | 49°04´45" | 15°14´14" | 2.10.2009 | 5.00-5.73 | 10 | 0.05 |
| 57 | CZ, Větrný Jeníkov, ditch along road | 49°29´14" | 15°30´25" | 25.10.2009 | 5.35-5.55 | 5 | 0 |
| 58 | F, Störsvik, pine forest | 60°05´10" | 24°18´09.1" | 8.7.2011 | 5.43-5.44 | 2 | 0 |
| 59 | F, Uusimaa, Nurmijärvi, pine forest | 60°26´49.4" | 23°34´0.9" | 7.7.2011 | 5.44-5.54 | 3 | 0 |
| 60 | F, Uusimaa, Nurmijärvi, Kiljava, pine forest | 60°30´44.6" | 24°42´5.2" | 3.7.2011 | 5.40-5.46 | 2 | 0 |
| 61 | F, Uusimaa, Nurmijärvi, Kiljava, pine forest | 60°29´34.1" | 24°39´25.1" | 7.7.2011 | 5.22-5.42 | 2 | 0.01 |
| 62 | F, Uusimaa, Tammisaari, pine forest | 60°01´0.8" | 23°34´1.9" | 2.7.2011 | 5.13-5.24 | 3 | 0 |
| 63 | F, Varsinais-Suomi, Karis - Lohja, pine forest | 60°06´14" | 23°46´30.3" | 8.7.2011 | 5.14-5.47 | 2 | 0.03 |
| 64 | F, Varsinais-Suomi, Karis - Lohja, pine forest | 60°06´50.4" | 23°48´03.4" | 8.7.2011 | 5.31-5.40 | 2 | 0 |
| 65 | F, Varsinais-Suomi, Kiikala, Tarvakas, forest clearing | 60°29´25.1" | 23°42´21" | 3.7.2011 | 5.36 | 1 | 0 |
| 66 | GB, Scotland, moorland | 57°41´55.6" | W4°66´22.1" | 26.8.2011 | 7.15-7.28 | 3 | 0 |
| 67 | GB, Scotland, moorland | 58°19´23.1" | W4°02´54.4" | 26.8.2011 | 7.15-7.18 | 2 | 0 |
| 68 | GB, Scotland, moorland | 53°00´28.2" | W4°08´43.3" | 28.8.2011 | 7.03-7.33 | 6 | 0.01 |
| 69 | GB, Scotland, moorland | 53°09´11" | W3°59´45.6" | 27.8.2011 | 7.04-7.28 | 4 | 0.01 |
| 70 | GB, Scotland, Knockhan Crag, cliffs | 58°02´13.6" | W5°03´49.2" | 16.10.2011 | 7.24 | 1 | 0 |
| 71 | N, Alnesvatnet, near to lake, alpine zone | 62°25´57.7" | 7°39´29" | 26.6.2009 | 7.10 | 1 | 0 |
| 72 | N, Dalsvelen, near to road to Røros, alpine zone | 62°32´56.6" | 11°26´08" | 26.6.2009 | 5.43 | 1 | 0 |
| 73 | N, Järvsö, ditch on the verge of the road | 61°39´01.9" | 16°10´56.4" | 11.8.2011 | 5.46-5.67 | 4 | 0.01 |
| 74 | N, Kalkovo lake, near to the railway line | 61°13´39.4" | 10°37´25.3" | 30.6.2009 | 7.04 | 1 | 0 |
| 75 | N, Linneset, lake Rengen shore | 64°03´40.4" | 14°03´15.9" | 28.6.2009 | 5.42-5.52 | 9 | 0 |
| 76 | N, between Formofoss and Sandmoen, alpine zone | 64°29´16.7" | 13°04´26.5" | 28.6.2009 | 7.08 | 1 | 0 |
| 77 | N, between Breidsjøen and Fofervatnet, moorland near to road | 61°29´10.1" | 9°29´17.3" | 30.6.2009 | 6.98 | 1 | 0 |
| 78 | N, between Dovre and Folldal, alpine zone | 62°01´48.7" | 9°24´49.8" | 26.6.2009 | 7.03 | 1 | 0 |
| 79 | N, between Lesja and Skogbygde, alpine zone | 62°02´18" | 8°58´03.6" | 28.6.2009 | 7.01 | 1 | 0 |
| 80 | N, NP Jotunheimen, alpine zone | 61°30'32.5'' | 8°48'05.5'' | 13.8.2011 | 7.12-7.22 | 3 | 0 |
| 81 | N, near NP Rondane, alpine zone | 61°56´17.2" | 10°00´29.3" | 6.9.2008 | 7.12-7.23 | 3 | 0 |
| 82 | N, Torsefeatn, alpine zone | 60°20´28.4" | 8°29´78.9" | 26.6.2009 | 7.07 | 1 | 0 |
| 83 | N, Trollhsimon, alpine zone | 62°43´33.9" | 9°10´11" | 11.8.2011 | 7.11-7.29 | 2 | 0.01 |
| 84 | N, Vik, alpine zone | 60°56´19.2" | 6°26´34.6" | 26.6.2009 | 7.06 | 1 | 0 |

* variation of the sum of individuals genome size
