# Supplementary material for: Continuous Morphological Variation Correlated with Genome Size Indicates Frequent Introgressive Hybridization among Diphasiastrum Species (Lycopodiaceae) in Central Europe
Source: PLoS One. 2014 Jun 16;9(6):e99552. doi: 10.1371/journal.pone.0099552 (PMC4059668; doi:10.1371/journal.pone.0099552)
Supplement: Table S2 — Primary data (flow cytometry, morphometrics). (PDF) [file pone.0099552.s006.pdf]

Supporting Infromation Table S2: Primary data

Flow cytometry

locality	ID	date	index	1/index	2C	CV sample	CV std
CZ, Beskydy Mts, Kladnatá hill	BES 01	16.11.2010	1,235	0,810	7,36	2,40	2,04
	BES 02	16.11.2010	1,234	0,810	7,37	2,55	2,03
	BES 03	16.11.2010	1,619	0,618	5,61	2,71	1,96
	BES 04	16.11.2010	1,210	0,826	7,51	2,82	2,54
	BES 05	16.11.2010	1,239	0,807	7,34	2,26	2,10
	BES 08	16.11.2010	1,698	0,589	5,35	2,82	1,97
	BES 09	16.11.2010	1,259	0,794	7,22	2,44	2,08
CZ, Brdy, Hutě pod Třemšínem, Kobylí hlava hill	TRI+P	13.7.2010	1,801	0,555	5,05	3,50	2,56
CZ, Brdy, Věšín	1+P	11.5.2010	1,220	0,820	7,45	4,84	2,57
	2+P	11.5.2010	1,238	0,808	7,34	4,48	2,10
CZ, Bohemian forest, Malinová hill	Malin.H.1+P	14.7.2010	1,415	0,707	6,42	3,16	2,49
	Malin.H.2	14.7.2010	1,654	0,605	5,50	3,84	2,40
	Malin.H.3	14.7.2010	1,396	0,716	6,51	2,79	2,49
	Malin.H.4	14.7.2010	1,414	0,707	6,43	2,84	2,56
	Malin.H.5	14.7.2010	1,241	0,806	7,32	2,85	2,05
	Malin.H.6	16.7.2010	1,400	0,714	6,49	2,58	2,53
	Malin.H.7	16.7.2010	1,426	0,701	6,37	3,35	2,84
	Malin.H.8	16.7.2010	1,408	0,710	6,46	2,76	2,55
	Malin.H.9	16.7.2010	1,430	0,699	6,36	4,27	2,87
	Malin.H.10	16.7.2010	1,423	0,703	6,39	3,81	2,62
CZ, Dolní Cerekev, Lysá hill	BES 06	16.11.2010	1,676	0,597	5,42	2,64	1,84
	BES 07	16.11.2010	1,663	0,601	5,47	2,67	2,51
CZ, Jeseníky Mts, Kamzičí hill	Je 1/1 +P	27.9.2010	1,184	0,845	7,68	3,32	1,87
	Je 1/2	27.9.2010	1,195	0,837	7,61	3,18	2,43
	Je 1/3	27.9.2010	1,185	0,844	7,67	3,35	2,11
	Je 1/4	27.9.2010	1,222	0,818	7,44	1,93	2,02
CZ, Jeseníky Mts, Praděd, Peter´s rocks	Je 3/1	27.9.2010	1,188	0,842	7,65	3,29	2,35
	Je 3/2	27.9.2010	1,228	0,814	7,40	2,37	2,25
	Je 3/3	27.9.2010	1,201	0,833	7,57	2,47	2,43
	Je 3/4	27.9.2010	1,233	0,811	7,37	3,31	2,64
	Je 3/5	27.9.2010	1,235	0,810	7,36	2,79	2,40
CZ, Jeseníky Mts, Praděd, Table´s rocks	Je 2/1	27.9.2010	1,187	0,842	7,66	3,33	2,12
	Je 2/2	27.9.2010	1,210	0,826	7,51	3,02	1,99
CZ, Jeseníky Mts, Praděd, Vysoká hole hill	Je 4/1	27.9.2010	1,214	0,824	7,49	2,57	2,03
	Je 4/2	27.9.2010	1,213	0,824	7,49	2,49	2,05
	RAMZ 01	4.11.2010	1,438	0,695	6,32	2,43	2,35
	RAMZ 02	4.11.2010	1,794	0,557	5,07	2,41	2,39
	RAMZ 03	4.11.2010	1,645	0,608	5,53	2,88	2,56
	RAMZ 04	4.11.2010	1,221	0,819	7,44	2,09	2,53
	RAMZ 05	4.11.2010	1,627	0,615	5,59	3,96	2,14
	RAMZ 06	4.11.2010	1,662	0,602	5,47	2,81	2,89
	RAMZ 07	4.11.2010	1,594	0,627	5,70	3,72	2,54
	RAMZ 08	4.11.2010	1,827	0,547	4,98	3,19	2,47
	RAMZ 09	4.11.2010	1,714	0,583	5,30	3,33	2,52
	RAMZ 10	4.11.2010	1,417	0,706	6,41	3,14	2,58

CZ, Jeseníky Mts, Ramzová-Černava ski slope	RAMZ 11	4.11.2010	1,419	0,705	6,41	2,34	2,30
	RAMZ 12	4.11.2010	1,447	0,691	6,28	2,66	2,22
	RAMZ 13	4.11.2010	1,826	0,548	4,98	2,94	2,65
	R1+P	23.10.2009	1,651	0,606	5,51	2,64	1,84
	R2+P	23.10.2009	1,718	0,582	5,29	2,83	2,43
	R3+P	23.10.2009	1,658	0,603	5,48	2,95	2,15
	R4+P	23.10.2009	1,692	0,591	5,37	2,74	1,89
	R5+P	23.10.2009	1,833	0,546	4,96	2,79	2,06
	R6+P	23.10.2009	1,741	0,574	5,22	2,61	1,96
	R7+P	23.10.2009	1,686	0,593	5,39	2,47	1,51
	R8+P	23.10.2009	1,617	0,618	5,62	2,89	2,17
	R9+P	23.10.2009	1,249	0,801	7,28	2,81	2,27
	R10+P	23.10.2009	1,419	0,705	6,41	2,76	2,39
	R11+P	23.10.2009	1,650	0,606	5,51	2,73	2,16
	R12+P	23.10.2009	1,663	0,601	5,47	2,64	1,93
	R13+P	23.10.2009	1,742	0,574	5,22	2,78	2,31
	R14+P	23.10.2009	1,744	0,573	5,21	2,80	2,49
	R15+P	23.10.2009	1,647	0,607	5,52	2,70	2,58
	R16+P	23.10.2009	1,249	0,801	7,28	2,81	2,27
	R17+P	23.10.2009	1,655	0,604	5,49	3,51	2,71
	R18+P	23.10.2009	1,782	0,561	5,10	2,73	2,61
	R19+P	23.10.2009	1,426	0,701	6,37	2,91	2,08
	R20+P	23.10.2009	1,675	0,597	5,43	2,81	2,26
	R21+P	23.10.2009	1,838	0,544	4,95	2,82	2,06
	R22+P	23.10.2009	1,264	0,791	7,19	2,88	2,34
	R23+P	23.10.2009	1,465	0,683	6,20	2,90	2,22
	R24+P	23.10.2009	1,693	0,591	5,37	2,39	2,40
	R25+P	23.10.2009	1,272	0,786	7,15	2,25	1,83
	R26+P	23.10.2009	1,243	0,805	7,31	2,35	2,49
	R27+P	23.10.2009	1,782	0,561	5,10	2,73	2,61
	R28+P	23.10.2009	1,596	0,627	5,70	3,95	2,75
	R29+P	23.10.2009	1,642	0,609	5,54	2,80	1,94
	R30+P	23.10.2009	1,643	0,609	5,53	2,86	2,13
	R31+P	23.10.2009	1,256	0,796	7,24	2,67	2,50
	R32+P	23.10.2009	1,680	0,595	5,41	2,68	2,34
	R33+P	23.10.2009	1,671	0,598	5,44	2,85	2,35
	R34+P	23.10.2009	1,685	0,593	5,39	3,52	2,57
	R35+P	23.10.2009	1,442	0,693	6,30	2,78	2,58
	R36+P	23.10.2009	1,672	0,598	5,44	2,78	2,44
	R37+P	23.10.2009	1,819	0,550	5,00	2,54	2,45
	R38+P	26.10.2009	1,720	0,581	5,28	2,70	2,34
	R39+P	26.10.2009	1,414	0,707	6,43	2,48	1,79
	R40+P	26.10.2009	1,629	0,614	5,58	2,59	1,76
	R41+P	26.10.2009	1,230	0,813	7,39	2,39	1,91
	R42+P	26.10.2009	1,814	0,551	5,01	2,59	2,20
	R43+P	26.10.2009	1,695	0,590	5,36	2,99	1,99
	R44+P	26.10.2009	1,613	0,620	5,64	2,74	1,92
	R45+P	26.10.2009	1,727	0,579	5,26	2,71	1,85
	Je 6/1	27.9.2010	1,216	0,822	7,48	3,88	1,79
	Je 6/1a	27.9.2010	1,267	0,789	7,17	2,77	2,78

CZ, Jeseníky Mts, Snow basin	Je 6/2	27.9.2010	1,232	0,812	7,38	2,27	2,48
	Je 6/3	27.9.2010	1,219	0,820	7,46	3,18	2,54
	Je 6/4	27.9.2010	1,260	0,794	7,21	3,07	2,64
	Je 6/5	27.9.2010	1,246	0,803	7,30	3,27	2,71
	Je 6/6	27.9.2010	1,224	0,817	7,43	2,24	2,39
	Je 6/7	27.9.2010	1,228	0,814	7,40	3,67	2,95
	Je 6/8	27.9.2010	1,217	0,822	7,47	3,46	2,91
	Je 6/9	27.9.2010	1,197	0,835	7,59	2,76	2,71
	Je 6/10	27.9.2010	1,200	0,833	7,58	3,71	2,42
CZ, Jeseníky Mts, Vysoká hole hill	Je 5/1	27.9.2010	1,253	0,798	7,25	2,60	2,45
	Je 5/2	27.9.2010	1,227	0,815	7,41	3,17	2,75
	Je 5/3	27.9.2010	1,208	0,828	7,52	4,11	3,16
	Je 5/3a	27.9.2010	1,224	0,817	7,43	3,94	2,93
	Je 5/4	27.9.2010	1,230	0,813	7,39	3,62	2,57
	Je 5/6	27.9.2010	1,238	0,808	7,34	3,99	3,26
CZ, Kaproun, Červenka forest	K1+P	10.12.2009	1,871	0,534	4,86	2,71	2,14
	K2+P	10.12.2009	1,700	0,588	5,35	2,92	2,39
	K3+P	10.12.2009	1,876	0,533	4,85	3,73	2,04
	K4+P	10.12.2009	1,842	0,543	4,93	3,05	1,75
	K5+P	10.12.2009	1,660	0,602	5,48	2,65	1,62
	K6+P	10.12.2009	1,873	0,534	4,85	2,86	2,38
	K7+P	10.12.2009	1,845	0,542	4,93	2,71	3,29
	K8+P	10.12.2009	1,820	0,549	4,99	3,22	1,72
CZ, Krkonoše Mts, Herlíkovice ski slope	KR2-1	9.9.2009	1,680	0,595	5,41	2,80	2,44
	KR2-2	8.9.2009	1,656	0,604	5,49	2,74	2,49
	KR2A	8.9.2009	1,679	0,596	5,41	2,55	2,03
	KR2B-1	8.9.2009	1,647	0,607	5,52	2,47	2,54
	KR2B-2	8.9.2009	1,647	0,607	5,52	2,96	2,27
	KR2B-3	8.9.2009	1,629	0,614	5,58	2,39	2,09
	KR2-3	8.9.2009	1,660	0,602	5,48	2,63	2,64
CZ, Krkonoše Mts, Herlíkovice ski slope	KR3-1	8.9.2009	1,441	0,694	6,31	3,10	2,26
	KR3-2	8.9.2009	1,485	0,673	6,12	2,62	2,02
CZ, Krkonoše Mts, Husí challets	KR4-1	8.9.2009	1,825	0,548	4,98	2,70	2,01
	KR4-2	7.9.2009	1,266	0,790	7,18	2,59	2,15
	KR4-3	7.9.2009	1,268	0,789	7,17	2,26	1,83
CZ, Krkonoše Mts, Kotelní jáma	KRK3	6.9.2011	1,284	0,779	7,08	3,06	3,31
CZ, Krkonoše Mts, Luční challet	KRK4	6.9.2011	1,276	0,784	7,12	3,04	2,80
	KR1-1	7.9.2009	1,846	0,542	4,92	2,84	2,00
	KR1-2	7.9.2009	1,680	0,595	5,41	2,90	2,68
	KR1-3	7.9.2009	1,516	0,660	6,00	2,92	2,32
	KR1-4	7.9.2009	1,722	0,581	5,28	2,90	3,64
	KR1-5	7.9.2009	1,893	0,528	4,80	2,88	1,96
	KR1-6	7.9.2009	1,442	0,693	6,30	2,37	1,63
	KR1-7	7.9.2009	1,537	0,651	5,91	2,50	2,80
	KR1-8	7.9.2009	1,719	0,582	5,29	2,67	2,32
	KR1-9	8.9.2009	1,456	0,687	6,24	2,57	1,72
	KR1-10	8.9.2009	1,562	0,640	5,82	3,28	3,85
	KR1-11	8.9.2009	1,855	0,539	4,90	2,89	2,50
	KR1-12	8.9.2009	1,868	0,535	4,87	2,82	1,86

CZ, Krkonoše Mts, Medvědín ski slope	KR1-13	8.9.2009	1,723	0,580	5,28	2,76	2,85
	KR1-14	8.9.2009	1,851	0,540	4,91	3,53	2,23
	KR1-15	8.9.2009	1,506	0,664	6,04	2,75	2,49
	KR1-16	8.9.2009	1,866	0,536	4,87	2,63	1,84
	KR1-17	7.9.2009	1,522	0,657	5,97	2,39	2,51
	KR1-18	7.9.2009	1,290	0,775	7,05	2,15	2,11
	KR1-19	7.9.2009	1,880	0,532	4,84	3,52	2,68
	KR1-20	7.9.2009	1,860	0,538	4,89	3,39	2,43
	KR1-21	9.9.2009	1,276	0,784	7,12	2,37	1,64
	KR1-22	9.9.2009	1,476	0,678	6,16	1,67	2,61
	KR1-23	8.9.2009	1,527	0,655	5,95	2,78	2,00
	KR1-24	8.9.2009	1,497	0,668	6,07	2,68	1,88
	KR1-25	8.9.2009	1,800	0,556	5,05	2,23	1,56
	KR1-26	8.9.2009	1,453	0,688	6,26	2,80	2,44
	KR1-27	8.9.2009	1,512	0,661	6,01	3,32	3,09
	KR1-28	8.9.2009	1,869	0,535	4,86	3,15	2,45
	KR1-29	8.9.2009	1,651	0,606	5,51	2,91	2,12
	KR1-30	8.9.2009	1,816	0,551	5,01	3,30	2,52
	KR1-31	8.9.2009	1,645	0,608	5,53	2,25	2,06
	KR1-32	8.9.2009	1,482	0,675	6,13	2,86	2,66
	KR1-33	8.9.2009	1,500	0,667	6,06	2,57	1,56
	KR1-34	9.9.2009	1,506	0,664	6,04	2,71	2,26
	KRK1 -1	6.9.2011	1,562	0,640	5,82	3,58	3,18
		2.11.2011	1,490	0,671	6,10	2,63	2,67
		3.11.2011	1,540	0,649	5,90	2,64	2,45
	KRK1 -2	1.11.2011	1,774	0,564	5,12	3,63	3,05
		2.11.2011	1,856	0,539	4,90	2,97	2,62
		3.11.2011	1,837	0,544	4,95	3,07	2,39
	KRK1 -3	1.11.2011	1,496	0,668	6,08	3,43	3,14
		2.11.2011	1,508	0,663	6,03	3,09	1,48
		3.11.2011	1,490	0,671	6,10	2,77	2,83
CZ, Krušné Mts, Boží Dar	1+P	7.7.2010	1,248	0,801	7,28	2,20	2,06
	2+P	8.7.2010	1,233	0,811	7,37	2,37	2,52
	3+P	8.7.2010	1,243	0,805	7,31	2,35	2,42
	4+P	8.7.2010	1,248	0,801	7,28	2,78	2,71
	5+P	8.7.2010	1,236	0,809	7,35	2,71	2,13
	6+P	8.7.2010	1,237	0,808	7,35	3,09	2,10
CZ, Krušné Mts, Horní Blatná	1+P	7.7.2010	1,442	0,693	6,30	3,08	2,15
	2+P	7.7.2010	1,415	0,707	6,42	3,44	2,28
	3+P	7.7.2010	1,424	0,702	6,38	3,67	2,69
CZ, Krušné Mts, Kalek	KAL 1	5.9.2011	1,660	0,602	5,48	2,34	2,07
		1.11.2011	1,666	0,600	5,46	3,25	1,83
		3.11.2011	1,700	0,588	5,35	2,87	2,85
	KAL 2	5.9.2011	1,680	0,595	5,41	3,03	2,10
		1.11.2011	1,693	0,591	5,37	2,68	2,35
		3.11.2011	1,683	0,594	5,40	2,96	2,50
	RYŽ 1	31.10.2011	1,676	0,597	5,42	2,78	2,15
		1.11.2011	1,673	0,598	5,43	2,93	2,50
		3.11.2011	1,682	0,595	5,40	2,85	2,50
	RYŽ 2	5.9.2011	1,266	0,790	7,18	2,73	2,54

CZ, Krušné Mts, Ryžovna		1.11.2011	1,267	0,789	7,17	2,97	2,51
		3.11.2011	1,297	0,771	7,01	2,23	1,64
	RYŽ 3	5.9.2011	1,254	0,797	7,25	2,11	2,48
		1.11.2011	1,261	0,793	7,21	2,99	2,62
		3.11.2011	1,273	0,786	7,14	2,83	2,44
CZ, Krušné Mts, Výsluní	VÝSL 1	5.9.2011	1,694	0,590	5,37	2,86	2,36
		1.11.2011	1,703	0,587	5,34	2,57	2,12
		2.11.2011	1,725	0,580	5,27	2,95	1,77
	VÝSL 2	5.9.2011	1,673	0,598	5,43	2,87	2,19
		1.11.2011	1,681	0,595	5,41	2,71	2,11
		3.11.2011	1,672	0,598	5,44	3,20	2,35
	VÝSL 3	1.11.2011	1,693	0,591	5,37	2,82	2,44
		2.11.2011	1,695	0,590	5,36	2,72	2,86
		3.11.2011	1,702	0,588	5,34	2,82	2,24
CZ, Orlické Mts, Sedloňov, Polom hill	POL 1+P	2.8.2010	1,795	0,557	5,06	2,88	1,93
	POL 2	2.8.2010	1,826	0,548	4,98	3,19	2,36
	POL 3	2.8.2010	1,794	0,557	5,07	3,49	2,95
	POL 4	2.8.2010	1,822	0,549	4,99	2,82	2,80
	POL 5	2.8.2010	1,847	0,541	4,92	3,90	2,61
	POL 5	4.11.2010	1,825	0,548	4,98	3,63	2,37
	POL 5	3.3.2011	1,847	0,541	4,92	1,88	1,71
	POL 6	2.8.2010	1,828	0,547	4,97	4,05	2,45
	POL 7	2.8.2010	1,743	0,574	5,22	3,82	3,11
	POL 8	2.8.2010	1,830	0,546	4,97	4,41	3,27
	POL 9	2.8.2010	1,826	0,548	4,98	4,05	2,86
CZ, Radhošťské Beskydy Mts, Krásná hill	BESK1	8.12.2010	1,660	0,602	5,48	2,25	1,78
	BESK2	8.12.2010	1,675	0,597	5,43	2,89	2,25
	BESK3	8.12.2010	1,595	0,627	5,70	3,60	2,05
	BESK4	8.12.2010	1,669	0,599	5,45	2,37	1,74
	BESK5	8.12.2010	1,702	0,588	5,34	2,18	1,82
CZ, Sklené nad Oslavou	SK1+P	26.10.2009	1,658	0,603	5,48	2,50	2,10
	SK1	10.12.2009	1,688	0,592	5,39	3,41	2,08
	SK2+P	26.10.2009	1,667	0,600	5,45	2,92	2,09
	SK3+P	26.10.2009	1,638	0,611	5,55	2,32	1,95
	SK3	10.12.2009	1,656	0,604	5,49	2,67	1,76
CZ, Slavkovský les, Bečov nad Teplou	BEČ 1+P	14.9.2010	1,697	0,589	5,36	3,62	2,11
	BEČ 2	14.9.2010	1,679	0,596	5,41	4,65	2,92
	BEČ 3	14.9.2010	1,675	0,597	5,43	4,29	2,34
	BEČ 4	14.9.2010	1,648	0,607	5,52	3,74	2,97
	BEČ 5	14.9.2010	1,667	0,600	5,45	4,74	2,71
	BEČ 6	14.9.2010	1,421	0,704	6,40	3,99	2,33
	BEČ 7	14.9.2010	1,415	0,707	6,42	2,69	2,57
	BEČ 8	14.9.2010	1,688	0,592	5,39	4,87	2,85
	BEČ 9	14.9.2010	1,627	0,615	5,59	3,49	2,40
	BEČ 10	14.9.2010	1,632	0,613	5,57	3,87	2,91
	BEČ 11	14.9.2010	1,631	0,613	5,57	4,96	3,54
	BEČ 12	14.9.2010	1,618	0,618	5,62	3,76	2,90
	BEČ 13	14.9.2010	1,654	0,605	5,50	3,64	2,90
	BUC 01+P	10.9.2010	1,244	0,804	7,31	4,80	4,13
	BUC 02	10.9.2010	1,292	0,774	7,04	3,85	2,66

CZ, Šumava, Bučina	BUC 03	10.9.2010	1,299	0,770	7,00	3,63	3,40
	BUC 04	10.9.2010	1,266	0,790	7,18	3,39	3,59
	BUC 05	10.9.2010	1,272	0,786	7,15	3,57	3,68
	BUC 06	10.9.2010	1,257	0,796	7,23	3,49	3,23
	BUC 07	10.9.2010	1,294	0,773	7,02	2,89	3,01
	BUC 08	10.9.2010	1,305	0,766	6,97	2,18	3,72
	BUC 09	10.9.2010	1,315	0,760	6,91	2,43	2,73
	BUC 1	18.10.2011	1,357	0,737	6,70	2,64	1,29
	BUC 2	18.10.2011	1,296	0,772	7,01	2,56	1,64
	BUC 3	18.10.2011	1,242	0,805	7,32	2,49	2,25
	BUC 4	18.10.2011	1,273	0,786	7,14	2,54	2,15
	BUC 5	18.10.2011	1,413	0,708	6,43	3,03	2,59
	BUC 6	18.10.2011	1,247	0,802	7,29	2,76	2,43
	BUC 7	18.10.2011	1,382	0,724	6,58	2,38	2,27
CZ, Šumava, Český Rudolec	CRU 01	22.6.2010	1,640	0,610	5,54	3,70	2,01
	CRU 02	22.6.2010	1,648	0,607	5,52	4,54	1,98
	CRU 03	22.6.2010	1,631	0,613	5,57	5,78	2,35
	CRU 04	22.6.2010	1,662	0,602	5,47	3,80	2,37
	CRU 05	22.6.2010	1,620	0,617	5,61	4,90	2,47
	CRU 06	22.6.2010	1,598	0,626	5,69	5,22	2,80
CZ, Šumava, Hůrka	HUR 01+P	9.9.2010	1,266	0,790	7,18	3,99	3,54
	HUR 02	9.9.2010	1,326	0,754	6,86	4,55	3,46
	HUR 03	9.9.2010	1,239	0,807	7,34	5,06	4,08
	CBS 01A	9.7.2010	1,384	0,723	6,57	3,55	2,17
	CBS 01B	9.7.2010	1,431	0,699	6,35	2,61	2,96
	CBS 02	9.7.2010	1,253	0,798	7,25	2,66	2,42
	CBS 03	9.7.2010	1,254	0,797	7,25	2,58	1,74
	CBS 04	9.7.2010	1,399	0,715	6,50	2,63	1,97
	CBS 05	9.7.2010	1,657	0,604	5,49	2,75	1,98
	CBS 06	9.7.2010	1,651	0,606	5,51	3,39	2,26
	CBS 07	9.7.2010	1,437	0,696	6,33	2,47	2,01
	CBS 08	9.7.2010	1,264	0,791	7,19	2,36	2,31
	CBS 09	9.7.2010	1,254	0,797	7,25	2,86	1,95
	CBS 10	9.7.2010	1,635	0,612	5,56	3,06	2,06
	ZAD 2-1	18.10.2011	1,411	0,709	6,44	2,42	2,04
		24.10.2011	1,441	0,694	6,31	2,52	2,31
		2.11.2011	1,422	0,703	6,39	2,55	1,60
	ZAD 2-2	24.10.2011	1,455	0,687	6,25	2,21	1,86
		31.10.2011	1,435	0,697	6,33	2,12	2,10
		2.11.2011	1,455	0,687	6,25	2,78	2,17
	ZAD 2-3	18.10.2011	1,259	0,794	7,22	2,38	2,15
		31.10.2011	1,256	0,796	7,24	2,26	2,11
		2.11.2011	1,279	0,782	7,11	2,81	2,99
	ZAD 2-4	18.10.2011	1,388	0,720	6,55	2,36	2,06
		31.10.2011	1,418	0,705	6,41	2,68	2,73
		2.11.2011	1,403	0,713	6,48	2,93	2,85
	ZAD 2-5	18.10.2011	1,429	0,700	6,36	2,89	2,72
		24.10.2011	1,435	0,697	6,33	2,27	1,94
		2.11.2011	1,430	0,699	6,36	2,74	2,23
	ZAD 2-6	18.10.2011	1,573	0,636	5,78	1,66	2,16

CZ, Šumava, Churáňov		31.10.2011	1,645	0,608	5,53	2,71	2,03
		2.11.2011	1,595	0,627	5,70	2,83	2,42
	ZAD 2-7	18.10.2011	1,294	0,773	7,02	1,81	2,31
		31.10.2011	1,270	0,787	7,16	2,47	2,36
		2.11.2011	1,271	0,787	7,15	2,44	2,43
	ZAD 2-8	18.10.2011	1,681	0,595	5,41	2,27	2,22
		31.10.2011	1,661	0,602	5,47	2,93	2,28
		2.11.2011	1,671	0,598	5,44	2,84	2,09
	ZAD 2-9	18.10.2011	1,425	0,702	6,38	2,08	1,97
		31.10.2011	1,400	0,714	6,49	2,41	2,33
		2.11.2011	1,486	0,673	6,12	2,78	2,00
	ZAD 2-10	18.10.2011	1,449	0,690	6,27	1,94	1,92
		31.10.2011	1,423	0,703	6,39	2,55	2,79
		2.11.2011	1,478	0,677	6,15	2,62	2,22
	ZAD 2-11	18.10.2011	1,573	0,636	5,78	3,32	2,85
		31.10.2011	1,626	0,615	5,59	2,72	2,43
		2.11.2011	1,627	0,615	5,59	3,32	2,70
	ZAD 2-12	18.10.2011	1,437	0,696	6,33	2,00	1,99
		24.10.2011	1,428	0,700	6,37	2,58	1,97
		31.10.2011	1,424	0,702	6,38	2,26	2,25
	ZAD 2-13	1.11.2011	1,206	0,829	7,54	3,00	2,77
		2.11.2011	1,232	0,812	7,38	2,97	2,24
		3.11.2011	1,248	0,801	7,28	2,29	1,68
	ZAD 2-14	1.11.2011	1,220	0,820	7,45	2,39	1,86
		2.11.2011	1,232	0,812	7,38	2,97	2,24
		3.11.2011	1,231	0,812	7,38	2,70	1,77
	ZAD 2-15	1.11.2011	1,407	0,711	6,46	2,85	2,14
		2.11.2011	1,403	0,713	6,48	2,56	2,90
		3.11.2011	1,415	0,707	6,42	2,89	1,86
	ZAD 2-16	1.11.2011	1,429	0,700	6,36	2,90	2,26
		2.11.2011	1,429	0,700	6,36	2,90	2,26
		3.11.2011	1,393	0,718	6,53	2,98	2,65
	ZAD 2-17	1.11.2011	1,381	0,724	6,58	3,06	2,22
		2.11.2011	1,388	0,720	6,55	2,56	2,21
		3.11.2011	1,383	0,723	6,57	2,87	1,97
	ZAD 2-18	1.11.2011	1,613	0,620	5,64	3,31	2,81
		2.11.2011	1,653	0,605	5,50	2,99	2,22
		3.11.2011	1,553	0,644	5,85	3,84	2,50
	ZAD 2-19	1.11.2011	1,241	0,806	7,32	2,71	2,04
		2.11.2011	1,262	0,792	7,20	2,56	2,32
		3.11.2011	1,192	0,839	7,63	2,98	2,56
	KLA 01+P	8.7.2010	1,607	0,622	5,66	3,72	2,70
	KLA 02	8.7.2010	1,629	0,614	5,58	3,75	2,69
	KLA 03	8.7.2010	1,631	0,613	5,57	3,60	2,48
	KLA 04	8.7.2010	1,605	0,623	5,66	3,42	2,50
	KLA 05	8.7.2010	1,603	0,624	5,67	3,23	2,54
	KLA 06	8.7.2010	1,604	0,623	5,67	2,98	2,47
	KLA 07	9.7.2010	1,645	0,608	5,53	3,19	1,93
	KLA 08	9.7.2010	1,602	0,624	5,67	3,86	3,70
	KLA 09	9.7.2010	1,590	0,629	5,72	3,10	2,87

CZ, Šumava, Klášterec	KLA 10	9.7.2010	1,624	0,616	5,60	3,18	2,50
	KLA 1	18.10.2011	1,650	0,606	5,51	2,78	1,86
		31.10.2011	1,611	0,621	5,64	2,55	2,09
		2.11.2011	1,650	0,606	5,51	3,06	2,59
	KLA 2	18.10.2011	1,603	0,624	5,67	3,27	2,16
		31.10.2011	1,602	0,624	5,67	2,94	2,98
		2.11.2011	1,624	0,616	5,60	2,73	2,38
	KLA 3	18.10.2011	1,641	0,609	5,54	2,85	2,31
		31.10.2011	1,611	0,621	5,64	3,13	3,02
		2.11.2011	1,648	0,607	5,52	2,54	2,79
	KLA 4	24.10.2011	1,621	0,617	5,61	2,08	2,21
		31.10.2011	1,618	0,618	5,62	2,41	2,82
		2.11.2011	1,643	0,609	5,53	2,92	2,62
	KLA 5	24.10.2011	1,626	0,615	5,59	2,42	2,03
		31.10.2011	1,672	0,598	5,44	3,04	2,65
		2.11.2011	1,653	0,605	5,50	3,42	3,46
CZ, Šumava, Knížecí pláně	KNI 01+P	9.7.2010	1,427	0,701	6,37	2,88	2,18
	KNI 02	9.7.2010	1,264	0,791	7,19	3,00	2,56
	KNI 03	9.7.2010	1,712	0,584	5,31	3,24	2,38
	KNI 04	9.7.2010	1,802	0,555	5,04	3,11	2,28
	KNI 05	9.7.2010	1,813	0,552	5,01	3,10	2,49
	KNI 06	9.7.2010	1,809	0,553	5,02	3,56	2,92
	KNI 07	9.7.2010	1,264	0,791	7,19	2,29	2,26
	KNI 08	9.7.2010	1,254	0,797	7,25	2,39	2,07
	KNI 09	9.7.2010	1,231	0,812	7,38	2,52	2,26
	KNI 10	9.7.2010	1,241	0,806	7,32	2,40	2,14
CZ, Šumava, Ktiš	KTI 01+P	12.7.2010	1,686	0,593	5,39	3,02	2,93
	KTI 02	12.7.2010	1,688	0,592	5,39	5,21	3,73
	KTI 03	12.7.2010	1,720	0,581	5,28	4,75	3,73
CZ, Šumava, Kubova huť	KUB 01	1.6.2010	1,858	0,538	4,89	3,19	2,49
	KUB 02	2.6.2010	1,762	0,568	5,16	3,99	3,29
	KUB 03	2.6.2010	1,265	0,791	7,19	4,09	3,01
	KUB 04	2.6.2010	1,240	0,806	7,33	4,79	4,18
	KUB 05	2.6.2010	1,416	0,706	6,42	5,10	3,51
	KUB 06	1.6.2010	1,270	0,787	7,16	3,21	2,65
	KUB 07	2.6.2010	1,427	0,701	6,37	3,83	3,31
	KUB 08/1	2.6.2010	1,698	0,589	5,35	3,81	2,80
	KUB 08/2	2.6.2010	1,840	0,543	4,94	3,61	2,89
	KUB 08/3	1.6.2010	1,684	0,594	5,40	2,59	2,04
	KUB 09	1.6.2010	1,659	0,603	5,48	3,46	2,60
	KUB 10	1.6.2010	1,274	0,785	7,14	2,77	2,42
	KUB 11	12.7.2010	1,257	0,796	7,23	1,70	2,51
	KUB 12	12.7.2010	1,250	0,800	7,27	2,00	2,71
	KUB 13	12.7.2010	1,435	0,697	6,33	3,27	2,19
	KUB 14a	12.7.2010	1,443	0,693	6,30	2,87	3,12
	KUB 14b	12.7.2010	1,446	0,692	6,29	2,28	2,65
	KUB15+P20	12.7.2010	1,717	0,582	5,29	2,71	2,11
	KUB16	12.7.2010	1,415	0,707	6,42	2,88	2,22
	OLS 01+P	13.9.2010	1,242	0,805	7,32	2,82	2,84
	OLS 02	13.9.2010	1,230	0,813	7,39	2,67	2,70

CZ, Šumava, Kvilda - Olšinka	OLS 03	13.9.2010	1,262	0,792	7,20	2,87	2,90
	OLS 04	13.9.2010	1,328	0,753	6,84	2,74	2,20
	OLS 05	13.9.2010	1,242	0,805	7,32	2,09	2,94
	OLS 06	13.9.2010	1,248	0,801	7,28	2,46	1,96
	OLS 07	13.9.2010	1,278	0,782	7,11	2,40	2,75
	OLS 08	13.9.2010	1,254	0,797	7,25	2,46	2,83
	OLS 09	13.9.2010	1,254	0,797	7,25	2,66	3,25
	OLS 10	13.9.2010	1,244	0,804	7,31	2,75	2,69
	OLS 11	13.9.2010	1,433	0,698	6,34	2,70	2,82
	OLS 12	13.9.2010	1,412	0,708	6,44	3,47	3,33
	OLS 13	14.9.2010	1,424	0,702	6,38	2,45	2,33
	OLS 14	14.9.2010	1,242	0,805	7,32	2,17	2,82
	OLS 15	14.9.2010	1,226	0,816	7,41	2,10	2,65
	OLS 16	14.9.2010	1,236	0,809	7,35	2,34	2,93
	OLS 17	14.9.2010	1,228	0,814	7,40	2,23	3,01
	OLS 18	14.9.2010	1,237	0,808	7,35	2,55	3,02
	OLS 19	14.9.2010	1,230	0,813	7,39	2,61	2,71
CZ, Šumava, Kvilda, Tetřev	TET 01+P	9.9.2010	1,235	0,810	7,36	2,36	2,91
	TET 01	4.11.2010	1,267	0,789	7,17	2,42	2,50
	TET 01	3.3.2011	1,266	0,790	7,18	1,82	1,68
	TET 02	9.9.2010	1,257	0,796	7,23	3,16	1,62
	TET 03	9.9.2010	1,255	0,797	7,24	3,45	2,90
	TET 04	9.9.2010	1,247	0,802	7,29	3,43	3,26
	KVIL	18.10.2011	1,211	0,826	7,51	2,09	2,70
		26.10.2011	1,234	0,810	7,37	2,78	2,24
		31.10.2011	1,214	0,824	7,49	3,01	2,32
CZ, Šumava, Laka lake	LAK 01+P	10.9.2010	1,265	0,791	7,19	3,19	2,64
	LAK 02	10.9.2010	1,249	0,801	7,28	3,05	2,66
	LAK 03	10.9.2010	1,318	0,759	6,90	3,98	2,85
	LAK 04	10.9.2010	1,427	0,701	6,37	3,81	3,21
	LAK 05	10.9.2010	1,429	0,700	6,36	3,13	2,81
	LAK 06	10.9.2010	1,282	0,780	7,09	3,04	3,45
CZ, Šumava, Nové Hutě	PRIL 01+P	12.7.2010	1,261	0,793	7,21	2,86	2,45
	PRIL 02	12.7.2010	1,262	0,792	7,20	3,35	2,05
	PRIL 03	12.7.2010	1,670	0,599	5,44	2,90	3,22
	PRIL 04	12.7.2010	1,254	0,797	7,25	3,27	2,68
	PRIL 05	12.7.2010	1,250	0,800	7,27	3,08	2,80
	PRIL 06	12.7.2010	1,459	0,685	6,23	3,44	2,46
	PRIL 07	12.7.2010	1,619	0,618	5,61	3,32	3,91
	PRIL 1	18.10.2011	1,251	0,799	7,27	2,69	2,60
		24.10.2011	1,271	0,787	7,15	2,66	2,03
		1.11.2011	1,255	0,797	7,24	2,97	2,47
	PRIL 2	18.10.2011	1,643	0,609	5,53	2,15	2,42
		24.10.2011	1,650	0,606	5,51	2,75	2,04
		1.11.2011	1,629	0,614	5,58	3,09	2,76
	PRIL 3	18.10.2011	1,647	0,607	5,52	2,90	2,59
		24.10.2011	1,649	0,606	5,51	2,96	2,03
		31.10.2011	1,638	0,611	5,55	2,71	1,93
	PRIL 4	18.10.2011	1,643	0,609	5,53	2,61	2,00
		31.10.2011	1,638	0,611	5,55	2,85	2,52

		2.11.2011	1,622	0,617	5,60	2,70	2,59
	PRIL 5	1.11.2011	1,230	0,813	7,39	2,10	2,77
		2.11.2011	1,259	0,794	7,22	2,71	2,21
		3.11.2011	1,240	0,806	7,33	2,32	2,17
	PRIL 6	1.11.2011	1,608	0,622	5,65	3,06	2,35
		2.11.2011	1,680	0,595	5,41	2,41	2,21
		3.11.2011	1,664	0,601	5,46	2,72	1,88
CZ, Šumava, Ostrá hora	OST 01+P	9.7.2010	1,713	0,584	5,31	2,54	1,82
	OST 02	9.7.2010	1,721	0,581	5,28	2,73	1,91
	OST 03	9.7.2010	1,722	0,581	5,28	2,54	2,12
	OST 04	9.7.2010	1,710	0,585	5,32	3,02	1,83
	OST 05	9.7.2010	1,716	0,583	5,30	3,08	1,94
	OST 06	9.7.2010	1,747	0,572	5,20	2,50	1,97
CZ, Šumava, Vltava´s springs	VLT 01+P	10.9.2010	1,265	0,791	7,19	2,53	2,66
	VLT 02	10.9.2010	1,271	0,787	7,15	3,32	3,30
	VLT 03	10.9.2010	1,234	0,810	7,37	3,04	3,20
	VLT 04	10.9.2010	1,417	0,706	6,41	2,85	3,13
	VLT 05	10.9.2010	1,263	0,792	7,20	3,69	3,11
	VLT 06	10.9.2010	1,441	0,694	6,31	3,69	3,45
	VLT 07	10.9.2010	1,539	0,650	5,91	4,87	3,48
	VLT 07	4.11.2010	1,583	0,632	5,74	3,86	2,40
	VLT 07_2	3.3.2011	1,581	0,633	5,75	4,62	2,11
	VLT 08	10.9.2010	1,441	0,694	6,31	4,03	2,91
CZ, Šumava, Příslop	PRI 01+P	9.7.2010	1,646	0,608	5,52	2,57	1,81
	PRI 02	9.7.2010	1,625	0,615	5,59	2,85	2,09
	PRI 03	9.7.2010	1,634	0,612	5,56	3,16	2,10
	PRI 04 (1.po	9.7.2010	1,661	0,602	5,47	2,89	1,90
	PRI 05	9.7.2010	1,665	0,601	5,46	2,79	1,98
CZ, Šumava, Srní	SRN 01+P	9.9.2010	1,495	0,669	6,08	4,18	3,31
	SRN 01	4.11.2010	1,526	0,655	5,96	3,56	2,30
	SRN 01	3.3.2011	1,521	0,657	5,98	2,63	1,39
	SRN 02	9.9.2010	1,495	0,669	6,08	4,13	3,66
	SRN	24.10.2011	1,491	0,671	6,10	2,67	2,04
		26.10.2011	1,470	0,680	6,18	2,01	2,51
		31.10.2011	1,495	0,669	6,08	2,94	2,17
CZ, Šumava, Stachy	STA 1	8.9.2011	1,420	0,704	6,40	2,12	1,58
	STA 2	8.9.2011	1,401	0,714	6,49	2,86	1,44
	Š1+P	22.10.2009	1,282	0,780	7,09	2,75	2,31
	Š2+P	22.10.2009	1,426	0,701	6,37	2,75	1,91
	Š3+P	22.10.2009	1,261	0,793	7,21	2,31	2,18
	Š4+P	22.10.2009	1,246	0,803	7,30	2,49	2,21
	Š5+P	22.10.2009	1,265	0,791	7,19	2,93	2,47
	Š6+P	22.10.2009	1,243	0,805	7,31	2,65	2,56
	Š7+P	22.10.2009	1,434	0,697	6,34	3,10	1,82
	Š8+P	22.10.2009	1,317	0,759	6,90	3,11	2,35
	Š9+P	22.10.2009	1,240	0,806	7,33	2,66	2,68
	Š10+P	22.10.2009	1,249	0,801	7,28	2,60	2,09
	Š11+P	22.10.2009	1,249	0,801	7,28	2,37	2,09
	2Š1+P	22.10.2009	1,792	0,558	5,07	2,38	2,12
	2Š2+P	22.10.2009	1,277	0,783	7,12	2,98	2,14

CZ, Šumava, Špičák hill	2Š3+P	22.10.2009	1,774	0,564	5,12	2,83	2,08
	2Š4+P	22.10.2009	1,248	0,801	7,28	2,42	1,76
	2Š5+P	22.10.2009	1,274	0,785	7,14	2,91	2,53
	2Š6+P	22.10.2009	1,259	0,794	7,22	2,94	2,10
	2Š7+P	22.10.2009	1,282	0,780	7,09	2,50	2,15
	2Š8+P	22.10.2009	1,438	0,695	6,32	2,51	2,17
	2Š9+P	22.10.2009	1,271	0,787	7,15	2,56	2,59
	2Š10+P	22.10.2009	1,270	0,787	7,16	2,87	2,24
	2Š11+P	22.10.2009	1,244	0,804	7,31	2,62	2,17
	2Š11+P	23.10.2009	1,242	0,805	7,32	2,51	2,14
	2Š12+P	23.10.2009	1,226	0,816	7,41	2,85	2,21
	2Š13+P	23.10.2009	1,237	0,808	7,35	2,98	1,86
	2Š14+P	23.10.2009	1,219	0,820	7,46	2,85	2,60
	2Š15+P	23.10.2009	1,242	0,805	7,32	2,51	2,14
	VCE 01+P	9.7.2010	1,616	0,619	5,63	2,77	2,24
	VCE 02	9.7.2010	1,642	0,609	5,54	2,66	1,92
	VCE 03	9.7.2010	1,421	0,704	6,40	2,78	2,16
	VCE 04	12.7.2010	1,432	0,698	6,35	1,84	2,81
CZ, Šumava, Včelná pod Boubínem	VCE 1	18.10.2011	1,551	0,645	5,86	2,80	2,39
		31.10.2011	1,624	0,616	5,60	3,02	2,93
		1.11.2011	1,604	0,623	5,67	2,60	2,22
	VCE 2	24.10.2011	1,466	0,682	6,20	2,40	2,02
		31.10.2011	1,454	0,688	6,25	2,55	1,60
		1.11.2011	1,453	0,688	6,26	2,56	2,18
	CER 01+P	10.9.2010	1,695	0,590	5,36	3,14	2,11
	CER 02	10.9.2010	1,686	0,593	5,39	3,71	2,60
	CER 03	10.9.2010	1,638	0,611	5,55	4,07	2,34
	CER 04	10.9.2010	1,748	0,572	5,20	4,29	2,07
CZ, Šumava, Vysočina - Čeřínek	CER 04	4.11.2010	1,760	0,568	5,16	2,92	1,78
	CER 04	3.3.2011	1,685	0,593	5,39	3,87	1,81
	CER 05	3.3.2011	1,312	0,762	6,93	3,97	1,69
	CER 05	4.11.2010	1,437	0,696	6,33	2,98	2,42
	CER 05	3.3.2011	1,312	0,762	6,93	3,97	1,69
	Z1+P	23.10.2009	1,224	0,817	7,43	2,48	2,24
	Z2+P	23.10.2009	1,243	0,805	7,31	1,87	2,79
	Z3+P	23.10.2009	1,212	0,825	7,50	2,30	2,18
	Z4+P	23.10.2009	1,244	0,804	7,31	2,56	1,98
CZ, Šumava, Zadov	Z5+P	23.10.2009	1,230	0,813	7,39	2,74	1,89
	Z6+P	23.10.2009	1,222	0,818	7,44	2,47	2,04
	ZAD 1	24.10.2011	1,280	0,781	7,10	2,76	2,14
		31.10.2011	1,253	0,798	7,25	2,40	2,05
		2.11.2011	1,255	0,797	7,24	2,96	2,87
	CPL 01+P	12.7.2010	1,269	0,788	7,16	1,87	2,04
	CPL 02	12.7.2010	1,262	0,792	7,20	2,78	2,67
	CPL 03	12.7.2010	1,270	0,787	7,16	2,18	2,34
CZ, Šumava, Zadov	CPL 04	12.7.2010	1,276	0,784	7,12	3,43	2,33
	CPL 05	12.7.2010	1,261	0,793	7,21	3,12	2,27
	CPL 06	12.7.2010	1,260	0,794	7,21	2,57	2,11
	ZMU 01+P	12.7.2010	1,254	0,797	7,25	3,20	2,47
	ZMU 02	12.7.2010	1,264	0,791	7,19	3,29	2,11

CZ, Šumava, Žlíbský hill	ZLI 01	40330	1,464	0,683	6,21	4,19	2,74
	ZLI 02/1	40330	1,745	0,573	5,21	3,28	2,83
	ZLI 02/2	40330	1,759	0,569	5,17	3,05	2,42
	ZLI 03	40330	1,708	0,585	5,32	3,41	1,67
	ZLI 04	40330	1,765	0,567	5,15	3,03	2,70
	ZLI 05	1.6.2010	1,759	0,569	5,17	2,50	1,78
	ZLI 06	7.6.2010	1,468	0,681	6,19	4,28	3,74
	ZLI 07	1.6.2010	1,762	0,568	5,16	3,68	2,68
	ZLI 08	1.6.2010	1,274	0,785	7,14	2,92	2,45
	ZLI 09/1	1.6.2010	1,753	0,570	5,19	3,26	2,91
	ZLI 09/2	1.6.2010	1,425	0,702	6,38	2,91	2,60
	ZLI 10	1.6.2010	1,748	0,572	5,20	3,20	2,89
	ZLI 11	7.6.2010	1,242	0,805	7,32	5,62	3,78
	ZLI 12	7.6.2010	1,194	0,838	7,61	3,84	4,31
	ZLI 13	7.6.2010	1,510	0,662	6,02	4,63	3,26
	ZLI 14	7.6.2010	1,647	0,607	5,52	5,11	4,08
	ZLI 15/1	7.6.2010	1,744	0,573	5,21	4,68	3,28
	ZLI 15/2	7.6.2010	1,772	0,564	5,13	5,19	4,30
CZ, Terezín, Červenka forest	KB1+P	10.12.2009	1,866	0,536	4,87	2,71	1,85
	KB2+P	10.12.2009	1,669	0,599	5,45	2,59	1,90
	KB3+P	10.12.2009	1,659	0,603	5,48	2,33	1,85
	KB4+P	10.12.2009	1,804	0,554	5,04	3,05	2,37
	KB5+P	10.12.2009	1,666	0,600	5,46	2,95	2,33
	KB6+P	10.12.2009	1,862	0,537	4,88	3,40	2,48
	KB7+P	10.12.2009	1,679	0,596	5,41	3,15	1,89
	KB8+P	10.12.2009	1,823	0,549	4,99	3,06	2,51
	KB9+P	10.12.2009	1,660	0,602	5,48	2,59	1,60
	KB10+P	10.12.2009	1,868	0,535	4,87	2,57	1,60
	KB11+P	10.12.2009	1,883	0,531	4,83	3,33	1,92
	KB12+P	10.12.2009	1,666	0,600	5,46	2,35	1,29
	KB13+P	10.12.2009	1,910	0,524	4,76	3,97	2,90
	KB14+P	15.12.2009	1,838	0,544	4,95	2,58	2,62
	KB15+P	15.12.2009	1,848	0,541	4,92	2,53	1,89
	KB16+P	15.12.2009	1,652	0,605	5,50	2,21	1,38
	KB17+P	15.12.2009	1,821	0,549	4,99	2,06	2,44
	KB18+P	15.12.2009	1,634	0,612	5,56	1,93	1,81
	KB19+P	15.12.2009	1,663	0,601	5,47	2,16	1,69
	KB20+P	15.12.2009	1,644	0,608	5,53	2,53	1,71
	KB21+P	15.12.2009	1,591	0,629	5,71	2,74	1,81
	KB22+P	15.12.2009	1,657	0,604	5,49	2,45	1,86
	KB23+P	15.12.2009	1,837	0,544	4,95	2,43	1,95
	KB24+P	15.12.2009	1,833	0,546	4,96	1,95	1,09
	KB25+P	14.12.2009	1,635	0,612	5,56	2,17	2,97
	KB26+P	15.12.2009	1,828	0,547	4,97	2,28	1,37
	KB27+P	15.12.2009	1,666	0,600	5,46	2,32	1,73
	KB28+P	15.12.2009	1,809	0,553	5,02	3,38	2,17
	KB29+P	14.12.2009	1,790	0,559	5,08	2,08	2,84
	KB30+P	14.12.2009	1,791	0,558	5,08	3,37	2,74
	T1+P	14.12.2009	1,636	0,611	5,56	2,93	2,20
	T2+P	14.12.2009	1,818	0,550	5,00	3,66	2,56

CZ, Terezín, Červenka forest	T3+P	14.12.2009	1,810	0,552	5,02	3,36	2,28
	T4+P	14.12.2009	1,723	0,580	5,28	3,29	1,96
	T5+P	14.12.2009	1,586	0,631	5,73	2,10	2,45
	T6+P	14.12.2009	1,682	0,595	5,40	2,86	2,04
	T7+P	15.12.2009	1,776	0,563	5,12	2,95	1,67
	T8+P	14.12.2009	1,786	0,560	5,09	2,77	2,32
	T9+P	14.12.2009	1,723	0,580	5,28	2,63	1,72
	T10+P	14.12.2009	1,813	0,552	5,01	2,83	2,10
CZ, Větrný Jeníkov	VJ1+P	26.10.2009	1,638	0,611	5,55	2,62	1,97
	VJ1	10.12.2009	1,643	0,609	5,53	3,33	2,03
	VJ2+P	26.10.2009	1,659	0,603	5,48	2,96	2,29
	VJ3+P	26.10.2009	1,700	0,588	5,35	2,97	2,72
	VJ3	10.12.2009	1,674	0,597	5,43	2,78	1,78
	VJ4+P	26.10.2009	1,657	0,604	5,49	2,53	2,27
	VJ5+P	26.10.2009	1,654	0,605	5,50	2,73	2,14
A, Alps, Oberatuern	RAK	18.10.2011	1,206	0,829	7,54	2,85	2,37
A, Hochficht	HOCH1	18.10.2007	1,219	0,820	7,46	2,30	
	HOCH2	18.10.2007	1,236	0,809	7,35	2,76	
	HOCH3	18.10.2007	1,241	0,806	7,32	2,85	
	HOCH4	19.10.2007	1,252	0,799	7,26	2,79	
	HOCH5	19.10.2007	1,255	0,797	7,24	2,28	
	HOCH6	19.10.2007	1,256	0,796	7,24	2,99	
	HOCH7	19.10.2007	1,258	0,795	7,23	3,00	
	HOCH8	19.10.2007	1,261	0,793	7,21	2,40	
	HOCH9	19.10.2007	1,262	0,792	7,20	4,23	
	HOCH10	19.10.2007	1,264	0,791	7,19	2,86	
	HOCH11	19.10.2007	1,264	0,791	7,19	3,15	
	HOCH12	19.10.2007	1,265	0,791	7,19	2,23	
	HOCH13	19.10.2007	1,266	0,790	7,18	3,37	
	HOCH14	19.10.2007	1,267	0,789	7,17	4,08	
	HOCH15	19.10.2007	1,267	0,789	7,17	1,22	
	HOCH16	19.10.2007	1,268	0,789	7,17	4,37	
	HOCH17	19.10.2007	1,268	0,789	7,17	3,20	
	HOCH18	19.10.2007	1,268	0,789	7,17	3,97	
	HOCH19	19.10.2007	1,271	0,787	7,15	2,81	
	HOCH20	19.10.2007	1,272	0,786	7,15	2,74	
	HOCH21	19.10.2007	1,275	0,784	7,13	2,22	
	HOCH22	19.10.2007	1,277	0,783	7,12	4,32	
	HOCH23	19.10.2007	1,279	0,782	7,11	2,46	
	HOCH24	19.10.2007	1,285	0,778	7,07	2,61	
	HOCH25	19.10.2007	1,287	0,777	7,06	2,98	
	HOCH26	19.10.2007	1,292	0,774	7,04	2,85	
	HOCH27	19.10.2007	1,292	0,774	7,04	4,22	
	HOCH28	19.10.2007	1,292	0,774	7,04	4,09	
	HOCH29	19.10.2007	1,297	0,771	7,01	4,12	
	HOCH30	19.10.2007	1,298	0,770	7,00	2,88	
	HOCH31	24.10.2007	1,303	0,767	6,98	2,57	
	HOCH32	24.10.2007	1,308	0,765	6,95	2,78	
	HOCH48	30.10.2007	1,616	0,619	5,63	2,67	
	HOCH49	31.10.2007	1,619	0,618	5,61	2,45	

	HOCH50	31.10.2007	1,630	0,613	5,58	2,35	
	HOCH51	12.11.2007	1,728	0,579	5,26	3,31	
	HOCH52	13.11.2007	1,729	0,578	5,26	3,53	
	HOCH53	13.11.2007	1,742	0,574	5,22	3,50	
	HOCH54	13.11.2007	1,748	0,572	5,20	3,12	
	HOCH55	13.11.2007	1,764	0,567	5,15	2,60	
	HOCH56	14.11.2007	1,766	0,566	5,15	2,23	
	HOCH57	14.11.2007	1,769	0,565	5,14	3,08	
	HOCH58	14.11.2007	1,782	0,561	5,10	4,12	
	HOCH59	14.11.2007	1,796	0,557	5,06	2,82	
	HOCH60	14.11.2007	1,817	0,550	5,00	3,11	
	HOCH61	14.11.2007	1,825	0,548	4,98	3,64	
	HOCH62	14.11.2007	1,828	0,547	4,97	4,16	
	HOCH63	14.11.2007	1,833	0,546	4,96	3,83	
	HOCH64	14.11.2007	1,841	0,543	4,94	3,65	
	HOCH65	14.11.2007	1,848	0,541	4,92	3,63	
	HOCH66	14.11.2007	1,848	0,541	4,92	2,46	
	HOCH67	14.11.2007	1,854	0,539	4,90	3,01	
	HOCH68	14.11.2007	1,863	0,537	4,88	2,90	
	HOCH69	14.11.2007	1,872	0,534	4,86	3,61	
	HOCH70	14.11.2007	1,879	0,532	4,84	4,67	
	HOCH71	14.11.2007	1,883	0,531	4,83	4,86	
	HOCH72	14.11.2007	1,887	0,530	4,82	2,15	
A, Kärnten, Oberkolbnitz, Reisseck-gruppe	12-09-A-1	7.9.2009	1,281	0,781	7,10	2,59	2,62
	12-09-A-2	7.9.2009	1,271	0,787	7,15	2,57	2,57
	12-09-A-3	7.9.2009	1,263	0,792	7,20	2,32	2,61
F, Störsvik	FIN 8-1	8.9.2011	1,673	0,598	5,43	2,26	1,77
	FIN 8-2	8.9.2011	1,671	0,598	5,44	1,91	1,64
F, Uusimaa, Nurmijärvi	FIN 5-1	8.9.2011	1,641	0,609	5,54	2,01	1,12
	FIN 5-2	5.9.2011	1,655	0,604	5,49	2,64	2,52
	FIN 5-3	5.9.2011	1,670	0,599	5,44	2,59	2,38
F, Uusimaa, Nurmijärvi, Kiljava	FIN 3-1	8.9.2011	1,664	0,601	5,46	2,47	1,46
	FIN 3-2	8.9.2011	1,683	0,594	5,40	2,14	1,57
F, Uusimaa, Nurmijärvi, Kiljava	FIN 4-1	6.9.2011	1,678	0,596	5,42	2,59	2,94
	FIN 4-2	6.9.2011	1,743	0,574	5,22	3,10	2,85
F, Uusimaa, Tammisaari	FIN 1-1+P	6.9.2011	1,747	0,572	5,20	2,88	2,63
	FIN 1-2	6.9.2011	1,771	0,565	5,13	2,95	2,75
F, Varsinais-Suomi	FIN 6-1	8.9.2011	1,663	0,601	5,47	2,82	1,46
	FIN 6-2	6.9.2011	1,770	0,565	5,14	2,93	2,75
F, Varsinais-Suomi	FIN 7-1	6.9.2011	1,684	0,594	5,40	2,98	2,98
	FIN 7-2	6.9.2011	1,712	0,584	5,31	2,68	2,47
F, Varsinais-Suomi, Kiikala, Tarvakas	FIN 2	8.9.2011	1,696	0,590	5,36	2,29	1,64
GB, Scotland	SK 1-1	5.9.2011	1,272	0,786	7,15	3,40	2,42
	SK 1-2	8.9.2011	1,252	0,799	7,26	2,07	1,58
	SK 1-3	8.9.2011	1,249	0,801	7,28	2,33	1,44
GB, Scotland	SK 2-1	5.9.2011	1,266	0,790	7,18	3,57	2,62
	SK 2-2	5.9.2011	1,271	0,787	7,15	3,73	3,01
	SK 3-c	5.9.2011	1,279	0,782	7,11	2,40	2,24
	SK 3a - 1	5.9.2011	1,240	0,806	7,33	3,01	3,18

GB, Scotland	SK 3a - 2	5.9.2011	1,241	0,806	7,32	2,88	2,90
	SK 3a - 3	5.9.2011	1,263	0,792	7,20	2,27	2,33
	SK 3b -1	5.9.2011	1,276	0,784	7,12	2,25	2,77
	SK 3b -2	5.9.2011	1,293	0,773	7,03	1,89	2,38
	SK 3d	5.9.2011	1,288	0,776	7,06	2,96	2,88
GB, Scotland	SK 4-2_2	8.9.2011	1,248	0,801	7,28	2,82	1,45
	SK 4-3	6.9.2011	1,278	0,782	7,11	2,28	2,36
	SK 4-4	6.9.2011	1,291	0,775	7,04	3,03	2,58
	SK 4-5	8.9.2011	1,261	0,793	7,21	2,26	1,43
GB, Scotland, Knockhan Crag	VB	24.10.2011	1,256	0,796	7,24	2,19	2,07
N, Alnesvatnet	SC3	7.9.2009	1,280	0,781	7,10	2,51	2,33
N, Dalsvelen	SC5	7.9.2009	1,675	0,597	5,43	2,64	2,61
N, Järvsö	NORC -1	8.9.2011	1,621	0,617	5,61	2,91	1,66
	NORC -2	8.9.2011	1,664	0,601	5,46	2,29	1,60
	NORC -3	8.9.2011	1,662	0,602	5,47	2,86	1,51
N, Kalkovo	SC10	7.9.2009	1,291	0,775	7,04	2,08	1,22
N, Linneset	SC6-1	7.9.2009	1,655	0,604	5,49	1,93	1,36
	SC6-2	7.9.2009	1,649	0,606	5,51	1,77	1,68
	SC6-3	7.9.2009	1,656	0,604	5,49	1,95	1,77
	SC6-4	7.9.2009	1,650	0,606	5,51	2,53	2,24
	SC6-5	7.9.2009	1,674	0,597	5,43	2,24	2,28
	SC6-6	7.9.2009	1,676	0,597	5,42	2,17	1,71
	SC6-7	7.9.2009	1,672	0,598	5,44	2,71	1,90
	SC6-8	7.9.2009	1,654	0,605	5,50	2,59	2,40
	SC6-9	7.9.2009	1,647	0,607	5,52	2,14	1,90
N, between Formofoss and Sandmoen	SC7	7.9.2009	1,283	0,779	7,08	2,33	1,68
N, between Breidsjøen and Fofervatnet	SC9	7.9.2009	1,303	0,767	6,98	2,50	2,31
N, between Dovre and Folldal	SC4	7.9.2009	1,293	0,773	7,03	2,14	1,90
N, between Lesja and Skogbygde	SC8	7.9.2009	1,296	0,772	7,01	2,59	1,89
N, NP Jotunheimen	NORB 1	5.9.2011	1,277	0,783	7,12	2,72	2,31
	NORB 2	5.9.2011	1,259	0,794	7,22	2,86	2,24
N, near NP Rondane	GPS21	10.9.2008	1,277	0,783	7,12	3,00	2,48
	GPS25	10.9.2008	1,258	0,795	7,23	2,87	2,96
	Solkpass	11.9.2008	1,267	0,789	7,17	2,12	2,11
N, Torsefeatn	SC1	7.9.2009	1,286	0,778	7,07	2,42	1,96
N, Trollhsimon	NOR 1	5.9.2011	1,278	0,782	7,11	2,99	2,25
	NOR 2	8.9.2011	1,247	0,802	7,29	2,92	1,67
N, Vik	SC2	7.9.2009	1,287	0,777	7,06	2,35	1,62

Numerical Morphometrics

Central Europe

ID	taxa	v2	v3	v4	v5	v6	v7	v8	v9	v10	v11	v12	v13	v14	v15	v16	v17	GS (2C)
KR 1_27	*D. i.*	1,4	3,0	0,5	1,8	2,1	2,9	0,6	0,6	0,9	1,8	1,4	3,3	0,7	1,5	1,9	1,2	6,01
2Š1	*D. t.*	1,5	2,4	0,4	0,7	1,7	2,3	0,4	0,6	0,4	1,1	1,4	2,4	0,5	0,5	1,4	0,9	5,07
2Š10	*D. a.*	2,2	2,3	0,8	-0,2	2,0	2,1	0,7	0,2	0,8	2,5	2,7	2,3	0,8	0,4	1,7	0,9	7,16
2Š11	*D. a.*	1,3	2,1	0,6	0,4	1,9	2,1	0,5	0,3	0,6	1,7	1,6	2,6	0,9	0,7	1,8	0,9	7,31
2Š12	*D. a.*	2,1	1,9	0,8	0,0	2,2	2,0	0,6	0,2	0,8	2,3	1,1	1,6	0,8	0,4	1,7	0,9	7,32
2Š13	*D. a.*	2,4	2,5	0,7	0,1	2,1	2,5	0,6	0,4	0,8	2,5	1,4	2,2	0,8	0,5	1,7	0,8	7,41
2Š14	*D. a.*	1,5	1,8	0,7	0,5	2,0	2,0	0,7	0,2	0,6	1,9	1,2	1,9	0,8	0,2	1,8	1,0	7,35
2Š15	*D. a.*	1,5	2,2	0,8	0,4	2,3	2,0	0,4	0,5	0,9	2,3	1,4	2,2	0,9	0,6	1,4	0,5	7,46
2Š2	*D. a.*	2,1	2,8	0,7	1,0	2,3	2,8	0,5	0,8	0,7	1,9	1,7	2,1	0,7	0,8	1,3	0,7	7,12
2Š3	*D. t.*	1,3	2,0	0,3	0,6	1,3	1,8	0,5	0,6	0,4	1,4	1,5	1,9	0,5	0,5	1,6	1,1	5,12
2Š4	*D. a.*	1,5	1,9	0,6	0,5	1,7	1,8	0,7	0,3	0,6	2,1	1,6	1,9	0,7	0,3	2,0	1,3	7,28
2Š5	*D. a.*	1,9	2,1	0,7	-0,2	1,5	1,9	0,6	0,2	0,6	1,6	2,2	1,4	0,8	-0,5	1,6	0,9	7,14
2Š6	*D. a.*	2,4	2,2	0,8	-0,3	2,4	2,1	0,8	-0,1	0,5	2,4	2,0	1,9	0,9	0,2	1,9	1,1	7,22
2Š8	*D. i.*	2,0	2,2	0,5	0,2	1,8	2,4	0,6	0,7	0,5	1,9	2,9	1,7	0,6	-0,3	1,6	1,0	6,32
2Š9	*D. a.*	1,2	1,8	0,7	1,7	1,9	2,0	0,5	0,2	0,7	1,9	1,4	1,2	0,8	0,3	1,6	0,8	7,15
2Š9	*D. a.*	1,8	1,6	0,6	-0,4	2,0	1,5	0,4	-0,2	0,5	1,8	2,2	1,5	0,5	-0,3	1,7	1,2	7,15
A_1	*D. a.*	2,3	2,4	0,7	0,1	1,9	2,4	0,0	-1,1	0,8	2,1	2,9	2,1	0,7	0,7	1,5	0,8	7,10
A_2	*D. a.*	1,9	1,9	0,8	-0,4	2,5	2,1	0,0	-0,5	0,9	1,8	1,8	1,6	0,6	0,6	1,5	0,8	7,15
A_3	*D. a.*	2,3	1,8	0,8	-0,5	1,9	1,9	0,0	-0,3	1,0	1,9	2,7	2,1	0,7	0,6	1,4	0,7	7,20
ALP 1	*D. a.*	1,8	1,9	0,7	-0,1	1,7	1,9	0,8	0,0	0,5	2,0	1,5	2,2	0,8	0,7	1,3	0,5	7,23
BD1	*D. a.*	1,8	2,9	0,6	0,5	2,2	3,0	0,5	0,9	0,6	1,9	1,6	2,3	0,7	0,3	1,7	1,8	7,28
BD2	*D. a.*	2,5	2,7	0,7	0,1	2,4	3,3	0,8	1,2	0,9	2,9	2,9	2,7	0,8	0,7	1,8	1,8	7,37
BD3	*D. a.*	2,2	3,8	0,8	1,1	2,3	3,7	0,9	1,3	0,7	2,8	2,1	2,9	0,9	0,7	1,7	1,8	7,31
BD4	*D. a.*	2,0	2,2	0,7	0,3	2,1	2,4	0,7	0,2	0,6	2,0	1,3	2,0	0,9	0,3	2,0	2,1	7,28
BD5	*D. a.*	2,1	2,2	0,6	0,0	2,1	2,2	0,8	0,0	0,7	2,4	1,9	2,3	0,9	-0,2	2,0	2,1	7,35
BD6	*D. a.*	2,1	2,8	0,5	0,4	1,9	2,1	0,6	0,6	0,5	1,9	1,4	2,9	0,7	1,1	1,5	1,5	7,35
BEČ 1	*D. z.*	2,0	3,4	0,5	1,5	2,2	3,0	1,1	0,8	0,8	2,5	1,5	2,6	0,6	0,9	2,3	2,3	5,36
BEČ 10	*D. c.*	1,3	2,7	0,4	1,1	1,5	1,9	0,6	0,5	0,5	2,0	1,3	2,8	0,6	1,0	2,0	2,1	5,57
BEČ 11	*D. c.*	1,0	2,2	0,3	1,3	0,9	2,4	0,4	1,3	0,6	2,3	1,4	1,9	0,5	0,2	1,6	1,7	5,57
BEČ 12	*D. c.*	1,3	1,9	0,3	0,8	1,8	2,9	0,5	0,9	0,6	2,2	1,1	2,0	0,5	0,6	1,5	1,6	5,62
BEČ 13	*D. c.*	1,3	2,5	0,4	0,9	1,5	2,6	0,5	1,0	0,6	2,2	1,5	2,7	0,5	0,9	1,4	1,5	5,50
BEČ 2	*D. c.*	1,3	2,8	0,4	1,1	1,7	3,3	0,7	1,8	0,7	2,0	1,6	3,4	0,8	1,8	1,9	2,2	5,41
BEČ 3	*D. c.*	1,2	2,2	0,4	1,4	1,5	2,5	0,7	0,9	0,7	2,5	1,8	2,8	0,7	1,3	2,3	2,5	5,43
BEČ 4	*D. c.*	1,0	1,7	0,3	0,4	1,6	1,8	0,6	0,2	0,5	2,1	1,4	1,9	0,5	0,8	1,9	2,0	5,52
BEČ 5	*D. c.*	1,0	2,6	0,4	1,2	1,6	2,4	0,6	0,9	0,5	1,9	1,6	2,2	0,5	0,7	1,5	1,5	5,45
BEČ 6	*D. i.*	2,0	2,8	0,6	0,2	2,1	2,6	0,7	0,0	0,6	2,3	2,0	2,6	0,8	0,2	2,0	2,1	6,40
BEČ 7	*D. i.*	2,0	3,1	0,8	0,8	2,1	3,0	0,9	0,5	0,6	2,1	1,9	3,2	0,9	0,7	2,3	2,3	6,42
BEČ 8	*D. c.*	1,5	2,8	0,4	1,3	1,9	2,9	0,9	1,1	1,0	2,1	1,8	3,1	0,8	1,3	2,6	2,8	5,39
BEČ 9	*D. c.*	2,1	3,0	0,4	0,2	2,2	3,3	1,1	1,0	1,0	2,7	1,9	2,1	0,5	0,5	2,4	2,5	5,59
BES 01	*D. a.*	2,4	4,3	0,5	1,8	2,1	4,2	0,5	1,9	0,5	2,1	1,9	4,5	0,6	2,0	1,6	1,7	7,36
BES 02	*D. a.*	2,2	5,0	0,8	2,5	2,2	5,2	0,8	3,0	0,7	2,8	2,5	5,5	0,8	2,2	1,9	1,9	7,37
BES 03	*D. c.*	0,9	3,6	0,3	2,5	1,8	3,5	1,0	2,1	0,9	3,7	1,4	3,9	0,5	2,2	1,5	1,6	5,61
BES 04	*D. a.*	1,8	1,9	0,6	0,2	2,0	2,1	0,5	0,2	0,8	2,0	1,9	2,0	0,3	0,2	1,1	1,0	7,51
BES 05	*D. a.*	2,2	3,8	0,5	1,3	2,2	3,2	0,7	1,5	0,6	2,1	2,2	3,7	0,7	1,8	1,7	1,8	7,34

BES 07	*D. c.*	1,2	3,5	0,5	2,0	1,6	3,6	0,8	2,1	0,8	2,4	1,7	3,9	0,4	1,9	2,2	2,2	5,47
BES 08	*D. c.*	0,7	2,6	0,2	2,0	1,3	2,1	0,7	1,5	0,5	2,5	1,3	2,7	0,5	1,7	1,9	2,1	5,35
BES 09	*D. a.*	2,8	4,6	0,5	2,0	2,8	4,6	0,7	2,6	0,8	3,0	2,6	4,9	0,9	2,5	1,8	2,0	7,22
BESK1	*D. c.*	1,0	2,7	0,3	2,8	1,8	2,6	0,9	1,5	0,9	2,4	1,9	2,9	0,7	1,2	1,8	1,0	5,48
BESK2	*D. c.*	0,8	2,2	0,4	1,2	1,5	2,2	0,4	1,2	0,5	1,8	1,4	2,3	0,4	0,9	1,3	0,9	5,43
BESK3	*D. c.*	0,9	2,7	0,5	1,8	1,3	3,0	0,7	1,8	0,5	1,4	1,3	2,6	0,5	1,3	1,6	1,1	5,70
BESK4	*D. c.*	1,2	2,7	0,3	1,8	1,5	2,9	0,8	1,8	0,8	2,2	1,5	2,8	0,5	1,3	1,5	1,0	5,45
BESK5	*D. c.*	1,3	2,6	0,4	1,2	1,6	2,4	0,7	0,8	0,6	2,0	1,6	2,7	0,6	1,5	1,7	1,1	5,34
Brdy	*D. t.*	2,9	3,1	0,6	0,4	1,8	3,1	0,6	1,7	0,4	1,5	1,7	2,9	0,7	1,0	1,4	1,5	5,05
BUC 01	*D. a.*	2,3	2,3	0,7	0,1	2,1	2,3	0,7	0,0	0,7	2,4	2,6	2,0	0,9	0,1	1,1	1,2	7,27
BUC 02	*D. a.*	2,4	2,8	0,7	0,6	2,3	2,7	1,1	0,6	0,7	3,2	2,0	3,0	0,8	0,3	1,1	1,2	7,04
BUC 03	*D. a.*	2,4	2,8	0,5	0,5	2,5	2,6	0,7	0,2	0,7	2,4	2,1	2,8	0,9	0,5	1,0	1,2	7,00
BUC 04	*D. a.*	2,6	2,4	0,9	0,4	2,1	2,2	0,5	-0,1	0,6	2,2	2,3	2,1	0,8	0,1	2,1	2,1	7,18
BUC 05	*D. a.*	2,3	2,4	0,7	0,1	2,2	2,3	0,7	0,2	0,7	2,1	2,5	2,8	0,9	0,4	1,4	1,5	7,15
BUC 06	*D. a.*	2,3	2,9	0,4	0,6	2,2	2,9	0,7	1,0	0,8	2,8	2,0	3,3	0,8	1,1	1,0	1,2	7,23
BUC 07	*D. a.*	1,6	3,3	0,6	1,5	1,9	3,9	0,9	1,4	0,7	2,3	2,2	3,2	0,9	1,1	2,5	2,6	7,02
BUC 08	*D. a.*	1,8	1,8	0,6	0,3	1,4	1,9	0,8	0,2	0,5	2,1	1,8	1,9	0,8	0,3	2,2	2,3	6,97
BUC 09	*D. a.*	2,2	3,2	0,7	0,5	2,6	2,7	0,8	0,3	0,7	2,4	2,2	2,6	0,8	0,5	2,3	2,4	6,91
BUC1	*D. a.*	2,3	2,7	0,8	0,4	2,1	2,4	1,0	0,2	0,6	2,1	1,7	2,6	1,0	0,7	2,8	1,8	6,70
BUC2	*D. a.*	2,2	3,4	0,8	1,2	2,5	3,7	1,1	1,4	0,6	2,4	1,9	2,6	1,0	1,0	2,9	1,9	7,01
BUC3	*D. a.*	1,9	2,3	0,5	0,2	2,1	2,2	0,7	0,4	0,5	2,2	1,9	2,5	0,7	0,4	2,7	2,0	7,32
BUC4	*D. a.*	2,8	3,3	0,9	0,4	3,2	2,9	0,6	0,5	0,7	2,7	2,2	3,5	1,0	1,2	2,8	1,8	7,14
BUC5	*D. a.*	2,0	2,0	0,6	0,1	2,0	1,7	0,6	0,0	0,6	1,7	1,6	2,1	0,6	0,2	2,2	1,6	6,43
BUC6	*D. a.*	2,7	2,9	0,8	0,3	2,5	2,8	0,7	0,3	0,7	2,5	2,3	2,9	0,9	0,7	2,5	1,6	7,29
BUC7	*D. a.*	2,1	3,2	0,7	1,1	1,8	3,2	0,8	1,6	0,7	2,1	1,9	3,6	1,0	2,1	2,6	1,7	6,58
CBS 01A	*D. i.*	1,5	1,8	0,4	0,3	2,0	1,8	0,8	-0,1	0,6	2,5	1,4	1,8	0,8	0,1	2,3	2,5	6,57
CBS 01B	*D. i.*	1,9	2,6	0,4	0,6	2,0	2,0	0,8	0,6	0,6	2,4	1,4	2,4	0,8	0,7	2,3	2,5	6,35
CBS 02	*D. a.*	2,3	2,9	0,7	0,2	2,1	2,5	0,9	0,0	0,7	2,5	2,1	2,6	0,8	0,2	1,6	1,6	7,25
CBS 03	*D. a.*	2,7	2,2	0,8	-0,3	2,1	2,4	0,5	-0,1	0,7	2,1	2,1	2,1	1,0	-0,1	2,2	2,3	7,25
CBS 04	*D. i.*	1,9	2,7	0,5	1,0	2,1	2,1	0,9	0,2	0,6	2,0	1,7	2,3	0,7	0,7	2,1	2,2	6,50
CBS 05	*D. c.*	0,9	1,8	0,3	0,8	1,1	1,7	0,6	0,5	0,5	1,7	1,2	1,7	0,6	0,4	1,9	2,0	5,49
CBS 06	*D. c.*	1,2	2,7	0,4	1,6	1,7	2,8	0,7	1,5	0,7	2,5	1,0	2,8	0,5	1,5	2,1	2,1	5,51
CBS 07	*D. i.*	1,6	2,8	0,5	1,1	1,8	3,0	0,9	0,6	0,6	1,7	1,7	2,8	0,9	0,7	2,2	2,4	6,33
CBS 09	*D. a.*	1,6	2,3	0,8	0,3	2,0	2,0	0,6	0,5	0,6	2,0	1,9	1,9	0,8	0,4	1,5	1,5	7,25
CBS 10	*D. c.*	0,6	2,3	0,4	1,5	1,7	1,8	0,5	0,4	0,7	1,7	1,3	1,8	0,5	0,7	1,6	1,7	5,56
CER 01	*D. z.*	1,4	3,3	0,3	1,6	1,8	3,1	0,5	1,3	0,5	2,1	1,4	2,6	0,5	0,9	1,3	1,4	5,36
CER 02	*D. z.*	1,4	3,3	0,4	2,0	1,8	3,5	0,6	1,9	0,5	1,6	1,7	3,0	0,7	1,3	1,2	1,4	5,39
CER 03	*D. c.*	0,5	2,9	0,4	1,5	1,5	2,2	0,8	1,2	0,5	2,3	1,7	2,8	0,7	0,6	1,3	1,4	5,55
CER 04	*D. z.*	1,4	1,6	0,4	-0,3	1,3	1,2	0,4	-0,2	0,4	1,3	1,3	1,4	0,5	-0,1	1,4	1,4	5,20
CER 05	*D. i.*	1,5	2,2	0,5	0,5	1,6	2,2	0,9	0,4	0,6	2,0	1,7	2,5	0,7	0,8	1,5	1,6	6,37
CPL 01	*D. a.*	2,3	3,0	0,8	0,4	2,3	3,1	0,9	1,4	1,0	2,8	2,2	3,1	1,0	0,4	1,5	1,6	7,16
CPL 02	*D. a.*	1,9	2,3	0,6	0,1	2,0	2,2	0,8	0,6	0,7	2,7	1,5	2,2	0,8	0,1	1,6	1,7	7,20
CPL 03	*D. a.*	2,4	2,3	0,7	-0,3	2,2	2,3	0,8	0,1	0,8	2,4	2,4	2,5	0,7	-0,2	1,3	1,3	7,16
CPL 04	*D. a.*	2,9	2,8	0,9	-0,1	2,9	2,9	0,4	0,5	1,0	2,5	2,1	2,9	0,9	0,4	1,6	1,6	7,12
CPL 05	*D. a.*	0,9	1,4	0,6	0,0	2,0	1,4	0,7	-0,3	0,6	2,1	1,4	1,3	0,7	-0,1	2,2	2,3	7,21
CPL 06	*D. a.*	1,1	1,3	0,5	-0,3	1,7	1,2	0,5	-0,3	0,6	1,9	1,3	1,0	0,5	-0,5	1,6	1,6	7,21
CRU 01	*D. c.*	0,9	3,1	0,5	1,5	1,4	3,0	0,6	1,7	0,7	2,0	1,4	2,6	0,6	0,9	1,8	1,9	5,54
CRU 02	*D. c.*	0,5	2,6	0,5	2,0	1,3	2,7	0,6	1,9	0,4	2,3	1,4	2,6	0,6	1,3	1,7	1,8	5,52
CRU 03	*D. c.*	0,9	3,0	0,4	1,9	1,0	2,7	0,6	1,5	0,6	2,0	1,2	3,3	0,6	2,3	1,9	2,0	5,57
CRU 04	*D. c.*	0,7	3,2	0,3	1,9	1,7	2,9	0,3	1,7	0,5	2,7	1,1	2,9	0,4	1,9	1,8	1,8	5,47

CRU 05	*D. c.*	0,9	2,8	0,4	1,9	1,5	2,9	0,6	1,5	0,6	2,9	1,3	2,7	0,7	1,1	1,9	2,1	5,61
CRU 06	*D. c.*	0,6	3,3	0,3	2,7	1,4	3,8	0,8	2,9	0,6	2,4	1,1	3,2	0,5	2,2	1,9	2,0	5,69
HB1	*D. i.*	2,5	5,0	0,7	2,8	2,3	4,1	1,3	1,9	0,7	2,7	1,6	5,0	1,1	2,4	2,8	3,0	6,30
HB2	*D. i.*	0,9	3,0	0,7	0,9	1,7	2,4	0,8	0,7	0,8	2,3	1,7	4,9	1,2	2,6	2,6	2,9	6,42
HB3	*D. i.*	1,7	3,0	0,7	0,7	1,7	2,8	0,8	1,1	0,8	2,4	2,2	2,9	0,8	1,5	2,3	2,4	6,38
HUR 01	*D. a.*	2,3	2,0	0,7	-0,3	2,1	1,9	0,5	-0,2	0,6	2,5	1,9	1,5	0,8	0,0	2,1	2,2	7,18
HUR 02	*D. a.*	1,9	1,9	0,6	0,2	2,1	2,1	0,8	0,0	0,6	2,4	1,7	2,0	0,8	0,0	2,2	2,3	6,86
HUR 03	*D. a.*	2,3	2,7	0,5	0,8	2,1	2,6	0,7	0,6	0,6	2,6	1,8	2,7	0,7	0,6	1,0	1,1	7,34
Je 1_1	*D. a.*	1,9	2,4	0,6	0,6	2,1	2,6	0,8	0,4	0,8	2,5	2,2	2,5	0,8	0,2	1,0	1,1	7,68
Je 1_2	*D. a.*	2,4	3,0	0,6	0,5	2,0	2,7	0,6	0,8	0,6	2,0	2,2	2,8	0,7	1,1	1,3	1,3	7,61
Je 1_3	*D. a.*	2,5	5,9	0,6	2,7	2,6	5,6	0,6	3,4	0,7	2,8	2,1	6,1	0,8	3,1	1,0	1,1	7,67
Je 1_4	*D. a.*	2,4	3,2	0,6	0,7	2,1	3,4	0,9	0,8	0,9	2,4	1,8	2,8	0,8	1,0	1,3	1,4	7,44
Je 2_1	*D. a.*	2,4	1,6	0,9	-0,6	1,8	1,6	0,5	-0,3	0,9	2,1	2,4	1,8	0,9	-0,5	1,7	1,7	7,66
Je 3_1	*D. a.*	2,2	2,7	0,8	0,4	2,0	2,5	0,4	0,3	0,7	1,9	1,9	2,4	0,8	0,3	0,9	0,9	7,65
Je 3_2	*D. a.*	2,8	3,0	0,9	0,5	3,3	2,9	0,5	-0,3	0,8	1,5	3,0	3,2	0,8	0,3	1,4	1,3	7,40
Je 3_3	*D. a.*	2,3	2,3	0,8	0,0	2,2	2,1	0,4	0,0	0,7	1,7	2,5	2,3	0,8	-0,2	1,4	1,5	7,57
Je 3_4	*D. a.*	2,6	3,8	1,2	1,3	2,4	3,5	0,4	1,6	0,8	2,1	2,2	3,2	1,0	1,4	1,3	1,2	7,37
Je 4_1	*D. a.*	1,9	2,2	0,7	0,3	2,0	2,4	0,5	0,4	0,7	1,8	1,7	2,4	0,6	0,6	1,1	1,1	7,49
Je 4_2	*D. a.*	2,1	2,0	0,8	-0,1	1,8	2,0	0,6	-0,1	0,7	2,1	1,9	1,6	0,7	-0,3	0,8	0,8	7,49
Je 5_1	*D. a.*	2,6	3,0	0,8	0,8	2,7	3,3	1,0	0,9	1,0	2,7	2,3	2,8	1,0	0,8	3,1	3,2	7,25
Je 5_2	*D. a.*	2,1	2,6	0,8	0,3	2,1	2,5	0,4	0,3	0,6	2,1	2,0	3,0	0,9	0,5	1,5	1,6	7,41
Je 5_3	*D. a.*	2,3	2,9	0,8	0,2	2,3	2,4	0,8	0,4	0,8	2,5	2,3	2,6	1,0	0,0	2,3	2,4	7,52
Je 5_4	*D. a.*	2,0	2,6	0,9	0,2	2,3	2,6	0,5	0,0	0,6	2,3	2,5	2,5	1,0	0,1	1,9	1,9	7,39
Je 5_6	*D. a.*	1,8	2,1	0,8	0,1	1,9	2,3	0,4	0,4	0,6	1,7	2,1	2,2	0,8	-0,2	1,5	1,5	7,34
Je 6_1	*D. a.*	2,3	3,7	0,5	1,3	2,3	3,7	0,8	1,6	0,9	2,8	1,7	3,4	0,7	2,0	0,9	1,0	7,48
Je 6_10	*D. a.*	2,6	3,8	0,6	1,4	2,3	3,3	0,8	1,3	0,7	2,7	2,0	3,4	0,8	2,0	1,5	1,6	7,58
Je 6_2	*D. a.*	2,4	2,6	0,9	0,0	2,4	2,7	0,7	0,3	0,7	2,5	2,6	2,7	0,9	0,6	1,3	1,3	7,38
Je 6_3	*D. a.*	2,1	2,3	0,6	0,0	2,0	2,6	0,5	0,4	0,6	2,0	2,5	2,6	0,9	0,4	1,7	1,9	7,46
Je 6_4	*D. a.*	1,5	1,4	0,6	-0,2	1,8	1,4	0,5	-0,1	0,6	2,4	1,5	1,6	0,7	0,0	1,7	1,7	7,21
Je 6_5	*D. a.*	2,5	3,8	0,7	1,4	2,3	3,8	0,8	1,2	0,8	3,2	2,3	3,9	0,9	1,6	1,4	1,5	7,30
Je 6_6	*D. a.*	2,1	2,6	0,7	0,4	2,0	2,3	0,7	0,9	0,8	2,8	1,9	2,3	0,8	1,2	1,1	1,1	7,43
Je 6_7	*D. a.*	2,3	2,4	0,7	0,1	2,3	2,6	0,8	0,0	0,7	2,8	2,3	2,8	0,9	0,5	2,5	2,6	7,40
Je 6_8	*D. a.*	1,7	2,0	0,7	0,3	1,9	1,9	0,4	0,4	0,7	2,4	1,9	2,0	0,7	0,0	0,9	0,9	7,47
Je 6_9	*D. a.*	2,0	2,8	0,7	0,5	2,4	2,7	0,5	0,5	0,8	2,4	2,0	2,5	0,8	0,6	1,1	1,2	7,59
K1	*D. t.*	1,4	2,1	0,6	0,7	1,4	2,0	0,0	0,4	0,4	1,5	1,4	8,6	0,6	0,5	1,5	0,8	4,86
K2	*D. c.*	0,5	2,4	0,3	2,0	1,2	2,7	0,0	1,2	0,8	2,1	1,5	2,5	0,5	1,0	1,7	1,2	5,35
K3	*D. t.*	1,7	1,8	0,6	0,7	1,3	2,5	0,0	0,4	0,4	1,3	1,4	1,9	0,7	0,6	1,5	0,8	4,85
K4	*D. t.*	1,1	2,7	0,5	1,2	1,4	2,8	0,0	0,9	0,5	1,1	1,3	2,1	0,7	1,2	1,3	0,6	4,93
K5	*D. c.*	1,3	3,3	0,5	2,5	1,5	3,4	0,0	1,5	0,9	2,6	1,4	3,1	0,6	0,8	2,1	1,5	5,48
K6	*D. t.*	1,2	2,5	0,5	1,1	1,4	2,4	0,0	0,7	0,4	1,4	1,2	2,4	0,6	0,8	1,3	0,7	4,85
K7	*D. t.*	1,2	2,3	0,4	1,0	1,3	2,5	0,0	1,1	0,4	1,3	1,4	2,3	0,3	1,3	1,1	0,8	4,93
K8	*D. t.*	1,2	1,9	0,4	0,6	1,3	2,3	0,0	0,9	0,3	1,3	0,9	1,9	0,4	0,6	1,1	0,6	4,99
KAL 1	*D. c.*	1,0	1,9	0,6	0,7	1,4	1,5	0,6	0,4	0,7	1,8	1,4	2,1	0,7	0,3	2,0	1,3	5,43
KAL 2	*D. c.*	1,0	2,2	0,5	1,0	1,3	2,3	0,4	0,7	0,6	2,2	1,3	1,9	0,5	0,6	1,8	1,2	5,39
KB 1	*D. t.*	1,3	2,1	0,5	0,9	1,4	2,2	0,4	0,7	0,4	1,4	1,5	2,2	0,5	0,8	1,3	0,9	4,87
KB 10	*D. t.*	1,4	2,6	0,4	1,2	1,4	2,8	0,3	1,3	0,4	1,3	1,4	3,0	0,7	1,5	1,5	0,8	4,87
KB 11	*D. t.*	1,6	3,9	0,3	2,2	1,6	3,4	0,3	1,7	0,5	0,9	1,4	3,5	0,4	2,1	1,4	1,0	4,83
KB 12	*D. c.*	0,5	2,0	0,4	1,3	1,3	2,1	0,5	1,0	0,5	2,0	1,2	2,6	0,6	1,3	1,6	1,0	5,46
KB 13	*D. t.*	1,3	2,2	0,5	0,7	1,5	2,2	0,4	0,7	0,4	1,2	1,5	2,3	0,6	0,8	1,5	0,8	4,73

KB 14	*D. t.*	1,4	2,1	0,5	0,8	1,4	2,3	0,3	1,1	0,4	1,3	1,4	2,4	0,7	1,3	1,1	0,5	4,95
KB 15	*D. t.*	1,5	3,2	0,7	1,7	1,5	3,0	0,3	1,5	0,5	1,2	1,5	2,9	0,8	1,6	1,4	0,6	4,92
KB 17	*D. t.*	1,3	2,6	0,5	1,2	1,6	2,9	0,4	1,4	0,4	1,2	1,3	2,8	0,6	1,4	1,4	0,7	4,99
KB 18	*D. c.*	0,8	3,1	0,2	2,4	1,5	3,2	0,4	1,9	1,0	2,5	1,2	3,0	0,8	1,3	2,0	1,2	5,56
KB 19	*D. c.*	0,8	2,9	0,4	1,8	1,4	2,9	0,4	1,7	0,7	2,1	1,4	3,0	0,8	1,8	2,0	1,1	5,47
KB 2	*D. c.*	0,9	2,9	0,3	1,4	1,5	2,9	0,7	1,6	0,9	2,6	1,2	3,6	0,7	2,1	2,2	1,5	5,45
KB 20	*D. c.*	0,8	2,9	0,3	1,7	1,4	2,1	0,3	0,9	0,8	2,3	1,1	2,4	0,6	0,9	1,7	1,1	5,53
KB 21	*D. c.*	0,7	2,6	0,3	1,8	1,2	2,8	0,3	1,7	0,5	1,7	1,3	2,5	0,6	1,2	1,5	0,9	5,71
KB 22	*D. c.*	0,9	1,8	0,3	0,9	1,2	1,8	0,6	0,7	0,5	2,1	1,3	1,6	0,7	0,6	1,8	1,1	5,49
KB 23	*D. t.*	1,3	2,2	0,6	0,8	1,3	2,0	0,4	0,7	0,4	1,1	1,3	2,2	0,3	1,0	1,4	1,1	4,95
KB 24	*D. t.*	1,3	2,3	0,5	1,2	1,5	2,3	0,3	0,6	0,4	1,3	1,3	2,5	0,5	2,4	1,3	0,7	4,96
KB 25	*D. c.*	0,8	2,8	0,3	1,6	1,2	3,1	0,6	1,4	0,5	1,8	1,5	2,5	0,6	1,0	1,9	1,3	5,56
KB 26	*D. t.*	1,4	2,3	0,5	1,1	1,3	2,3	0,4	1,1	0,4	1,1	1,6	2,2	0,8	0,9	1,4	0,7	4,97
KB 27	*D. c.*	0,7	1,9	0,2	1,1	1,4	2,1	0,3	0,9	0,5	1,7	1,4	2,2	0,7	0,8	1,6	1,0	5,46
KB 28	*D. t.*	1,3	2,8	0,4	1,8	1,5	3,2	0,3	1,7	0,4	1,1	1,3	3,5	0,5	1,9	1,2	0,8	5,02
KB 29	*D. t.*	1,5	2,6	0,4	1,2	1,2	2,2	0,4	0,9	0,4	1,4	1,4	2,1	0,7	0,8	1,4	0,7	5,08
KB 30	*D. c.*	0,9	2,2	0,3	1,3	1,2	1,7	0,5	0,7	0,6	1,8	1,2	1,9	0,5	0,8	1,8	1,2	5,08
KB 4	*D. t.*	1,4	2,9	0,5	1,2	1,5	2,8	0,5	1,4	0,4	1,4	1,5	2,8	0,7	1,4	1,6	0,9	5,04
KB 5	*D. c.*	0,6	2,1	0,3	1,3	1,2	1,9	0,3	0,9	0,5	1,5	1,1	1,7	0,5	0,6	1,5	1,0	5,46
KB 6	*D. z.*	1,6	3,8	0,4	1,8	1,6	3,5	0,5	2,1	0,5	1,7	1,5	3,9	0,5	2,4	1,9	1,4	4,88
KB 7	*D. c.*	0,9	3,3	0,3	2,1	0,8	3,1	0,2	2,1	1,1	2,1	1,6	2,9	0,6	1,2	1,6	0,9	5,41
KB 8	*D. t.*	1,4	2,6	0,5	1,1	1,4	2,5	0,3	0,9	0,4	1,3	1,2	2,2	0,6	0,8	1,4	0,7	4,99
KB 9	*D. c.*	1,0	3,4	0,3	1,9	1,7	3,4	0,5	2,2	0,9	2,5	1,4	3,6	0,8	2,0	2,1	1,3	5,48
KLA 01	*D. c.*	1,0	2,0	0,3	1,3	1,5	2,1	0,7	1,1	0,4	1,6	1,2	2,2	0,8	0,9	1,7	1,9	5,66
KLA 02	*D. c.*	1,1	2,1	0,5	1,3	1,6	2,1	0,6	0,7	0,8	2,3	1,2	1,6	0,4	0,2	1,5	1,5	5,58
KLA 03	*D. c.*	0,9	2,2	0,4	1,2	1,4	1,9	0,7	1,0	0,6	1,9	1,2	2,0	0,4	1,1	1,6	1,5	5,57
KLA 04	*D. c.*	0,9	2,3	0,5	1,4	1,0	2,3	0,5	1,5	0,4	1,6	1,4	2,2	0,5	1,3	1,5	1,5	5,66
KLA 05	*D. c.*	1,0	3,1	0,4	2,1	1,5	3,3	0,9	1,8	0,6	2,0	1,5	2,7	0,9	1,6	1,8	2,1	5,67
KLA 06	*D. c.*	0,8	2,5	0,4	1,5	1,3	3,0	0,8	1,5	0,5	1,9	1,1	1,5	0,6	1,3	1,6	1,7	5,67
KLA 07	*D. c.*	0,6	1,7	0,4	1,7	1,1	2,2	0,6	1,2	0,5	2,1	1,3	2,2	0,5	1,0	1,8	1,9	5,53
KLA 08	*D. c.*	1,0	3,2	0,3	2,3	1,4	3,2	0,7	2,1	0,7	2,3	1,4	2,7	0,8	1,5	1,8	2,1	5,67
KLA 09	*D. c.*	1,1	3,0	0,4	1,9	1,4	3,1	0,7	1,9	0,5	1,8	1,1	2,4	0,7	1,3	1,6	1,7	5,71
KLA 10	*D. c.*	0,7	2,0	0,4	1,5	1,1	2,3	0,6	1,1	0,5	1,4	1,2	3,1	0,7	1,8	1,8	1,9	5,60
KLA1	*D. c.*	3,3	3,2	0,4	1,8	1,2	2,5	2,1	1,5	0,6	2,0	1,4	2,6	0,7	1,6	2,0	1,3	5,55
KLA2	*D. c.*	1,1	2,5	0,4	1,3	1,4	2,1	0,7	0,7	0,7	2,2	1,1	2,4	0,6	1,3	2,1	1,5	5,65
KLA3	*D. c.*	1,1	2,2	0,5	1,6	1,4	2,3	0,7	0,8	0,7	2,3	1,1	2,1	0,7	0,7	2,3	1,6	5,57
KLA4	*D. c.*	1,4	2,3	0,5	0,7	1,4	1,6	0,7	0,4	0,6	1,8	1,3	1,8	0,7	0,5	2,2	1,5	5,59
KLA5	*D. c.*	1,1	1,5	0,4	0,9	1,7	1,8	0,9	0,9	0,7	3,2	1,2	2,8	0,5	1,2	2,6	2,1	5,51
KNI 01	*D. o.*	2,1	2,2	0,5	-0,1	2,3	2,1	0,9	-0,1	0,4	2,6	1,9	1,9	0,7	-0,2	2,6	2,7	6,37
KNI 02	*D. a.*	1,9	1,7	0,5	-0,3	2,0	1,8	0,5	-0,1	0,9	2,3	1,9	1,6	0,7	-0,3	1,5	1,6	7,19
KNI 03	*D. t.*	1,3	1,9	0,3	0,7	1,5	2,2	0,4	1,1	0,3	0,9	1,4	1,9	0,7	1,0	1,3	1,5	5,31
KNI 04	*D. t.*	1,3	2,4	0,5	0,7	1,6	2,4	0,4	0,6	0,3	0,8	1,4	2,5	0,7	0,8	1,4	1,5	5,04
KNI 05	*D. t.*	1,5	2,7	0,5	1,0	1,7	2,4	0,5	0,7	0,4	1,1	1,4	2,6	0,6	1,4	1,3	1,4	5,01
KNI 06	*D. t.*	1,6	2,1	0,6	0,7	1,4	2,2	0,5	0,5	0,3	1,4	1,4	2,6	0,6	1,1	1,3	1,4	5,02
KNI 07	*D. a.*	1,7	1,8	0,7	-0,2	1,6	1,7	0,5	-0,1	0,8	2,3	1,9	1,6	0,8	-0,2	2,2	2,2	7,19
KNI 08	*D. a.*	2,5	1,9	0,8	-0,2	2,2	2,1	0,6	0,0	0,7	1,7	2,3	2,8	1,0	0,6	2,1	2,2	7,25
KNI 09	*D. a.*	1,7	1,6	0,6	-0,2	1,5	1,7	0,6	-0,1	0,6	2,0	1,7	1,5	0,6	-0,3	1,9	1,9	7,38
KNI 10	*D. a.*	2,3	2,2	1,0	-0,2	1,9	2,0	0,6	-0,3	0,8	2,3	2,2	2,3	1,0	-0,5	1,8	1,8	7,32
KR 1_10	*D. c.*	1,5	3,0	0,5	1,6	2,6	3,0	0,4	0,6	0,6	1,9	2,0	3,1	0,6	1,2	1,5	0,9	5,95
KR 1_11	*D. t.*	1,7	2,6	0,3	0,8	1,7	2,4	0,3	0,9	0,4	1,7	1,5	2,7	0,4	0,9	1,5	1,2	4,90

KR 1_12	*D. t.*	1,2	1,9	0,4	0,7	1,3	1,9	0,4	0,7	0,2	0,8	1,3	1,9	0,6	0,6	1,0	0,5	4,87
KR 1_13	*D. z.*	1,6	2,0	0,3	0,7	1,3	1,8	0,5	0,5	0,6	2,2	1,8	2,1	0,5	0,3	1,8	1,4	5,28
KR 1_14	*D. t.*	1,5	2,5	0,4	0,8	1,6	2,6	0,4	1,1	0,6	0,7	2,1	2,3	0,6	0,6	1,5	0,9	4,91
KR 1_15	*D. i.*	1,4	2,5	0,5	0,8	1,9	2,6	0,6	0,6	0,4	2,1	1,6	2,2	0,5	0,7	1,6	1,1	6,04
KR 1_16	*D. t.*	1,1	2,1	0,6	1,1	1,4	2,0	0,3	0,6	0,3	1,5	1,1	2,5	0,6	0,8	1,4	0,7	4,87
KR 1_17	*D. i.*	2,0	2,8	0,4	1,0	1,7	3,0	0,4	0,7	0,7	1,9	1,9	2,5	0,8	0,9	1,8	1,0	5,97
KR 1_18	*D. a.*	2,6	3,4	0,8	0,8	2,4	3,6	0,6	1,3	0,8	2,4	2,4	3,9	0,6	1,3	1,6	1,0	7,05
KR 1_19	*D. t.*	1,5	2,5	0,3	1,2	1,5	2,8	0,3	1,0	0,4	1,5	1,3	2,3	0,5	0,9	1,5	1,0	4,84
KR 1_2	*D. c.*	1,6	4,3	0,5	2,6	1,6	4,0	0,6	2,7	0,9	2,9	1,5	3,5	0,9	1,9	2,2	1,3	5,41
KR 1_20	*D. t.*	1,9	1,9	0,3	0,4	1,8	2,6	0,4	0,7	0,3	1,1	1,8	3,1	0,4	0,8	1,5	1,1	4,89
KR 1_21	*D. a.*	2,3	2,9	0,6	0,3	1,6	2,7	0,6	1,0	0,9	2,1	1,2	2,4	0,7	0,8	1,2	0,5	7,12
KR 1_22	*D. i.*	1,9	1,3	0,4	0,1	1,7	1,9	0,4	0,3	0,3	1,8	1,9	2,0	0,4	0,2	0,9	0,5	6,16
KR 1_23	*D. i.*	2,3	4,0	0,4	1,3	2,2	4,1	0,7	1,7	0,7	1,2	1,9	3,8	0,5	1,4	1,3	0,9	5,95
KR 1_24	*D. i.*	2,0	2,4	0,8	0,4	2,1	2,5	0,7	0,5	0,7	2,2	2,2	2,8	1,0	0,8	2,0	1,0	6,07
KR 1_25	*D. t.*	1,2	2,7	0,4	0,9	1,3	2,5	0,3	1,1	0,6	1,2	1,4	2,7	0,4	0,7	1,5	1,1	5,05
KR 1_29	*D. c.*	1,4	2,0	0,4	0,5	1,7	1,9	0,6	0,5	0,7	1,9	1,3	2,2	0,8	0,5	2,2	1,4	5,51
KR 1_3	*D. i.*	1,2	2,0	0,4	0,7	1,6	2,4	0,5	0,4	0,5	1,6	1,8	2,2	0,8	0,4	1,7	0,9	6,00
KR 1_31	*D. c.*	1,4	2,5	0,5	1,1	1,8	2,5	0,6	0,8	0,6	2,3	1,5	1,9	0,6	0,3	1,9	1,3	5,53
KR 1_4	*D. t.*	1,3	3,7	0,4	1,9	1,7	3,2	0,6	1,6	0,9	2,2	1,9	3,4	0,8	1,1	2,3	1,5	5,28
KR 1_6	*D. o.*	1,6	1,8	0,5	0,2	1,5	1,6	0,7	0,0	0,5	2,1	1,6	1,6	0,8	0,3	2,0	1,3	6,30
KR 1_7	*D. i.*	1,9	3,0	0,5	1,0	2,0	2,9	0,5	0,9	0,6	1,4	1,8	2,7	0,8	0,7	1,6	0,9	5,91
KR 1_8	*D. c.*	1,0	2,3	0,3	0,9	1,7	2,1	0,5	0,4	0,5	2,2	1,4	2,6	0,6	0,9	1,8	1,1	5,30
KR 1_9	*D. o.*	1,7	3,0	0,7	0,6	2,5	3,0	0,9	0,4	0,7	2,5	1,6	2,5	0,8	0,5	2,7	1,9	6,24
KR 2_1	*D. c.*	1,0	3,2	0,5	1,8	2,0	3,2	0,6	1,5	0,5	2,7	1,7	3,1	0,8	1,8	2,2	1,5	5,41
KR 2_3	*D. c.*	1,4	2,0	0,3	0,5	1,6	1,5	0,3	0,4	0,6	1,5	1,5	1,8	0,4	0,3	1,4	1,0	5,48
KR 2B_1	*D. c.*	0,9	1,4	0,2	0,6	1,4	1,8	0,5	0,6	0,4	1,3	1,5	1,9	0,5	0,1	1,5	0,9	5,52
KR 2B_2	*D. c.*	0,8	1,9	0,3	0,5	1,5	1,8	0,2	0,4	0,4	1,2	1,4	1,5	0,4	0,6	1,2	0,8	5,52
KR 2B_3	*D. c.*	1,3	1,5	0,5	0,6	1,5	2,1	0,4	0,9	0,8	1,9	1,5	1,9	0,6	0,2	2,2	1,5	5,58
KR 3_1	*D. i.*	1,7	1,8	0,5	0,4	1,7	1,9	0,7	0,6	0,5	2,1	1,8	1,7	0,7	-0,1	2,1	1,4	6,31
KR 3_2	*D. i.*	1,4	2,3	0,6	0,4	1,9	2,4	0,6	0,4	0,8	2,1	2,0	2,2	0,7	0,5	1,9	1,2	6,12
KR 4_1	*D. t.*	1,4	1,8	0,3	0,9	1,1	1,9	0,3	0,6	0,3	0,9	1,3	2,1	0,4	0,4	0,9	0,5	4,98
KR 4_2	*D. a.*	1,9	1,5	0,8	-0,4	1,8	1,5	0,5	0,2	0,6	2,2	2,1	1,4	0,7	0,4	1,1	0,4	7,18
KR 4_3	*D. a.*	1,7	1,6	0,5	-0,2	1,8	1,7	0,5	-0,1	0,5	1,8	1,5	1,6	0,5	-0,1	1,6	1,1	7,17
KRK 1_1	*D. i.*	2,0	3,1	0,6	0,6	2,0	2,8	0,7	0,7	0,5	1,8	1,8	3,1	0,7	0,9	2,0	1,3	5,94
KRK 1_2	*D. t.*	1,5	2,0	0,6	0,7	1,6	1,7	0,6	0,2	0,4	1,0	1,3	2,1	0,6	0,9	1,4	0,7	4,99
KRK 1_3	*D. i.*	2,2	2,8	0,8	0,6	2,1	2,7	0,6	0,5	0,6	2,0	1,9	2,5	0,7	0,7	0,8	0,1	6,07
KRK 3	*D. a.*	2,1	2,5	0,8	0,3	2,1	2,1	0,5	0,4	0,4	1,5	2,0	2,5	0,7	0,7	2,0	1,2	7,08
KTI 01	*D. c.*	0,5	2,6	0,3	1,7	1,9	2,8	0,9	1,6	0,8	3,5	1,6	2,6	0,4	1,5	2,2	2,2	5,39
KTI 02	*D. c.*	1,1	3,2	0,2	2,1	1,6	3,6	1,1	2,0	0,8	4,1	1,1	3,1	0,5	1,5	1,7	1,8	5,24
KTI 03	*D. c.*	1,5	3,1	0,4	1,3	1,8	3,7	0,9	1,8	1,0	2,2	1,4	2,2	0,5	0,9	1,8	1,9	5,28
KUB 01	*D. t.*	1,5	2,0	0,5	0,6	1,6	1,9	0,4	0,7	0,3	1,3	1,4	1,9	0,7	0,7	1,4	1,5	4,89
KUB 02	*D. z.*	2,1	2,8	0,4	0,7	2,0	2,7	0,6	1,0	0,7	1,3	1,1	3,4	0,6	0,9	1,6	1,7	5,16
KUB 03	*D. a.*	1,6	1,7	0,5	-0,2	2,0	1,8	0,7	0,1	0,8	2,1	2,0	1,6	0,8	-0,3	2,3	2,4	7,19
KUB 04	*D. a.*	2,3	3,1	0,6	0,7	2,0	3,1	0,6	1,1	0,6	2,0	1,8	3,0	0,6	0,8	1,4	1,4	7,33
KUB 05	*D. i.*	1,6	2,6	0,4	0,7	1,6	2,4	0,8	0,4	0,6	1,9	1,5	2,1	0,6	0,7	1,9	2,0	6,42
KUB 06	*D. a.*	2,5	3,2	0,6	0,5	2,7	3,0	0,8	0,7	0,7	2,8	2,0	3,0	0,8	1,3	1,9	2,0	7,16
KUB 07	*D. o.*	1,8	2,5	0,8	0,9	2,0	2,6	0,9	0,7	0,7	2,5	1,8	2,6	0,8	1,0	2,6	2,6	6,37
KUB 08_1	*D. z.*	1,1	2,1	0,3	0,9	1,3	1,9	0,7	0,4	0,5	1,5	1,1	2,0	0,5	0,9	1,8	1,9	4,94
KUB 08_2	*D. t.*	1,9	2,0	0,4	0,4	1,4	1,8	0,5	0,1	0,4	0,8	1,6	2,2	0,5	0,2	1,3	1,3	5,40
KUB 08_3	*D. c.*	1,9	2,3	0,4	0,4	1,9	2,2	0,5	0,5	0,4	2,0	1,9	1,9	0,4	0,3	1,9	2,0	5,48

KUB 09	*D. c.*	1,9	2,2	0,4	2,4	2,0	2,4	0,5	0,7	0,7	2,0	1,8	3,1	0,5	0,6	1,5	1,6	5,48
KUB 10	*D. a.*	2,6	3,5	0,8	1,3	3,0	3,7	1,0	1,5	1,3	3,2	2,1	4,3	1,0	2,1	2,0	2,2	7,14
KUB 11	*D. a.*	1,9	2,2	0,8	0,1	1,9	2,0	0,8	0,3	0,9	2,3	2,0	2,1	0,8	0,0	1,4	1,4	7,23
KUB 12	*D. a.*	1,5	1,5	0,7	-0,3	1,8	1,6	0,5	-0,3	0,8	2,0	2,0	1,7	0,8	-0,4	1,4	1,5	7,27
KUB 13	*D. o.*	1,8	2,3	0,5	1,3	2,0	2,2	0,8	0,4	0,7	2,7	2,0	1,9	0,6	-0,3	1,9	1,9	6,33
KUB 14A	*D. o.*	2,0	2,7	0,4	0,8	1,9	2,5	0,7	0,6	0,7	1,8	1,8	2,1	0,5	0,4	1,6	1,7	6,30
KUB 14B	*D. o.*	1,7	2,0	0,5	0,2	1,7	1,9	0,6	0,1	0,6	2,1	1,4	1,9	0,7	0,4	2,0	2,1	6,29
KUB 15	*D. z.*	1,3	1,9	0,3	0,5	1,5	1,9	0,5	0,4	0,5	0,6	1,4	1,8	0,6	0,5	1,3	1,4	5,29
KUB 16A	*D. i.*	1,6	2,4	0,4	0,6	1,8	2,5	0,9	0,6	0,6	1,5	2,0	3,0	0,8	1,0	2,4	2,6	6,42
KVIL1	*D. a.*	1,5	1,3	0,6	-0,2	1,7	1,2	0,7	-0,3	0,6	2,0	1,5	1,4	0,6	-0,3	2,0	1,4	7,45
LAK 01	*D. a.*	2,0	1,9	0,9	0,2	2,0	2,2	0,6	0,1	0,5	2,3	2,2	1,7	0,9	-0,6	1,0	1,0	7,19
LAK 02	*D. a.*	2,4	2,4	0,8	0,1	2,4	2,6	0,7	-0,2	0,5	2,2	2,3	2,1	0,8	0,3	0,7	0,7	7,28
LAK 03	*D. a.*	2,8	3,7	0,6	1,0	2,5	3,9	1,0	1,7	0,6	3,1	3,1	3,8	0,8	1,4	0,9	1,1	6,90
LAK 04	*D. i.*	1,7	3,4	0,5	1,8	2,0	3,4	1,0	1,4	0,7	2,4	2,1	3,8	0,9	1,3	1,9	2,2	6,37
LAK 05	*D. i.*	1,5	4,9	0,4	3,2	2,5	4,6	1,0	3,0	0,7	3,7	1,7	4,9	0,6	2,8	1,0	1,1	6,36
LAK 06	*D. a.*	2,2	2,9	0,9	0,5	2,6	2,7	0,7	0,2	0,7	2,6	2,5	2,9	0,9	0,6	1,5	1,5	7,09
MH1	*D. o.*	1,7	1,9	0,4	0,3	1,6	2,0	0,6	0,3	0,7	2,1	1,4	1,8	0,6	0,5	2,0	2,1	6,42
MH10	*D. o.*	1,5	0,6	0,4	0,8	1,6	1,9	0,6	0,5	0,7	2,4	1,7	2,1	0,6	0,8	2,0	2,1	6,39
MH2	*D. t.*	1,0	2,0	0,3	1,0	1,1	1,9	0,5	0,8	0,4	1,2	1,2	2,3	0,5	0,9	1,7	1,8	5,50
MH3	*D. o.*	2,2	3,1	0,6	1,0	2,6	3,4	0,9	1,0	0,6	2,5	1,9	3,6	0,8	1,8	2,3	2,5	6,51
MH4	*D. o.*	2,2	2,7	0,4	0,6	1,4	2,3	0,6	0,6	0,6	2,2	1,9	2,8	0,9	1,0	2,0	2,2	6,43
MH5	*D. a.*	2,1	3,2	0,6	0,9	2,2	3,3	0,8	1,2	0,9	2,8	1,8	2,5	0,8	0,7	1,6	1,7	7,32
MH6	*D. o.*	1,5	2,4	0,5	0,9	2,1	2,2	0,7	0,5	0,6	2,1	1,5	2,5	0,8	0,5	2,4	2,5	6,49
MH7	*D. o.*	1,2	2,5	0,5	0,8	1,9	2,7	0,8	0,5	0,7	2,4	1,6	2,8	0,7	0,9	2,2	2,3	6,37
MH8	*D. o.*	1,7	1,9	0,5	0,7	1,5	2,1	0,7	0,6	0,6	2,5	1,5	2,2	0,7	0,8	1,9	2,1	6,46
MH9	*D. o.*	1,4	1,8	0,5	0,1	1,9	1,9	0,6	0,3	0,5	2,5	1,4	1,9	0,9	0,2	2,2	2,4	6,36
OLS 1	*D. a.*	2,8	4,4	0,9	1,2	2,5	3,8	1,0	1,4	0,9	3,7	2,5	4,2	0,8	1,9	1,2	1,1	7,32
OLS 10	*D. a.*	2,5	2,4	0,5	0,0	2,4	2,1	0,9	-0,3	0,8	3,2	2,1	2,3	0,9	-0,2	1,6	1,8	7,31
OLS 11	*D. o.*	1,3	2,3	0,5	1,1	1,7	2,4	0,9	0,7	0,5	2,5	1,9	2,3	0,9	0,5	2,3	2,5	6,34
OLS 12	*D. i.*	2,2	3,5	0,7	1,2	2,0	3,1	1,2	1,2	0,7	2,6	2,8	2,9	0,8	0,8	2,6	2,7	6,44
OLS 13	*D. o.*	1,5	2,4	0,5	0,6	1,7	2,4	0,9	1,1	0,7	2,4	1,9	2,4	0,7	0,7	2,6	2,7	6,38
OLS 14	*D. a.*	2,1	2,5	0,8	0,0	2,3	2,2	0,7	-0,1	0,7	3,4	2,4	2,5	0,9	-0,2	1,3	1,3	7,32
OLS 15	*D. a.*	1,6	1,9	0,7	-0,2	2,6	2,1	0,5	-0,4	0,7	3,0	2,1	2,0	0,8	-0,3	2,0	2,1	7,41
OLS 16	*D. a.*	2,1	1,7	0,7	-0,1	1,8	1,7	0,4	-0,2	0,8	2,2	2,0	1,7	0,8	-0,3	2,1	2,2	7,35
OLS 17	*D. a.*	1,9	2,0	0,7	1,5	2,2	2,1	0,9	-0,2	0,9	2,3	2,0	2,1	0,9	-0,3	2,4	2,5	7,40
OLS 18	*D. a.*	2,2	2,8	0,7	0,4	1,9	2,6	0,7	0,7	0,8	2,1	1,9	2,3	0,7	1,4	1,7	1,7	7,35
OLS 19	*D. a.*	2,5	2,5	0,8	0,1	2,0	2,2	0,8	0,5	0,8	2,9	1,8	2,6	0,8	0,6	1,6	1,6	7,39
OLS 2	*D. a.*	2,2	2,6	0,6	0,3	2,3	2,3	1,0	0,2	0,7	2,6	1,6	2,8	0,7	0,8	1,3	1,4	7,39
OLS 3	*D. a.*	2,2	2,7	0,6	0,3	2,2	2,8	0,8	0,2	0,8	2,7	1,8	2,8	0,8	0,3	1,4	1,5	7,20
OLS 4	*D. a.*	2,3	2,8	0,7	0,5	2,3	2,2	0,9	0,4	0,5	2,3	2,2	2,7	0,7	0,6	1,6	1,6	6,84
OLS 5	*D. a.*	2,0	2,4	0,6	0,1	2,6	2,5	0,8	0,1	0,9	2,7	1,9	2,1	0,8	-0,1	1,5	1,6	7,32
OLS 6	*D. a.*	2,5	2,4	0,7	0,0	2,3	2,2	0,8	0,1	0,9	2,9	2,1	2,2	0,9	-0,2	2,0	2,1	7,28
OLS 7	*D. a.*	2,3	2,8	0,8	0,4	2,3	2,8	0,9	0,6	0,7	2,9	2,2	2,6	0,8	0,6	1,1	1,1	7,11
OLS 8	*D. a.*	2,1	2,1	0,6	-0,1	2,3	2,3	0,7	0,3	0,7	2,8	2,0	2,2	0,8	0,3	1,3	1,4	7,25
OLS 9	*D. a.*	1,6	1,6	0,7	0,0	2,0	2,0	0,6	0,0	0,7	2,7	1,9	2,0	0,6	0,0	1,1	1,0	7,25
OST 02	*D. z.*	1,1	2,6	0,4	1,6	1,5	2,6	0,6	1,1	0,5	1,4	1,2	2,7	0,5	1,6	1,8	1,8	5,28
OST 03	*D. z.*	1,5	2,0	0,5	0,6	1,6	2,1	0,6	0,4	0,4	1,3	1,6	2,4	0,9	0,4	1,6	1,7	5,28
OST 04	*D. z.*	1,4	3,2	0,6	1,7	1,4	2,8	0,7	1,3	0,5	1,5	1,5	2,9	0,9	1,8	1,8	2,0	5,32
OST 05	*D. z.*	1,5	2,8	0,4	1,4	1,6	2,7	0,7	1,1	0,6	0,9	1,5	2,5	0,9	1,4	1,7	1,9	5,30
OST 06	*D. z.*	1,6	2,9	0,4	1,0	2,2	2,6	0,7	0,9	0,4	1,2	1,9	2,2	0,7	0,7	1,8	2,0	5,20

POL 1	*D. t.*	1,6	2,8	0,5	1,0	1,6	3,1	0,5	1,0	0,3	1,2	1,5	3,2	0,8	1,5	1,5	1,7	5,06
POL 2	*D. t.*	1,7	2,2	0,5	0,5	1,6	2,4	0,5	0,6	0,3	1,4	1,5	2,4	0,8	0,4	1,5	1,6	4,98
POL 4	*D. t.*	1,5	1,9	0,5	0,4	1,5	1,8	0,5	0,1	0,4	1,4	1,3	2,1	0,8	0,6	1,5	1,6	4,99
POL 5	*D. t.*	1,6	3,4	0,6	1,2	1,8	3,4	0,7	0,9	0,4	1,5	1,7	3,4	0,8	1,2	1,8	1,9	4,92
POL 6	*D. t.*	1,8	2,6	0,5	0,8	1,8	2,5	0,5	0,8	0,4	1,5	1,3	2,6	0,7	1,1	1,5	1,6	4,97
POL 7	*D. z.*	1,9	4,4	0,5	2,8	2,2	4,6	0,6	2,5	0,6	1,6	1,1	3,3	0,5	1,4	1,6	1,6	5,22
POL 8	*D. t.*	1,5	2,4	0,6	0,8	1,3	2,3	0,5	1,1	0,3	1,0	1,6	2,3	0,8	0,7	1,4	1,5	4,97
POL 9	*D. t.*	1,7	2,5	0,4	0,6	1,6	2,2	0,4	0,5	0,4	1,3	1,5	2,3	0,6	0,7	1,3	1,4	4,98
POL3	*D. t.*	1,7	3,2	0,4	1,4	1,8	3,2	0,5	1,3	0,4	0,9	1,5	2,8	0,6	1,0	1,4	1,5	5,07
PRI 01	*D. c.*	0,6	2,4	0,3	1,6	1,5	2,2	0,8	1,5	0,8	2,9	1,0	2,4	0,5	1,4	2,0	2,1	5,52
PRI 02	*D. c.*	1,0	3,1	0,4	2,5	1,5	2,9	0,9	1,7	0,9	3,3	1,1	2,5	0,7	1,8	2,3	2,5	5,59
PRI 03	*D. c.*	0,6	3,0	0,3	2,0	1,9	2,6	0,7	1,8	0,6	3,5	1,0	2,6	0,3	1,4	1,6	1,7	5,56
PRI 04	*D. c.*	1,1	4,5	0,4	3,3	1,7	4,0	0,8	2,5	1,2	3,5	1,6	3,5	0,3	2,2	2,7	2,7	5,47
PRI 05	*D. c.*	1,0	3,1	0,3	2,2	2,0	2,9	0,6	1,5	0,8	3,4	1,4	3,4	0,4	1,8	2,0	2,1	5,46
PRIL 01	*D. a.*	2,4	2,5	0,6	0,2	2,0	1,9	0,8	0,0	0,5	2,4	2,1	2,9	0,8	0,8	1,5	1,6	7,21
PRIL 02	*D. a.*	2,7	3,8	1,0	1,4	2,7	3,8	0,8	1,3	0,8	2,9	2,3	4,0	0,8	2,3	1,8	1,7	7,20
PRIL 03	*D. c.*	0,8	3,0	0,3	2,0	1,8	3,4	0,8	1,9	0,8	2,6	1,2	3,1	0,6	1,9	2,0	2,2	5,44
PRIL 04	*D. a.*	2,1	3,3	0,6	1,1	2,5	3,4	0,8	1,1	0,8	3,5	2,3	3,5	0,7	1,0	1,8	1,9	7,25
PRIL 05	*D. a.*	2,5	2,7	0,7	0,4	2,5	2,3	0,7	-0,2	0,8	2,6	2,0	2,4	0,7	0,4	1,6	1,6	7,27
PRIL 06	*D. i.*	1,6	3,5	0,5	1,7	2,0	3,4	1,0	1,2	0,7	2,8	1,8	3,5	0,6	1,3	2,3	2,3	6,23
PRIL 07	*D. c.*	1,1	2,7	0,3	1,9	1,9	3,1	0,7	1,3	0,9	3,0	1,6	3,1	0,6	1,7	2,0	2,2	5,61
PRIL1	*D. a.*	2,6	3,9	0,9	1,4	2,7	4,3	0,9	1,5	0,8	2,5	2,2	3,9	0,9	1,5	2,7	1,8	7,22
PRIL2	*D. c.*	0,9	3,5	0,4	2,6	1,7	3,7	1,0	2,7	0,7	3,6	1,1	3,7	0,5	2,4	2,7	2,2	5,54
PRIL3	*D. c.*	1,5	3,3	0,4	2,0	2,3	2,9	0,9	1,6	0,8	3,1	1,2	2,8	0,5	1,4	2,5	2,0	5,53
PRIL4	*D. c.*	0,9	2,1	0,5	1,3	1,4	2,4	0,6	1,0	0,7	1,9	1,4	1,9	0,5	0,6	2,1	1,6	5,56
R1	*D. c.*	1,3	2,3	0,3	0,7	1,9	2,1	0,6	0,3	0,8	2,2	1,4	2,4	0,3	0,6	1,8	1,5	5,51
R10	*D. i.*	2,0	2,6	0,7	0,5	2,0	2,4	0,7	0,5	0,6	2,0	1,5	2,3	0,9	0,6	2,2	1,3	6,41
R11	*D. c.*	0,7	1,8	0,3	1,3	1,5	1,8	0,6	0,7	0,4	2,0	1,4	2,2	0,5	1,1	2,0	1,5	5,51
R12	*D. c.*	1,2	2,2	0,3	0,6	1,7	2,3	0,4	0,6	0,8	2,1	1,5	2,8	0,7	0,5	1,8	1,1	5,47
R13	*D. t.*	1,3	1,7	0,5	0,4	1,4	1,6	0,5	0,4	0,3	1,5	1,0	1,3	0,6	0,4	1,5	0,8	5,22
R14	*D. t.*	1,1	1,5	0,3	0,4	1,2	1,4	0,4	0,1	0,4	1,0	1,2	1,7	0,4	0,3	1,3	0,8	5,21
R15	*D. c.*	0,9	2,4	0,3	1,4	1,6	2,5	0,8	0,8	0,6	2,3	1,1	2,1	0,5	0,9	1,9	1,4	5,52
R17	*D. c.*	1,4	1,5	0,5	0,5	1,3	1,5	0,6	0,4	0,5	1,7	1,3	1,6	0,4	0,4	2,3	1,9	5,00
R18	*D. t.*	1,2	1,6	0,4	0,4	1,2	1,4	0,3	0,3	0,2	1,2	1,1	1,5	0,7	0,2	1,3	0,6	5,10
R19	*D. i.*	1,9	2,1	0,3	0,3	2,0	1,6	0,9	0,2	0,3	1,2	1,3	1,5	0,5	0,4	2,1	1,6	6,37
R2	*D. c.*	1,7	2,4	0,7	2,5	1,6	2,3	0,6	0,6	0,4	1,8	1,6	2,3	0,7	0,9	1,7	1,1	5,29
R20	*D. c.*	1,7	2,7	0,5	1,0	1,8	2,4	0,5	0,7	0,5	1,6	1,7	2,7	0,9	0,6	1,8	0,9	5,43
R21	*D. t.*	1,1	2,2	0,5	1,0	1,5	2,6	0,5	0,6	0,5	1,8	1,4	2,2	0,7	0,6	1,5	0,8	4,95
R23	*D. i.*	1,5	3,0	0,5	1,5	1,6	3,4	0,6	1,6	0,9	2,5	1,4	3,5	0,9	1,8	1,7	0,9	6,20
R24	*D. c.*	1,1	1,9	0,3	0,6	1,3	1,4	0,5	0,2	0,5	1,6	1,2	1,7	0,4	0,5	1,7	1,3	5,37
R26	*D. a.*	1,5	1,3	0,7	-0,4	1,4	1,1	0,3	-0,4	0,7	1,7	1,0	1,1	0,7	0,2	1,8	1,2	7,31
R27	*D. z.*	1,6	3,0	0,5	1,8	1,7	3,7	0,7	2,1	0,6	2,0	1,4	2,9	0,7	1,9	1,9	1,3	5,10
R28	*D. c.*	0,6	2,4	0,3	1,4	1,5	2,2	0,6	1,2	0,5	2,5	0,9	1,9	0,4	0,7	1,5	1,0	5,01
R29	*D. c.*	1,4	2,3	0,3	1,1	1,7	2,0	0,4	0,4	0,8	2,1	1,5	1,6	0,3	0,8	1,7	1,4	5,54
R3	*D. c.*	0,8	1,6	0,4	0,4	1,4	1,2	0,6	0,5	0,3	1,9	1,4	1,9	0,5	0,3	1,8	1,3	5,48
R30	*D. c.*	1,1	2,8	0,5	2,1	1,6	2,4	0,8	1,0	0,6	2,1	1,5	2,7	0,3	1,2	1,9	1,5	5,53
R31	*D. a.*	2,1	3,0	0,6	0,4	2,4	2,6	0,8	0,7	0,5	2,8	1,8	2,4	0,8	1,0	1,5	0,7	7,24
R32	*D. c.*	0,5	2,1	0,4	1,9	1,3	1,8	0,5	0,7	0,5	1,8	1,3	2,0	0,4	0,5	1,8	1,3	5,41
R33	*D. c.*	1,3	2,6	0,4	1,3	1,7	1,9	0,5	1,7	0,5	1,9	1,6	2,1	1,8	0,3	1,7	-0,1	5,44
R34	*D. c.*	0,9	1,9	0,3	0,9	1,4	1,9	0,5	0,5	0,6	2,2	1,5	1,8	0,6	0,7	1,5	0,9	5,01

R35	*D. o.*	1,3	2,8	0,3	1,6	1,7	2,9	0,9	1,3	0,5	2,4	1,3	2,9	0,6	1,0	0,9	0,3	6,30
R36	*D. z.*	1,1	1,8	0,5	0,6	1,3	1,9	0,5	0,6	0,3	1,2	0,7	1,7	0,6	0,8	1,4	0,8	5,44
R37	*D. t.*	1,4	0,7	0,4	0,8	1,1	2,1	0,4	0,8	0,4	1,3	1,0	1,8	0,7	0,6	1,5	0,8	5,00
R38	*D. z.*	1,1	1,7	0,4	0,6	1,3	1,5	0,4	0,4	0,3	1,3	1,2	1,2	0,6	0,5	1,4	0,8	5,28
R39	*D. i.*	1,6	1,7	0,6	0,3	1,7	1,5	0,7	0,0	0,5	2,0	1,5	1,6	0,7	0,3	1,9	1,2	6,43
R4	*D. c.*	1,3	2,4	0,5	0,9	1,4	2,3	0,7	0,7	0,5	1,7	1,4	2,0	0,7	0,9	1,6	1,0	5,37
R40	*D. c.*	1,3	2,7	0,4	1,6	2,1	2,3	0,7	0,8	0,9	2,7	1,4	2,8	0,7	0,6	2,5	1,8	5,58
R41	*D. a.*	1,9	2,3	0,6	0,6	2,0	2,2	0,9	0,4	0,6	2,5	1,2	2,0	0,8	0,4	2,1	1,3	7,39
R42	*D. t.*	1,3	1,3	0,3	0,3	1,2	1,4	0,3	0,4	0,4	1,3	0,7	1,7	0,7	0,9	1,3	0,6	5,01
R43	*D. z.*	1,1	1,6	0,3	0,4	1,3	1,6	0,4	0,5	0,4	1,3	1,3	1,5	0,3	0,5	1,2	0,9	5,36
R44	*D. c.*	0,8	1,5	0,3	0,7	1,3	1,7	0,7	0,3	0,4	1,6	1,1	1,6	0,4	0,4	1,8	1,3	5,64
R45	*D. z.*	1,3	2,1	0,6	0,5	1,5	1,9	0,5	0,6	0,6	1,8	1,9	2,2	0,6	0,6	0,7	0,1	5,26
R5	*D. t.*	1,1	1,7	0,3	0,5	1,6	2,0	0,5	0,2	0,4	1,4	1,5	2,0	0,6	0,2	1,4	0,8	4,96
R6	*D. t.*	1,5	2,0	0,5	0,3	1,5	1,9	0,5	0,7	0,3	1,8	1,3	1,8	0,6	0,4	1,8	1,2	5,22
R7	*D. c.*	0,9	1,6	0,3	0,5	1,4	1,3	0,5	0,2	0,4	1,6	1,2	1,4	0,4	0,3	1,8	1,4	5,39
R8	*D. c.*	0,9	1,9	0,3	0,7	1,2	2,0	0,8	0,7	0,3	1,9	1,1	1,9	0,7	0,6	2,1	1,3	5,62
R9	*D. a.*	0,9	1,6	0,6	0,3	1,3	1,7	0,3	-0,3	1,1	2,4	1,5	1,3	0,5	0,3	1,9	1,3	7,28
RAK	*D. a.*	2,5	2,2	0,8	-0,1	2,2	2,1	0,7	0,0	0,5	2,5	1,9	2,3	0,9	0,4	2,4	1,5	7,54
RAMZ01	*D. o.*	2,0	3,0	0,5	1,0	2,3	2,5	0,7	0,3	0,6	1,7	1,8	2,5	0,7	0,8	1,9	2,1	6,32
RAMZ02	*D. t.*	1,5	2,4	0,4	0,7	1,5	2,2	0,4	0,6	0,4	1,1	1,4	2,4	0,5	0,9	1,3	1,3	5,07
RAMZ03	*D. c.*	1,4	3,7	0,6	2,5	1,3	3,4	0,6	2,0	0,5	1,9	1,2	2,6	0,5	1,3	2,0	1,9	5,53
RAMZ04	*D. a.*	2,0	1,6	0,7	-0,3	2,0	1,5	0,6	-0,3	0,7	2,2	1,7	1,6	0,8	-0,5	2,0	2,0	7,44
RAMZ06	*D. c.*	1,5	3,5	0,5	1,9	1,5	3,0	0,7	1,6	0,7	2,1	1,5	2,9	0,7	1,4	2,2	2,3	5,47
RAMZ07	*D. c.*	1,1	4,1	0,3	3,0	1,6	3,5	0,9	2,0	0,8	2,4	1,5	3,8	0,4	2,1	2,3	2,3	5,70
RAMZ08	*D. t.*	1,6	1,7	0,4	0,1	1,7	1,9	0,4	0,1	0,4	1,2	1,5	1,7	0,6	0,2	1,5	1,6	4,98
RAMZ09	*D. z.*	1,6	2,4	0,6	0,9	1,7	2,4	0,7	0,8	0,6	1,9	1,7	2,6	0,8	0,6	1,8	2,0	5,30
RAMZ10	*D. i.*	2,2	2,7	0,8	0,5	2,3	2,2	0,8	0,6	0,5	2,1	2,0	2,6	0,8	0,6	2,0	2,0	6,41
RAMZ11	*D. i.*	2,1	2,5	0,7	0,6	2,3	2,8	0,8	0,4	0,6	2,3	1,9	2,1	0,8	0,9	2,2	2,2	6,41
RAMZ13	*D. t.*	1,5	2,4	0,6	0,9	1,8	2,0	0,3	0,5	0,3	0,9	1,4	2,5	0,7	0,9	1,1	1,2	4,98
RYŽ 1	*D. c.*	1,5	3,6	0,6	2,1	2,0	3,3	0,9	1,7	0,8	1,9	1,6	3,1	0,8	2,0	2,0	1,3	5,42
RYŽ 2	*D. a.*	1,8	2,3	0,4	0,4	1,8	2,2	0,7	0,5	0,5	1,8	2,0	2,4	0,7	0,4	2,0	1,2	7,12
RYŽ 3	*D. a.*	1,8	2,1	0,5	0,4	1,8	1,9	0,7	0,2	0,5	1,9	1,5	2,0	0,6	0,4	0,7	0,1	7,20
SRN	*D. i.*	2,5	4,0	0,7	1,3	2,3	3,5	0,9	1,5	0,8	2,3	2,1	3,9	0,9	1,9	2,8	1,9	6,12
SRN 01	*D. i.*	2,2	2,9	0,4	0,9	2,1	2,8	0,8	1,0	0,5	2,2	2,0	2,6	0,6	0,6	2,3	2,4	6,08
SRN 02	*D. i.*	2,4	3,0	0,5	0,7	2,2	3,0	0,8	0,8	0,5	1,2	2,0	3,2	0,8	0,9	2,5	2,6	6,08
STA 1	*D. o.*	1,9	2,5	0,5	0,5	2,1	2,3	0,7	0,3	0,7	1,8	1,8	2,0	0,6	0,4	2,3	1,7	6,40
STA 2	*D. o.*	2,2	2,5	0,7	0,4	2,4	3,1	1,0	0,3	0,6	2,2	1,8	2,3	0,8	0,8	2,2	1,4	6,49
Š1	*D. a.*	1,8	2,0	0,4	0,1	1,9	2,2	0,6	0,4	0,5	1,7	1,6	1,9	0,6	0,4	1,4	0,8	7,09
Š10	*D. a.*	2,3	1,6	0,6	-0,7	1,7	1,6	0,6	-0,5	0,8	2,2	1,9	1,4	0,9	-0,6	2,3	1,4	7,28
Š11	*D. a.*	2,1	2,1	0,8	-0,4	2,2	1,9	0,5	-0,1	1,1	2,3	2,2	2,2	0,7	0,4	2,1	1,5	7,28
Š2	*D. i.*	1,6	2,8	0,6	1,4	1,9	3,0	0,8	1,1	0,7	2,3	1,8	3,1	0,9	1,2	2,0	1,2	6,37
Š4	*D. a.*	2,2	2,7	0,8	0,2	2,3	2,1	0,8	0,4	0,7	2,5	1,9	2,4	0,8	0,2	2,5	1,7	7,30
Š5	*D. a.*	1,9	2,8	0,5	0,5	2,1	3,0	0,6	1,1	0,4	1,8	2,3	1,9	0,7	-0,2	1,8	1,2	7,19
Š6	*D. a.*	2,1	2,2	0,7	0,2	2,1	2,4	0,7	0,5	0,6	2,3	1,8	1,8	0,7	0,4	1,1	0,4	7,31
Š7	*D. i.*	1,8	1,9	0,5	-0,2	1,6	1,7	0,6	0,2	0,5	1,5	1,5	1,7	0,7	0,3	1,7	1,0	6,34
Š8	*D. a.*	2,0	1,5	0,7	-0,5	2,1	1,7	0,6	-0,5	0,8	2,1	1,4	1,4	0,8	0,4	1,8	1,0	6,90
Š8	*D. a.*	1,7	1,4	0,4	-0,3	1,9	1,4	0,5	-0,1	0,7	1,9	1,6	1,1	0,5	0,1	1,8	1,2	6,90
Š9	*D. a.*	2,4	3,5	0,9	1,4	2,1	3,7	0,9	1,5	0,8	2,9	2,2	3,1	0,8	1,0	1,6	0,8	7,33
T1	*D. c.*	1,6	2,3	0,4	0,4	2,0	2,0	0,6	0,5	0,6	2,5	1,9	2,3	0,4	0,4	1,7	1,2	5,56
T10	*D. t.*	1,5	2,5	0,5	0,6	1,4	2,5	0,4	0,9	0,3	0,9	1,3	2,4	0,6	0,7	1,4	0,7	5,01

T2	*D. t.*	1,4	1,9	0,4	0,6	1,4	2,2	0,4	0,9	0,2	1,3	1,5	2,4	0,5	0,4	1,4	0,8	5,00
T3	*D. t.*	1,5	2,3	0,3	0,9	1,4	2,6	0,4	1,0	0,4	0,8	1,8	2,9	0,6	1,9	1,4	0,8	5,02
T4	*D. t.*	1,7	2,0	0,4	0,4	1,4	1,7	0,4	0,3	0,5	1,4	1,4	2,3	0,5	0,6	1,5	0,9	5,28
T5	*D. c.*	1,3	3,2	0,4	1,9	1,5	2,8	0,5	1,4	0,5	2,3	1,5	2,0	0,7	0,3	1,7	0,9	5,73
T6	*D. z.*	1,7	3,2	0,4	1,4	1,6	3,0	0,5	1,5	0,5	1,5	1,5	3,0	0,7	1,6	1,9	1,2	5,40
T7	*D. t.*	1,4	2,0	0,5	1,2	1,3	2,7	0,3	1,4	0,3	1,1	1,6	2,6	0,7	0,7	1,3	0,7	5,12
T8	*D. t.*	1,5	2,3	0,3	0,8	1,5	2,3	0,4	1,1	0,3	0,9	1,7	2,3	0,5	0,8	1,3	0,8	5,09
T9	*D. c.*	1,5	3,3	0,5	1,3	1,5	2,9	0,5	1,3	0,5	1,0	1,6	3,1	0,8	1,3	1,5	0,7	5,28
TET 01	*D. a.*	1,9	2,1	0,6	-0,1	1,9	2,1	0,6	0,1	0,5	2,2	2,0	1,8	0,8	-0,1	2,5	2,6	7,36
TET 02	*D. a.*	1,5	1,4	0,5	-0,2	2,1	1,1	0,5	-0,3	0,6	2,4	1,7	1,6	0,8	-0,3	2,2	2,4	7,23
TET 03	*D. a.*	2,0	1,9	0,7	-0,3	1,8	1,7	0,4	0,1	0,7	2,2	1,6	1,7	0,8	-0,1	2,7	2,8	7,24
TET 04	*D. a.*	1,8	1,5	0,7	-0,3	1,8	1,4	0,5	-0,3	0,6	2,0	1,7	1,4	0,8	-0,2	2,1	2,1	7,29
VCE 01	*D. c.*	1,1	2,7	0,4	2,3	1,6	2,7	0,9	1,4	0,7	1,9	1,8	3,3	0,6	0,8	1,9	2,1	5,62
VCE 02	*D. c.*	1,2	2,6	0,5	1,5	1,5	3,0	1,0	1,4	0,8	2,0	1,8	3,0	0,7	1,0	2,1	2,2	5,54
VCE 03	*D. i.*	1,5	2,5	0,5	0,8	1,8	2,2	0,8	0,6	0,7	2,0	1,7	3,1	0,7	1,1	1,9	2,0	6,40
VCE 04	*D. i.*	1,4	2,9	0,5	1,3	2,1	2,5	0,7	1,2	0,7	2,2	1,6	3,1	0,9	1,7	2,2	2,5	6,35
VCE1	*D. c.*	0,8	3,1	0,5	1,8	1,4	2,9	0,8	1,6	0,8	2,4	1,3	3,1	0,6	1,6	3,0	2,3	5,71
VCE2	*D. i.*	1,6	3,6	0,6	1,7	2,0	3,1	1,0	0,9	0,7	2,5	1,9	3,0	0,9	1,4	3,2	2,2	6,24
VJ1	*D. c.*	0,9	3,3	0,3	2,1	1,9	3,1	0,6	1,6	0,9	4,0	1,1	2,6	0,4	1,4	1,3	0,9	5,55
VJ2	*D. c.*	1,0	3,3	0,4	1,9	1,9	3,3	0,9	1,7	0,8	3,6	1,3	2,5	0,5	1,2	1,4	0,9	5,48
VJ3	*D. c.*	0,9	2,6	0,3	1,4	1,6	2,1	0,9	1,6	0,8	3,2	1,3	2,2	0,4	0,6	1,4	1,1	5,35
VJ4	*D. c.*	1,2	2,6	0,5	1,3	1,8	2,6	0,7	1,4	0,8	2,9	1,4	2,5	0,5	0,9	2,4	1,9	5,49
VJ5	*D. c.*	1,3	3,1	0,4	1,7	2,0	3,1	1,0	1,0	0,8	2,9	1,1	2,9	0,7	1,6	1,9	1,2	5,50
VLT 01	*D. a.*	1,8	1,9	0,7	0,2	1,9	2,0	0,9	0,1	0,6	2,7	1,9	2,5	0,9	0,5	2,7	2,8	7,19
VLT 02	*D. a.*	1,5	1,7	0,5	0,0	1,9	1,4	0,8	-0,2	0,6	1,9	2,0	1,5	0,6	-0,4	1,9	1,9	7,15
VLT 03	*D. a.*	1,6	1,8	0,7	0,0	1,9	1,9	0,7	-0,1	0,6	2,5	1,9	1,7	0,7	0,1	2,1	2,2	7,37
VLT 04	*D. i.*	1,2	3,3	0,4	2,3	2,4	3,2	1,0	1,1	0,7	2,5	2,3	3,5	0,7	1,0	1,1	1,3	6,41
VLT 05	*D. a.*	2,3	2,7	0,7	0,4	2,4	2,8	0,9	0,5	0,8	2,0	2,0	2,5	0,8	0,4	1,0	1,0	7,20
VLT 06	*D. i.*	1,7	3,6	0,6	1,7	2,4	3,9	1,1	1,4	0,7	2,6	1,8	3,9	0,8	1,6	2,4	2,5	6,31
VLT 07	*D. c.*	1,4	4,5	0,5	2,6	2,3	4,6	1,0	2,5	1,0	3,1	1,3	4,4	0,4	2,6	1,4	1,4	5,91
VLT 08	*D. i.*	1,8	4,8	0,6	2,5	2,8	4,0	1,1	1,3	0,8	3,0	2,5	4,8	0,9	2,8	2,9	3,1	6,31
VÝSL 1	*D. c.*	1,0	2,6	0,6	1,2	1,5	2,0	0,5	0,8	0,7	1,8	1,6	2,3	0,6	0,7	2,1	1,5	5,32
VÝSL 2	*D. c.*	1,2	3,0	0,5	1,7	1,8	2,6	0,8	0,7	0,7	2,3	1,5	3,0	0,8	1,4	2,3	1,5	5,43
VÝSL 3	*D. c.*	1,0	2,9	0,5	1,2	1,9	2,5	0,8	0,5	0,8	2,2	1,6	2,9	0,9	1,1	1,9	1,0	5,36
Z1	*D. a.*	1,4	1,2	0,6	-0,1	1,5	1,0	0,0	0,7	0,7	2,0	0,8	1,3	0,7	0,2	1,7	1,0	7,43
Z2	*D. a.*	1,0	2,0	0,7	0,0	1,9	1,9	0,0	0,5	0,5	2,3	1,0	1,8	0,8	0,6	1,6	0,8	7,31
Z3	*D. a.*	1,7	2,2	0,5	0,6	2,2	2,5	0,0	0,2	0,8	2,4	1,1	2,2	0,6	0,6	1,7	1,1	7,50
Z4	*D. a.*	1,2	2,0	0,6	0,5	1,7	1,6	0,0	0,4	0,6	2,1	1,4	1,6	0,7	0,3	1,6	0,9	7,31
Z5	*D. a.*	1,4	2,4	0,7	0,5	2,1	2,3	0,0	0,2	0,7	2,6	1,4	1,9	0,8	0,6	1,9	1,0	7,39
Z6	*D. a.*	1,2	1,7	0,8	0,3	1,9	1,8	0,0	0,3	0,8	2,7	1,4	2,0	0,9	0,7	1,9	1,0	7,44
ZAD1	*D. a.*	2,2	2,7	0,8	0,1	2,7	2,8	1,0	-0,1	0,6	2,8	2,2	2,5	1,0	0,6	3,2	2,2	7,20
ZAD2_1	*D. i.*	2,4	3,3	0,8	0,8	2,7	3,2	3,6	0,7	0,7	2,6	2,4	3,4	1,0	0,8	2,8	1,8	6,38
ZAD2_10	*D. i.*	2,1	2,7	0,7	0,6	2,3	2,7	0,8	0,6	0,5	2,3	1,6	2,7	0,8	0,7	2,1	1,4	6,27
ZAD2_11	*D. c.*	0,9	2,2	0,5	1,0	1,4	2,0	0,7	0,8	0,6	2,0	1,3	2,0	0,8	0,9	2,0	1,1	5,65
ZAD2_12	*D. i.*	1,9	2,1	0,5	0,3	1,9	2,1	0,7	0,2	0,5	1,9	1,5	2,2	0,8	0,2	1,9	1,1	6,36
ZAD2_2	*D. i.*	2,7	3,6	0,7	0,9	2,8	3,3	1,1	0,9	0,8	3,2	2,4	3,4	0,9	1,2	3,5	2,6	6,28
ZAD2_3	*D. a.*	2,3	1,9	0,8	-0,3	2,0	2,1	0,6	-0,1	0,6	2,3	2,6	2,2	0,8	0,1	2,3	1,5	7,19
ZAD2_4	*D. i.*	1,9	2,8	0,6	0,7	2,0	2,5	0,8	0,6	0,7	2,2	1,8	2,4	0,9	0,3	2,5	1,6	6,48
ZAD2_5	*D. i.*	2,1	2,5	0,8	0,3	2,1	2,2	2,4	0,2	0,7	2,1	1,9	2,8	0,9	0,8	2,2	1,3	6,35
ZAD2_6	*D. c.*	0,9	2,8	0,5	1,4	1,7	2,0	0,7	0,7	0,5	2,1	1,6	2,8	0,7	1,7	2,3	1,5	5,67

ZAD2_7	*D. a.*	2,4	2,5	0,6	0,3	2,6	3,1	0,9	0,5	0,7	3,2	2,3	3,3	0,8	0,6	2,1	1,3	7,11
ZAD2_8	*D. c.*	1,1	2,8	0,5	1,8	1,6	2,3	0,7	1,0	0,7	2,1	1,3	2,8	0,7	1,2	2,3	1,7	5,46
ZAD2_9	*D. i.*	1,9	2,3	0,8	0,2	2,0	2,5	1,0	0,4	0,7	2,2	2,0	2,7	0,9	0,4	2,1	1,2	6,33
ZLI 01	*D. i.*	1,4	2,5	0,5	1,1	2,0	2,6	1,1	1,0	0,6	2,4	1,5	2,6	0,6	0,7	2,0	2,1	6,21
ZLI 02_1	*D. t.*	1,9	2,9	0,5	1,1	1,8	2,2	0,5	0,1	0,5	1,2	1,4	2,1	0,4	0,5	1,5	1,5	5,21
ZLI 02_2	*D. z.*	1,4	2,0	0,4	0,6	1,6	2,0	0,6	0,4	0,5	1,3	1,7	2,1	0,6	0,9	1,8	1,9	5,17
ZLI 03	*D. c.*	1,4	3,0	0,3	1,4	1,7	3,2	0,6	1,6	0,7	1,6	1,5	3,1	0,6	1,5	1,8	2,0	5,32
ZLI 04	*D. z.*	1,7	3,4	0,5	1,5	2,0	3,4	0,6	1,4	0,6	1,3	2,1	3,5	0,7	1,3	1,6	1,6	5,15
ZLI 05	*D. z.*	1,5	1,9	0,4	0,2	1,6	1,6	0,5	0,1	0,5	1,3	1,5	1,9	0,4	0,5	1,5	1,5	5,17
ZLI 06	*D. o.*	2,2	2,7	0,6	0,3	2,0	2,5	0,7	0,6	0,5	2,3	2,0	2,7	0,9	0,6	2,2	2,4	6,19
ZLI 07	*D. z.*	1,1	2,3	0,4	1,2	1,5	1,1	0,5	1,1	0,5	1,1	1,2	2,7	0,4	1,3	1,4	1,5	5,16
ZLI 08	*D. a.*	2,1	2,4	0,7	0,0	2,2	2,5	0,8	0,1	0,9	3,4	1,8	2,2	1,0	0,7	2,0	2,2	7,14
ZLI 09_1	*D. t.*	1,1	2,5	0,4	1,1	1,4	2,4	0,6	1,2	0,4	1,4	1,4	2,8	0,4	1,2	1,5	1,5	5,19
ZLI 09_2	*D. i.*	1,1	2,5	0,5	2,7	1,6	2,3	0,6	1,2	0,6	2,2	1,9	2,5	0,7	1,0	1,8	1,9	6,38
ZLI 10	*D. z.*	1,3	2,1	0,4	0,8	1,5	2,1	0,7	0,6	0,5	1,1	1,5	2,1	0,5	0,4	1,5	1,6	5,20
ZLI 11	*D. a.*	1,8	2,4	0,7	0,0	1,8	2,2	0,9	0,2	0,8	2,4	1,9	1,8	0,7	-0,1	1,7	1,8	7,32
ZLI 12	*D. a.*	1,8	2,7	0,4	1,1	2,0	3,0	1,0	0,5	0,8	0,6	1,5	2,7	0,4	0,7	2,1	2,1	7,61
ZLI 13	*D. o.*	2,0	2,8	0,6	0,7	2,0	2,6	0,6	0,5	0,6	1,5	1,5	2,7	0,7	1,4	1,8	1,9	6,02
ZLI 14	*D. c.*	0,8	2,5	0,4	1,5	1,2	2,4	0,7	1,1	0,6	1,9	1,3	2,6	0,5	1,3	1,8	1,9	5,52
ZLI 15_1	*D. z.*	1,2	3,0	0,5	1,5	1,4	3,0	0,6	1,5	0,6	1,2	1,5	2,9	0,7	1,0	1,7	1,8	5,21
ZLI 15_2	*D. t.*	1,7	3,1	0,3	1,8	1,6	3,1	0,5	1,1	0,5	1,4	1,4	2,4	0,5	0,9	1,5	1,6	5,13
ZMU 01	*D. a.*	1,9	1,6	0,7	-0,3	1,5	1,7	0,8	-0,1	0,8	2,0	1,9	1,4	0,8	-0,6	2,1	2,2	7,25
ZMU 02	*D. a.*	2,4	2,3	0,6	-0,1	2,1	2,3	0,7	0,2	0,8	2,2	2,0	2,3	0,8	0,4	1,4	1,5	7,19

Northern Europe

ID	*taxa*	v2	v3	v4	v5	v6	v7	v8	v9	v10	v11	v12	v13	v14	v15	v16	v17	GS (2C)
FIN 1/1	*D. z.*	1,3	2,8	2,4	1,3	1,4	2,6	0,6	1,1	0,5	1,5	1,5	2,6	0,8	1,2	1,9	1,0	5,24
FIN 1/2	*D. z.*	1,3	2,5	0,6	1,1	1,5	2,5	0,6	0,8	0,5	1,8	1,5	2,4	0,6	0,8	1,8	1,2	5,13
FIN 2	*D. c.*	1,4	2,1	0,5	0,7	1,7	2,5	0,6	0,8	0,5	2,1	1,6	2,6	0,6	0,7	2,0	1,3	5,36
FIN 3/1	*D. c.*	1,1	4,4	0,6	2,9	1,7	3,6	0,8	1,5	0,6	2,5	1,4	4,1	0,8	2,4	2,1	1,2	5,46
FIN 3/2	*D. c.*	0,9	2,7	0,4	2,1	1,2	3,4	0,7	1,9	0,7	2,3	1,3	3,0	0,7	1,5	2,1	1,4	5,4
FIN 4/1	*D. c.*	1,0	3,0	0,5	1,8	1,5	2,8	0,8	1,3	0,5	1,9	1,5	2,9	0,9	1,6	2,0	1,1	5,42
FIN 4/2	*D. c.*	1,1	2,4	0,5	1,1	1,4	2,4	0,6	0,9	0,6	1,5	1,3	2,6	0,9	0,6	1,8	0,9	5,22
FIN 5/1	*D. c.*	1,1	3,5	0,6	2,1	1,6	3,4	0,8	1,8	0,8	2,4	1,7	4,0	0,8	2,2	2,6	1,7	5,54
FIN 5/2	*D. c.*	5,3	3,1	0,5	1,6	1,9	3,2	0,9	1,6	0,9	2,9	1,4	3,0	0,8	1,4	2,8	2,0	5,49
FIN 5/3	*D. c.*	1,0	3,2	0,7	1,9	1,7	3,1	0,7	1,6	0,7	2,1	1,6	2,8	0,8	1,5	2,1	1,3	5,44
FIN 6/1	*D. c.*	1,5	2,0	0,6	0,4	1,4	2,0	0,6	0,3	0,5	1,5	1,1	1,6	0,9	0,6	1,4	0,5	5,47
FIN 6/2	*D. z.*	1,4	2,3	0,5	0,5	1,7	2,2	0,6	0,7	1,4	1,4	1,4	2,0	0,6	0,6	1,6	1,0	5,14
FIN 7/1	*D. c.*	1,2	2,2	0,6	0,9	1,7	2,2	0,7	0,8	0,6	2,0	1,4	2,3	0,8	0,8	2,2	1,3	5,4
FIN 7/2	*D. c.*	1,0	2,0	0,5	1,0	1,4	2,1	0,7	0,7	0,6	2,1	1,2	2,2	0,8	0,6	2,1	1,3	5,31
FIN 8/1	*D. c.*	0,9	2,9	0,6	2,4	1,6	2,7	0,8	1,5	1,0	2,5	1,6	2,7	0,9	1,3	2,2	1,3	5,43
FIN 8/2	*D. c.*	1,1	3,2	0,6	2,1	1,6	3,2	0,8	1,9	1,0	2,6	1,5	3,0	1,0	1,4	2,6	1,6	5,44
GPS21	*D. a.*	2,3	2,8	0,8	-0,5	2,9	2,2	0,5	0,5	1,6	2,1	2,9	2,1	1,1	-0,8	1,9	0,8	7,12
NOR B/1	*D. a.*	1,9	1,8	0,8	-0,2	1,7	1,7	0,5	0,1	0,7	1,6	2,5	2,1	1,0	-0,2	2,0	1,1	7,12
NOR B/2	*D. a.*	1,9	2,0	1,0	-0,1	1,3	1,7	0,5	-0,1	0,6	1,7	1,6	2,0	0,8	0,3	2,0	1,2	7,22
NOR C/1	*D. c.*	1,1	2,9	0,6	1,8	1,8	2,8	0,7	1,3	0,6	2,3	1,7	3,0	0,8	1,3	2,1	1,3	5,61
NOR C/2	*D. c.*	1,3	2,5	0,5	1,3	1,5	2,2	0,6	0,9	1,0	2,0	1,4	2,6	0,9	1,1	2,0	1,1	5,46
NOR C/3	*D. c.*	1,0	2,5	0,5	2,5	1,5	2,3	0,6	0,8	0,6	2,0	1,4	2,4	0,9	1,1	1,9	1,0	5,47
NOR1	*D. a.*	2,4	2,2	0,8	-0,2	2,4	2,5	0,8	0,1	0,7	2,5	1,9	2,2	0,9	0,2	2,2	1,4	7,11

NOR2	*D. a.*	2,1	1,5	0,6	-0,5	1,9	1,5	0,5	-0,3	0,7	2,0	1,9	1,5	0,8	-0,3	2,0	1,2	7,29
SC1	*D. a.*	2,1	2,3	0,8	-0,5	3,4	2,6	0,6	-0,1	1,7	2,2	1,6	2,6	0,9	-0,7	1,9	1,0	7,07
SC10	*D. a.*	2,2	2,2	1,0	-0,4	1,9	2,2	0,4	-0,5	1,2	2,2	1,6	2,0	0,9	-0,7	1,9	1,0	7,04
SC2	*D. a.*	3,4	2,8	1,0	-0,9	3,6	2,8	0,5	-0,8	1,2	1,9	3,3	2,9	1,1	-1,0	1,9	0,9	7,06
SC3	*D. a.*	1,1	1,3	0,8	-0,7	1,8	1,3	0,3	-0,4	1,3	2,4	2,6	1,4	0,8	-1,2	2,2	1,4	7,1
SC4	*D. a.*	2,4	2,6	0,8	-0,2	1,9	2,0	0,8	-0,2	0,7	2,1	2,9	2,2	0,8	-0,7	2,4	1,5	7,03
SC5	*D. c.*	0,8	2,1	0,2	1,1	1,6	2,4	0,5	0,9	0,7	2,8	1,1	2,2	0,7	-0,4	1,9	1,2	5,43
SC6/1	*D. c.*	1,4	3,5	0,8	1,9	1,8	4,2	1,0	2,7	1,3	2,2	1,8	3,9	1,1	-1,4	2,8	1,7	5,49
SC6/2	*D. c.*	1,2	2,3	0,5	1,3	2,1	2,6	0,8	0,8	0,9	2,5	0,9	2,7	0,8	-1,5	2,3	1,6	5,51
SC6/3	*D. c.*	1,9	2,9	0,5	1,2	1,8	2,4	0,7	0,9	1,2	2,5	1,2	2,8	0,7	-1,3	2,1	1,4	5,49
SC6/4	*D. c.*	1,4	2,7	0,6	0,8	2,0	2,3	0,8	0,8	0,9	3,0	1,2	2,3	1,0	-1,3	2,3	1,3	5,51
SC6/5	*D. c.*	1,6	3,4	0,7	1,4	1,7	3,9	0,8	1,9	0,6	2,3	1,4	3,3	0,8	-0,8	2,0	1,2	5,43
SC6/6	*D. c.*	1,1	2,2	0,5	1,3	1,8	2,1	0,7	-0,4	2,0	2,1	1,1	2,5	0,9	-1,2	2,3	1,5	5,42
SC6/7	*D. c.*	1,0	2,1	0,4	1,1	1,9	2,5	0,6	1,1	0,7	2,1	1,4	2,2	0,7	-1,3	1,8	1,0	5,44
SC6/8	*D. c.*	1,2	1,8	0,5	0,7	1,3	1,8	0,7	0,9	0,7	2,4	1,2	2,2	0,8	-1,5	2,2	1,4	5,5
SC7	*D. a.*	1,2	1,5	0,8	-1,2	2,1	1,4	0,3	-0,3	0,9	2,0	1,4	1,1	0,8	-0,6	2,0	1,3	7,08
SC8	*D. a.*	2,5	3,0	1,0	-0,9	2,5	3,0	0,4	0,3	0,7	2,1	2,3	3,2	0,9	-0,9	2,1	1,2	7,01
SC9	*D. a.*	2,6	2,6	0,8	-0,3	2,9	2,4	0,3	-0,1	1,1	2,1	1,7	2,6	0,8	-0,9	2,0	1,2	6,98
SK 1/1	*D. a.*	2,0	2,3	0,6	0,4	2,1	2,2	0,8	0,2	0,6	2,4	2,3	0,8	0,8	0,3	2,4	1,5	7,15
SK 1/2	*D. a.*	2,2	2,7	0,8	0,1	2,4	2,4	0,8	0,1	0,8	2,6	2,5	0,9	0,9	0,5	2,4	1,5	7,26
SK 1/3	*D. a.*	2,0	3,4	0,7	1,1	2,1	3,4	1,0	1,1	0,7	2,7	3,1	0,9	0,9	1,3	1,8	0,9	7,28
SK 2/1	*D. a.*	2,3	1,8	0,6	-0,4	2,0	1,7	0,7	-0,1	0,6	2,4	1,8	0,9	0,9	-0,1	2,3	1,4	7,18
SK 2/2	*D. a.*	2,2	2,4	0,8	-0,2	2,0	2,3	1,0	0,1	0,7	2,7	2,1	0,9	0,9	0,3	2,7	1,8	7,15
SK 3/C	*D. a.*	2,0	1,9	0,6	0,0	1,8	2,1	0,7	0,1	0,5	1,9	1,8	1,8	0,8	0,2	2,4	1,6	7,11
SK 3/D	*D. a.*	2,4	2,0	0,8	-0,5	1,8	2,1	0,8	0,0	0,7	2,7	1,6	2,0	0,8	0,1	2,7	2,0	7,06
SK 3A/1	*D. a.*	2,3	2,0	0,8	-0,3	1,8	1,9	0,8	-0,1	0,5	2,6	1,8	0,9	0,9	-0,1	2,5	1,5	7,33
SK 3A/2	*D. a.*	2,2	2,0	0,6	0,2	2,0	1,6	0,6	-0,1	0,7	2,0	1,7	1,7	0,9	-0,2	2,2	1,4	7,32
SK 3A/3	*D. a.*	2,2	2,4	0,8	0,9	2,1	2,8	0,6	0,8	0,6	1,6	1,8	3,2	0,8	0,9	1,4	0,6	7,2
SK 3B/1	*D. a.*	1,7	1,8	0,5	0,0	1,8	1,9	0,9	0,2	0,6	2,3	1,6	1,8	0,8	0,3	2,4	1,7	7,12
SK 4/2	*D. a.*	2,4	2,7	0,7	-0,1	2,5	2,4	1,1	0,1	0,7	2,5	2,0	2,4	1,0	0,5	2,6	1,6	7,28
SK 4/3	*D. a.*	2,0	2,3	0,8	-0,1	2,4	2,3	1,3	0,1	0,8	3,6	2,3	2,7	1,0	-0,1	3,7	2,7	7,11
SK 4/4	*D. a.*	2,0	1,9	0,7	-0,1	2,1	2,0	0,7	-0,1	0,6	2,6	1,9	1,8	0,7	-0,2	1,9	1,2	7,04
SK 4/5	*D. a.*	2,6	3,7	0,5	0,7	2,3	3,3	1,2	0,8	0,8	2,8	2,1	3,5	0,9	1,2	3,3	2,4	7,21
VB	*D. a.*	2,2	2,1	0,9	-0,1	2,3	1,9	0,8	0,1	0,7	3,3	2,1	1,0	1,0	-0,6	3,2	2,1	7,24
